# Effectiveness of a mindfulness and acceptance-based intervention for improving the mental health of adolescents with HIV in Uganda: An open-label trial

**DOI:** 10.1371/journal.pone.0301988

**Published:** 2024-05-09

**Authors:** Khamisi Musanje, Rosco Kasujja, Carol S. Camlin, Nic Hooper, Josh Hope-Bell, Deborah L. Sinclair, Grace M. Kibanja, Ruth Mpirirwe, Joan N. Kalyango, Moses R. Kamya

**Affiliations:** 1 Clinical Epidemiology Unit, Makerere University, Kampala, Uganda; 2 School of Psychology, Makerere University, Kampala, Uganda; 3 Department of Obstetrics, Gynecology & Reproductive Sciences, University of California, San Francisco, United States of America; 4 School of Psychology, Cardiff University, Wales, United Kingdom; 5 School of Medicine, Cardiff University, Wales, United Kingdom; 6 Department of Special Needs Education, Ghent University, Belgium; 7 School of Medicine, Makerere University, Kampala, Uganda; University of Nigeria, Nsukka, NIGERIA

## Abstract

Adolescents with HIV (AWH) face the double burden of dealing with challenges presented by their developmental phase while coping with stigma related to HIV, affecting their mental health. Poor mental health complicates adherence to daily treatment regimens, requiring innovative psychosocial support strategies for use with adolescents. We assessed the effectiveness of a mindfulness and acceptance-based intervention on the mental health of AWH in Uganda. One hundred and twenty-two AWH, mean age 17 ±1.59 (range 15 to 19 years), 57% female, receiving care at a public health facility in Kampala were enrolled in an open-label randomized trial (ClinicalTrials.gov: NCT05010317) with assessments at pre-and post-intervention. The mindfulness and acceptance-based intervention involved weekly 90-minute group sessions for four consecutive weeks facilitated by two experienced trainers. Sessions involved clarifying values, skillfully relating to thoughts, allowing and becoming aware of experiences non-judgmentally, and exploring life through trial and error. The control group received the current standard of care. Three mental health domains (depression, anxiety, and internalized stigma) were compared between the intervention and control groups. A linear mixed effects regression was used to analyze the effect of the intervention across the two time points. Results showed that the intervention was associated with a statistically significant reduction in symptoms of depression (β = -10.72, 95%CI: 6.25, -15.20; *p* < .0001), anxiety (β = -7.55, 95%CI: 2.66, -12.43; *p* = .0003) and stigma (β = -1.40, 95%CI: 0.66 to -2.15; *p* = .0004) over time. Results suggest that mindfulness and acceptance-based interventions have the potential to improve the mental health of AWH.

## Introduction

Globally an estimated 1.7 million adolescents live with HIV, of whom 1.5 million are in Sub-Saharan Africa [1,2]. In Uganda, 98,000 young people between the ages of 10 and 19 years were estimated to be living with HIV in 2021 [2]. Adolescents with HIV (AWH) face the double burden of challenges presented by their developmental phase (such as insecurity) and stigma related to living with HIV which may predispose them to mental health-related problems [3].

Given this dual burden, it is perhaps unsurprising that AWH report more mental health-related challenges than children and adults with HIV [4–7] and have a higher risk of psychiatric hospitalization in their lifetime as compared to HIV-negative adolescents [8]. In Uganda, approximately 45.8% of AWH experience depressive symptoms [5], 23.8% have psychiatric disorders, 12.4% have behavioral disorders (e.g. Attention-deficit hyperactivity disorder) and 14% are suicidal [9]. Depression, anxiety, and behavioral disorders have been reported as the main mental health challenges faced by AWH in Uganda [10], while HIV-related stigma and shame are identified as the leading contributors to poor mental health among this group [11]. Such poor mental health levels among AWH are problematic because they adversely affects HIV treatment outcomes [4], retention in care [12], and quality of life [13] and are often associated with lapsed adherence to antiretroviral therapy (ART) [11,12,14,15]. There is therefore an urgent need to develop treatment strategies that improve the mental health of this population. Although several psychosocial support strategies such as intensive adherence counselling [16], Suubi+ adherence [17], cognitive behavioral therapy [18], and many others are in use with AWH in Uganda, the persistent mental health gap among AWH warrants alternative and innovative approaches to supplement existing care services if the mental health of AWH is to be improved [19].

Previous research has found that treatment strategies that promote emotion regulation, behavioral adjustment, and self-acceptance help improve the mental health of adolescents [11,15,20]. Mindfulness and acceptance-based interventions (MABI), specifically Acceptance and Commitment Therapy (ACT) [21], promote emotion regulation, behavioral adjustment, and self-acceptance through values-guided living [22,23]. Considered a third wave of cognitive and behavioral therapies’ [24], ACT recognizes that unpleasant and painful experiences are inevitable but the relationship formed with such experiences and the desire to control pain results in behaviors that only maintain the same experiences [20]. Thus, the goal of ACT is to support the development of psychological flexibility, which is defined as the ability to live as a conscious human being, fully aware of inner struggle while deliberately pursuing meaningful goals [24]. Importantly, when Acceptance and Commitment Therapy interventions improve the psychological flexibility of young people, improved mental health outcomes are reported [25–28].

Recently, a developmentally appropriate ACT-based model has been developed for young people. The Discoverer-Noticer-Advisor-values model (DNA-V) uses ACT principles which are derived from mindfulness and acceptance practices to promote the wellbeing of young people [22,29]. DNA-V utilizes language, metaphors, play, and conversation materials to support the development of three functional classes of behavior that promote psychological flexibility among young people [30]. The *Discoverer* involves learning to explore the world through trial and error, the *Noticer* involves developing better awareness and self-awareness, and the *Advisor* involves becoming familiar with and developing a relationship with one’s inner voice [29]. When young people develop these three skills, they are more likely to respond to life challenges in ways that serve their values [29]. In an earlier related study, we engaged thirty stakeholders involved in HIV care and mental health support in Uganda to review the DNA-V manual, gauge the appropriateness of the intervention for use in Uganda and suggest adjustments to improve its social validity. Key adjustments made included; simplifying the language of the Discoverer-Noticer-Advisor-values model DNA-V manual, incorporating local stories and plays in therapy, using racially congruent visuals, adjusting therapy sessions from six to four to minimize clinic visits and adding cards that depict different emotion expression [31].

Implementing an evidence-based MABI, such as the DNA-v model, with a known mechanism of change (psychological flexibility), could address the unmet needs of appropriate psychosocial support for AWH in Uganda [32,33]. However, since evidence supporting the effectiveness of the DNA-v model has almost exclusively been generated from resource-rich contexts and not with AWH [28,34,35], producing context-specific evidence in a low-resource setting is vital in influencing policy and encouraging the intervention’s uptake in a new setting [19]. Additionally, such evidence must be derived from studies with strong designs, such as randomized trials, and with large numbers of participants, to clarify the potential benefits of MABIs for AWH [20,36].

In this study, we used an open-label randomized controlled trial to evaluate the effectiveness of the Discoverer-Noticer-Advisor-values model in improving the mental health of AWH in Uganda.

## Methods

### Study design and setting

We conducted a pre-post open-label randomized trial with individuals assigned to either a group-based DNA-v intervention or to the current standard of care procedures (SOC). Participants were recruited from Kisenyi Health Center iv (KHC), an urban public health facility located in the Kisenyi zone, Rubaga division of Kampala district. The KHC is administratively managed by the Kampala Capital City Authority and serves a catchment population of about one million people within the central business district, mainly traders, urban refugees, as well as slum dwellers. The KHC offers free HIV care services and serves approximately 590 AWH.

#### Participants

The participants were older adolescents receiving care at the KHC. Inclusion criteria were: 1) being 15–19 years old, 2) an HIV-positive diagnosis, 3) attending care at the participating study clinic, and 4) ability to speak and understand Luganda or English (the most commonly spoken languages in Kampala City). AWH with cognitive impairments or disabilities, those planning to move out of the catchment area during the study period, or those participating in an HIV care study were excluded. Recruitment of participants began on November 1^st^ 2022 and ended on December 3^rd^ 2022. AWH who were 18 years and above provided written informed consent while those below 18 years of age provided assent, with written consent provided by their caregivers. Caregivers were not treated as participants and did not provide study data. The consent form was in both Luganda and English languages and participants were given sufficient time to read or be read the form in their language of preference and to ask any questions. The consent/assent process was conducted in a private room at the clinic with a health care provider in presence to witness that participation was voluntary.

### Ethics approval

Ethics approval was granted by the Makerere University School of Medicine Research and Ethics Committee (Mak-SOMREC-2021-176), and the Uganda National Council for Science and Technology (HS1656ES). We further obtained administrative clearance from the Kampala Capital City Authority, the body that manages all public health centers in the city.

### Protocol and adaptation

Study methods and results followed the 2010 CONSORT checklist for reporting parallel group randomized trials “Text in S1 Text” [37]. The study protocol was registered on ClinicalTrials.gov (NCT05010317).

In order to contextualize DNA-V to the local population, we made minor deviations from the DNA-V manual. First, because the study served a population vulnerable to session load, we delivered four sessions of the intervention rather than six. Reducing sessions was previously recommended by local mental health experts at the DNA-V adaptation stage due to concerns regarding travel cost and logistical burdens in the context of other life activities [38]. In addition, experts noted that ACT is a flexible approach that can be adjusted to meet the needs of participants in a given context [39] and four session ACT based treatments have been tested and found effective [40]. To implement these adaptations, sessions one and two (Introduction and Discoverer) were merged into a single session and the same was done for sessions five and six (Values and Self as context). To reduce session time with the adapted schedule, we replaced lengthy narratives from the DNA-V manual with shorter plays and games carrying the same messages [31]. This adaptation was reviewed and approved by the developers of the DNA-V.

Second, while adolescents age 14 and above are considered eligible for DNA-V, our study enrolled participants ages 15 and above. This decision was undertaken to align with local definitions of older adolescents. The HIV clinic grouped adolescent according to the WHO categorization of adolescents[41], where 14 year-olds are grouped with young adolescents group (10–14 years) rather than the older adolescents group (15–19 years).

Finally, we conducted the study at one health center (KHC) not two as indicated in the original protocol. KHC had a sufficient number of participants to meet the sample requirements. A full study protocol is attached as part of other documents “Text in S3 Text”.

### Procedure

After obtaining administrative clearance from the city authority, the research team approached staff at the adolescent clinic at the KHC to recruit participants. Having been granted access to anonymous medical records, we screened for eligibility and the clinic staff contacted potential participants telephonically with information about the study. Adolescents who expressed interest in the study were scheduled for individual appointments to inform and obtain consent/assent which was followed by a group briefing and later baseline assessment. Participants were then randomly assigned to the intervention or control group using allocation sequences prepared by an external bio-statistician. Allocation concealment was done using sealed serialized brown opaque envelopes that were kept by the statistician.

#### Randomization

**Fig 1:**
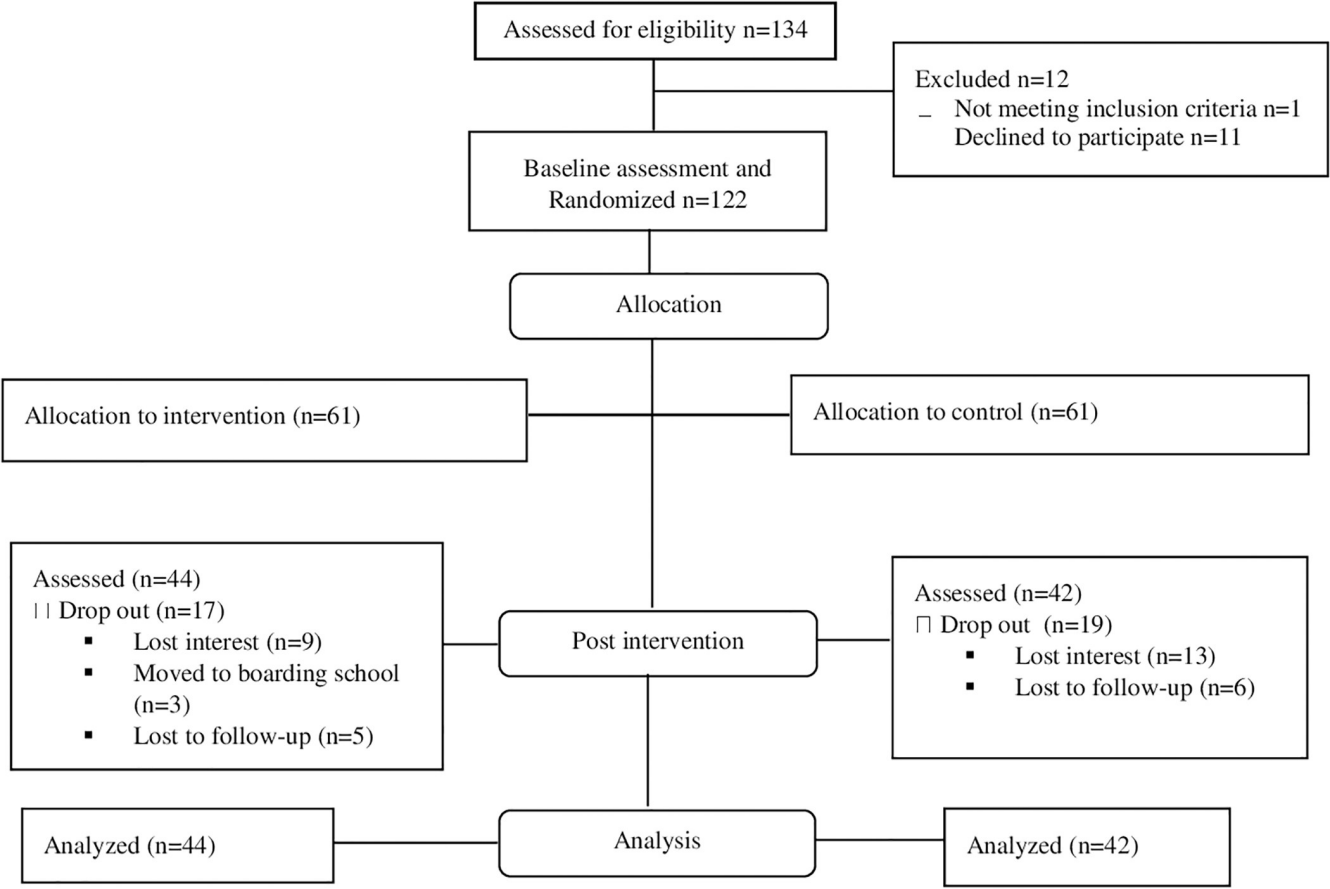
Flow diagram of the intervention process.

Participants were randomly allocated to the intervention or control group using computer-generated random numbers. The block randomization method was used using a STAT code for allocating AWH to the intervention or control groups and to achieve a 1:1 randomization ratio, we used a block size of six with equal individuals going to the two arms per block. Participants allocated to the intervention arm received the DNA-V training and the SOC, while participants who were allocated to the control group continued with the SOC only. Since participants were recruited from the same health center, there was a possibility of contamination. Thus, to minimize spillover, the research team worked closely with healthcare providers to schedule sessions on non-clinic days to reduce the possibility of unplanned meet-ups among participants, furthermore, the team also encouraged participants not to discuss the training with non-group members until it was completed. This was an open-label randomized study because healthcare providers (counsellors at the adolescent clinic) participated in recruitment, administration of the intervention, and the assessment of outcomes. The intervention flow process is shown in Fig 1 below,

### Study intervention

#### The discoverer-noticer-advisor-values model

The DNA-V intervention is a flexible mental health support program that targets to promote psychological flexibility among young people and can be delivered by lay providers such as counsellors and adolescent peers with minimal experience in facilitating group psychotherapy and working with adolescents. The providers are trained in using the DNA-V manual, practicing mindfulness, creating present moment awareness, values clarification and facilitating experiential exercises [30,31] and adherence to the guides of the manual ensures fidelity. In this study, group sessions of the DNA-V were delivered to AWH as life skills training responding to the developmental needs of young people [29]. To maximize the intervention effect, we divided participants in the intervention arm into sub-groups of 11–13 participants varied by age and gender to fit within an optimal group range of 8–16 participants recommended for ACT-based training [42]. After creating sub-groups, we designed a session schedule to guide the implementation of the intervention. The first author, who is experienced in group counselling for adolescents and had completed a six weeks course on using the DNA-v model which was organized by the model developers, and a one-month training on Acceptance and Commitment Therapy for teens and the second author, who is a clinical psychologist and trained in the therapeutic use of mindfulness and acceptance, collaboratively delivered four 90-minute sessions to each sub-group across four weeks, meeting each sub-group once a week at the clinic premises. We conducted measures pre- and post-intervention (at four weeks). Post-intervention assessment was conducted among AWH who completed at least 75% of the program (minimum of three out of the four sessions) which is considered minimum dosage for ACT-based programs [43]. Participants were reimbursed $10 (37,000 Uganda shillings) every week to compensate for their time and transport costs. The session content is detailed in Table 1 below.

**Table 1:** Content and activities of the DNA-V training with AWH.

Training session	Description of content
Session One	Overview of the DNA-V program and its main goal (life skills training that supports meaning-driven living, forming better relationships, and becoming who you want to be regardless of the situation). Self-introductions and bonding, agreeing to the group behaviors of respect and confidentiality, introducing the DNA-V model as a play.
	Values: identifying what brings meaning to you as a person (participants engaged in a values card sort exercise). Living a values-led life (small steps that matter). Navigating the challenges of living or struggling to fit in.
Session Two	The Noticer: normalizing the Noticer (games), naming emotions (using cards depicting emotions), practicing awareness, naming and describing emotions, and creating new rules for noticing.
Session Three	The Advisor: normalizing the inner voice (we all have an ‘advisor’ that keeps talking to us; games to become familiar with the advisor), appreciating the helpfulness of the inner voice, detaching from the inner voice (games to ‘unhook’), choosing when to listen to the inner voice and when not to depending on the situation. Creating new rules for relating with the inner voice (advisor).
Session Four	The Discoverer: trying, tracking, and building strength to serve values. Small steps build big action, reflecting and learning from what has worked and what has not.
	Putting D-N-A together while moving towards values: acting out the DNA with humour as we end the training.

#### The standard of care

The SOC involved a combination of clinic-based strategies routinely used to support AWH. The services are offered during adolescent clinic days or as weekend programs by trained healthcare providers or young adolescent peer leaders. SOC is an ongoing program recommended by the National Treatment Guidelines for the prevention and treatment of HIV in Uganda. For every clinic visit, an adolescent receives counselling services for about 30 minutes to an hour before meeting a doctor. The program structure is detailed and was designed by the Ministry of Health. The services are however varied between patients or groups depending on their level of adherence to antiretroviral therapy or reported mental health status. Services include psychosocial assessment (using the HEADSS tool for gathering information on home, education, eating, employment, activity, drugs, sex, sexuality, and suicidal ideation), and support (either individual or group-based, led by a healthcare worker to discuss disclosure of status, stigma, discrimination, treatment, loss and bereavement, and transitioning for AWH turning 18 years) [44].

Psychosocial support is combined with health education such as information about ART (available care packages, benefits, side effects, and managing lifelong treatment), information on growth and development such as sexual reproductive health (antenatal care, safe deliveries, and post-natal care), life skills education, counselling on substance use, nutrition services, pregnancy testing, and contraceptive methods [44].

### Measures and outcomes

We used *Beck’s Depression Inventory (BDI ii)* to measure symptoms of depression [45]. The BDI-ii is a 21-item self-report rating inventory that has been widely used to measure the presence and degree of depression among adolescents and adults. The BDI-ii includes both cognitive and somatic symptoms of depression. It has a high test-retest reliability (r = .93) and high internal consistency (α = .91) [45]. In scoring the BDI-ii, each of the 21 items is summed to give a single score. Each item has a 4-point scale ranging from 0–3, except items 16 and 18 which have seven options indicating either an increase or decrease in appetite and sleep. A total score in the range of 0–13 is considered minimal range, while 14–19 is mild, 20–28 is moderate and 29–63 is severe depression [45]. The cut-off guidelines for BDI-ii are adjusted based on the characteristics of the sample [46]. The BDI-ii has been validated among people with HIV in Uganda and shown good psychometric properties (α = .79) and (r = .86) [47].

We measured health-related anxiety using the *Short Health Anxiety Inventory (SHAI)* [48]. This is an 18-item measure of health-related anxiety independent of physical health status. It has commonly been used to assess health anxiety among adolescents and has good psychometric properties when used in non-clinical samples (α = .86) [49]. Responses from the instrument are scored on a 4-point Likert scale, where 0 = no symptoms, 1 = mild symptoms, 2 = severe symptoms and 3 = very severe symptoms. If more than one statement is selected, the higher-scoring statement is considered. The total score ranges between 0 to 54 with higher scores representing higher anxiety. A total score above 40.5 is considered to represent severe anxiety [50].

We measured stigma using the *Internalized AIDS-Related Stigma Scale (IARSS)* [51]. This is a 6-item scale reflecting self-defeating beliefs and negative perceptions of people living with HIV/AIDS (PLWHA) [51]. This instrument has been used and validated in the Ugandan context [52]. Items of the IARSS are assessed with a binary response of yes/no and a total scale score is computed as the sum of all the 6 items. Higher scores indicate higher levels of internalized HIV/AIDS-related stigma [51]. The IARSS has demonstrated acceptable internal consistency (α = .75), construct validity with measures of related constructs such as depression and mental health-related quality of life, and time stability when used in Uganda [52].

The study questionnaire “Text in S2 Text” was administered in either English or Luganda and the translation involved two bilingual native speakers of both languages. One translated the original English tool into Luganda and the other back-translated the Luganda version into English. The back-translated and original English tools were compared and inconsistencies were addressed. Both versions of the tool were then pilot tested with adolescents in another public health facility which was not part of the study. The scales had appropriate consistency: the BDI ii (α = .79, .80 for the English version and .85 for the Luganda version), the SHAI (α = .77; .89 for the English version and .74 for the Luganda version), and the IARSS (α = .80; .77 for the English version and .81 for the Luganda version).

### Statistical analysis

A sample size of 116 participants was set to ensure 80% power to detect at least a 4.09 decrease in mean outcome scores in the intervention versus the control arm of the trial. The following assumptions were considered in determining the sample size.

$$\text{N}=\frac{2{\left[\text{Z}\alpha +\text{Z}\beta \right]}^{2}\;{\text{S}}^{2}}{{\left(\mu 1-\mu 2\right)}^{2}}$$

where:

N is the sample size per group

Zα is the standard normal value corresponding to the level of significance = 1.96 for 5% level of significance

Zβ is the standard normal value corresponding to the power of the study = 0.84 for 80% power

μ1 is the mean in the control group and μ2 is the mean in the intervention group

μ1-μ2 = effect size (minimum meaningful difference between the means in the control and intervention groups)

S = standard deviation of the outcome in the control group

Considering a study evaluating the effectiveness of ACT on reducing depressive symptomatology among Swedish and Australian adolescents [28], the difference in means of the post-treatment depression scores between the ACT arm and control arm was 4.09 while the standard deviation in the control arm was 5.13 [28].

N = 25.1

26 participants in each group

If we consider a design effect of 2.0, the new minimum sample size is 26*(2) = 52. If we consider a 10% loss to follow-up, the revised sample size becomes (52*(1/ (1–0.10)) = 58 participants in each arm.

Baseline characteristics were compared using the Student’s t-test for continuous variables while the categorical variables were compared using the Fisher’s exact and Chi-square tests. To test the interaction effect of the intervention on the study outcomes over time, we controlled for the sub-grouping of participants. Specifically, intervention arm participants were assigned to each of the 5 treatment sub-groups of 11 to 13 participants each. Because the control group did not receive a group-based intervention, all control arm participants were assigned to a single treatment sub-group.

We used these sub-groupings in a hierarchical linear mixed effects model, using two time-points of pre-treatment and post-treatment. For comparison, we also performed a linear regression with score change as the outcome, controlling for treatment sub-group. Both analyses were repeated for the outcomes. Analyses were conducted using SPSS version 25.0 [53] “Data in S1 File” and STATA 17.0 and followed a per-protocol approach.

## Results

### Description of the study population

One hundred and twenty-two (n = 122) participants were randomly allocated to two study arms, (n = 61 in each study arm) and evaluated at two time points comparing the groups’ outcomes within times and between groups. The mean (standard deviation) age of study participants in the treatment and control arm was 17.0 (1.3) and 16.9 (1.6) respectively. In the treatment arm, 60.7% were females compared to 57.4% in the control arm. 54.1% of the study participants were in school both in the treatment arm and control arm as shown in Table 2 below. The mean age of the participants, sex, and school status did not differ significantly across groups at pre-assessment as shown in Table 2 below.

**Table 2:** Background characteristics of participants at enrollment into the trial.

Variable	Control (n = 61)	Intervention (n = 61)
**Age**, mean(±SD)	16.95(±1.59)	17.03(±1.28)
**Sex, n(%)**		
Female	35(57.38)	37(60.66)
Male	26(42.62)	24(39.34)
**School status n(%)**		
In school	33(54.10)	33(54.10)
Out of school	28(45.90)	28(45.90)

### Mental health outcomes

We compared the outcomes of depression, stigma, and anxiety in the intervention and control groups at pre- and post-intervention time points using a linear mixed effects model testing the interaction effect of group and time.

Results from the analysis found that the effect of treatment in the intervention arm compared to the control (SOC) arm was statistically significant after accounting for sub-groups for all three outcomes (depression, anxiety, and stigma). While with a linear regression looking at score change, we found that the change in depression and stigma scores were statistically significant after accounting for sub-grouping, but the change in anxiety score ceased to be significant after accounting for sub-grouping. Magnitudes of coefficients were similar in both analyses. Thus, a linear regression was dropped since a linear mixed effects model approach was more efficient, in that it allowed us to identify a statistically significant effect on anxiety score reduction, which would not have been identified in linear regression on score change. Results of the linear mixed effects model are presented below under the different mental health outcomes.

#### Depression

Looking at the interaction term between the time point (0 for pre-treatment, 1 for post-treatment) and randomization (intervention relative to control). The intervention was associated with a statistically significant reduction in depression. That is to say, there was a large reduction in depression score over time (decline by 10.72 points, 95% CI: 6.25–15.20 points, p<0.0001) as shown in Table 3 below.

**Table 3:** Showing the effects estimates for depression.

	B	SE	Df	t	Sig	95% CI
Intercept	18.95	1.84	116.12	10.29	< 2e-16 ***	
Time	1.15	1.65	79.00	0.70	0.48	
Randomization Experimental group	2.69	2.55	116.12	1.05	0.29	
Group #Time	-10.72	2.28	79.00	-4.69	1.1e-05***	-6.25, -15.20
Residual	5.27					

p < .0001***.

#### Anxiety

The result of the interaction term between the time point (0 for pre-treatment, 1 for post-treatment) and randomization (intervention relative to control) showed that the intervention was associated with a large reduction in anxiety score (decline by 7.55 points, 95% CI: 2.66, -12.43 points, p = 0.003) as shown in Table 4 below.

**Table 4:** Showing the effects estimates for anxiety.

	B	SE	Df	t	Sig	95% CI
Intercept	39.95	1.62	144.51	24.69	< 2e-16 ***	
Time	-2.66	1.79	83.00	-1.48	0.14	
Randomization Experimental group	-2.33	2.25	144.51	-1.04	0.30	
Group #Time	-7.55	2.49	83.00	-3.03	0.003***	2.66, -12.43
Residual	65.92					

p < .0001***.

#### Stigma

This result from the linear mixed effects model looking at the interaction term between the time point (0 for pre-treatment, 1 for post-treatment) and randomization (intervention relative to control) showed that the intervention was associated with significant reduction in stigma, that is to say, there was a large reduction in stigma scores (additional decline by 1.40 points, 95% CI: 0.66–2.15 points, p = 0.0004) as shown in Table 5 below.

**Table 5:** Showing the effects estimates for stigma.

	B	SE	Df	t	Sig	95%CI
Intercept	3.12	0.24	149.53	13.01	< 2e-16 ***	
Time	0.38	0.27	84.00	1.39	0.17	
Randomization Experimental group	-0.03	0.34	149.53	-0.08	0.93	
Group #Time	-1.40	0.38	84.00	-3.68	0.0004***	0.66, -2.15
Residual	1.56					

p < .0001***.

## Discussion

We compared the effect of a MABI (the Discoverer-Noticer-Advisor-values model-DNA-V) aimed at improving the mental health of AWH to a SOC control group. We hypothesized that the group-based DNA-v intervention would result in reduced symptoms of depression, HIV-related anxiety, and internalized stigma among older adolescents with HIV (AWH) aged 15–19 years based on prior data showing an association between mindfulness and acceptance strategies and the mental health of adolescents. Addressing mental health challenges among PLWHA has important treatment implications such as retention in care and adherence to daily medication. Overall, our findings show that the DNA-V intervention improved the mental health of AWH at post-assessment.

In line with our hypothesis, results from this study demonstrate that the DNA-V intervention was effective in reducing symptoms of depression and anxiety among AWH as compared to a control group who received the SOC only. Since MABIs promote psychological flexibility, it is possible that being open to life experiences non-judgmentally, developing self-compassion, and learning how to make choices in ways that bring meaning may be the mechanism by which participants in the intervention arm experienced less mental distress at post-intervention. Our findings are consistent with literature that emphasizes the direct and positive link between mindfulness and psychological well-being among adolescents [54]. The results are also consistent with previous studies examining the use of MABIs for improving the mental health outcomes of adolescents [28,55,56]. The results however contrast studies where MABIs showed no significant difference when compared to other mental health interventions for adolescents [57,58]. It should however be noted that some of these studies had small sample sizes which could have affected the results.

We used the DNA-V as a transdiagnostic approach since it had never been used to support the mental health of adolescents with HIV. Our findings showed beyond improving mental health, the DNA-V intervention reduced self-stigma around HIV. This may mean that the intervention has a preventive or protective component, as self-stigma can often compound mental health problems in AWH [9]. The results are in agreement with the literature suggesting that MABIs promote acceptance of HIV status which reduces emotional distress among PLWHA [59]. Furthermore, our findings are consistent with studies that underscore the relevance of MABIs in reducing stigma, anxiety and depression among PLWHA [36]. The results however contrast a recent study where an acceptance-based intervention did not reduce stigma manifestation among a sample of people living with HIV [60]. This study however had several limitations including being underpowered and delivering an intervention that had not been adapted to the study context [60]. Additional studies maybe needed to test this relationship further.

While several studies have tested the effectiveness of mindfulness and acceptance-related therapies in Uganda [61,62], such studies have focused on the mental health of adults in vulnerable situations. The present study is the first, to our knowledge, to examine and demonstrate, in a controlled trial, the effectiveness of an adolescent-centered MABI (the DNA-V model) for AWH in a low-income context (LIC). The study contributes to literature evaluating evidence-based mental health interventions for use with AWH, as a way of generating evidence to influence uptake and utilization [63]. The study is consistent with previous findings that emphasize the relevance of utilizing evidence-based interventions with AWH to support standardization and scalability of treatments in contexts where the development of new treatments is costly [64]. Additionally, the results reflect those from studies that have used other psychotherapeutic approaches to improve the mental health of AWH in LICs [15,65–67].

Our study had limitations and findings should be interpreted with caution. First, the study only collected data immediately following the intervention, thus, the timeframe between baseline and post-intervention was four weeks. It is therefore unclear whether the improvements found here would be sustained over a longer period. Randomized studies with longer follow-ups are needed to test the sustainability of the intervention effect. Nevertheless, pre-post studies are equally useful and provide evidence that lays the groundwork for larger and longer studies. Second, our intervention is only compared to a SOC group not with other interventions. Although the participants in the control condition were unlikely to have realized that they were not receiving the intervention, the inclusion of a third active control group or an additional intervention approach (e.g. adherence-based counselling) would allow comparison of our intervention to other AWH interventions. Whilst a single control group still provides a useful comparison because the group continued receiving the care that is currently offered to AWH, future similar studies might include active controls. Further still, the anxiety scale (SHAI) has not been validated in Uganda and among people with HIV which is likely to have some psychometric implications. Relatedly, the study based on self-reported data which is subject to social desirability bias. Participants could have given responses they thought the researchers were interested in. However, since the study involved repeated measures at different time intervals, such bias could have been minimized.

Another limitation of the study is that interclass correlation coefficients were not included in power calculations when estimating the sample size yet individuals were randomly assigned to study conditions and treatment administered in groups which is likely to induce potential correlation among observations within conditions and also possibly under power the study. It is however important to note that since the study groups were small in size, the variance inflation factor is presumed to be too small to change the study findings and secondly, using mixed effects methods in analysis helped to account for within group correlation [68].

Finally, the results may have been affected by the high rate of attrition (30% post-assessment), given that more vulnerable participants may have dropped out. However, since groups were almost equivalent at baseline and attrition did not differ significantly between conditions at post-assessment and the characteristics of those who dropped out were not different from those who completed, the possibility of affecting results is minimal. Nevertheless, smaller numbers in randomised conditions could still provide useful insights. Taken together, the study provides a starting point for future studies with larger samples to generate more evidence.

Despite these limitations, the study also has several strengths. The study focuses on a population that is worthy of great attention and care, particularly in LICs [10,12], and currently, there is very little research investigating psychological interventions to improve the mental health of these young people [64]. Secondly, the study responds to a call to evaluate evidence-based psychosocial support interventions across varying contexts to generate evidence that can influence the utilization of such interventions and influence policy [19]. This study precedes an adaptation phase where we engaged local health care providers and AWH from a different study site to culturally translate the DNA-v to improve its appropriateness for use in Uganda. In this process, we replaced metaphors and aids that relied on the internet and technology which could not be sustained in a resource-constrained setting, introduced cards that represent various emotional states to aid emotional expression, incorporated story telling which is a famous therapeutic practice in Uganda, revised the language of the manual and introduced racially congruent visuals [31]. We further conducted qualitative interviews among participants to gauge acceptability and feasibility of DNA-v before evaluation [38]. Thus, we evaluated an intervention that was already deemed feasible for use in Uganda. The research study has also used a rigorous randomized controlled design, meaning that the risk of bias can be considered low. Finally, the strengths of the intervention is that the DNA-v is an evidence-based approach [69] and that the specific materials have been adapted to the Ugandan culture and context [31].

## Conclusion

To conclude, AWH are a clinical population that faces many medical, psychological, and social challenges. As they represent the future generation, it is vital to ensure that these individuals can access effective psychological support. The results in this study demonstrate that group DNA-v interventions are effective and therefore represent such a source of support. Although there may be certain barriers to access, the intervention studied here can be considered feasible due to its group format and time efficiency. However, further larger-scale studies with increased sample size and multiple test sites are needed to help strengthen the evidence. Further still, translating the DNA-V into real life clinical practise will require integration into existing standard of care, involving peer leaders to lessen the burden on providers, fitting sessions into pre-existing scheduled activities such as drama clubs and running DNA-V sessions on clinic days to reduce on the frequency of clinic visits as earlier identified in our previous studies [31,38].

## Supporting information

S1 File Data file. Data which supported the study conclusions.
(XLSX)

S1 Text CONSORT checklist. Guidelines followed in reporting clinical trial data.
(DOCX)

S2 Text Study questionnaire. Instrument measuring depression symptoms, HIV related anxiety and stigma.
(DOCX)

S3 Text Clinical trial NCT05010317 protocol. Detailed clinical trial information.
(PDF)
