# Supplementary material for: Effectiveness of a mindfulness and acceptance-based intervention for improving the mental health of adolescents with HIV in Uganda: An open-label trial
Source: PLoS One. 2024 May 9;19(5):e0301988. doi: 10.1371/journal.pone.0301988 (PMC11081388; doi:10.1371/journal.pone.0301988)
Supplement: S2 Text — Instrument measuring depression symptoms, HIV related anxiety and stigma. (DOCX) [file pone.0301988.s003.docx]

Study identification no:………………

**ACT** *for* **Adolescents Study**

**Research Questionnaire**

Dear participants, this questionnaire is intended to serve research purposes only. Responses given will be kept confidential and cannot in any way be used against you. Kindly respond in the most honest way possible.

Beck’s Depression Index (BDI-ii)

Please read each group of statements carefully. And then pick out the one statement in each group that best describes the way you have been feeling during the past two weeks, including today. Circle the number beside the statement you have picked. Be sure that you do not choose more than one statement for any group, including Item 16 (Changes in Sleeping Pattern) or Item 18 (Changes in Appetite).

1. Sadness

0. I do not feel sad.

1. I feel sad much of the time.

2. I am sad all the time.

3. I am so sad or unhappy that I can't stand it.

2. Pessimism

0. I am not discouraged about my future.

1. I feel more discouraged about my future than I used to.

2. I do not expect things to work out for me.

3. I feel my future is hopeless and will only get worse.

3. Past Failure

0. I do not feel like a failure.

1. I have failed more than I should have.

2. As I look back, I see a lot of failures.

3. I feel I am a total failure as a person.

4. Loss of Pleasure

0. I get as much pleasure as I ever did from the things I enjoy.

1. I don't enjoy things as much as I used to.

2. I get very little pleasure from the things I used to enjoy.

3. I can't get any pleasure from the things I used to enjoy.

5. Guilty Feelings

0. I don't feel particularly guilty.

1. I feel guilty over many things I have done or should have done.

2. I feel quite guilty most of the time.

3. I feel guilty all of the time.

6. Punishment Feelings

0. I don't feel I am being punished.

1. I feel I may be punished.

2. I expect to be punished.

3. I feel I am being punished.

7. Self-Dislike

0. I feel the same about myself as ever.

1. I have lost confidence in myself.

2. I am disappointed in myself.

3. I dislike myself.

8. Self-Criticalness

0. I don't criticize or blame myself more than usual.

1. I am more critical of myself than I used to be.

2. I criticize myself for all of my faults.

3. I blame myself for everything bad that happens.

9. Suicidal Thoughts or Wishes

0. I don't have any thoughts of killing myself.

1. I have thoughts of killing myself, but I would not carry them out.

2. I would like to kill myself.

3. I would kill myself if I had the chance.

10. Crying

0. I don't cry any more than I used to.

1. I cry more than I used to.

2. I cry over every little thing.

3. I feel like crying, but I can't.

11. Agitation

0. I am no more restless or wound up than usual.

1. I feel more restless or wound up than usual.

2. I am so restless or agitated, it's hard to stay still.

3. I am so restless or agitated that I have to keep moving or doing something.

12. Loss of Interest

0. I have not lost interest in other people or activities.

1. I am less interested in other people or things than before.

2. I have lost most of my interest in other people or things.

3. It's hard to get interested in anything.

13. Indecisiveness

0. I make decisions about as well as ever.

1. I find it more difficult to make decisions than usual.

2. I have much greater difficulty in making decisions than I used to.

3. I have trouble making any decisions.

14. Worthlessness

0. I do not feel I am worthless.

1. I don't consider myself as worthwhile and useful as I used to.

2. I feel more worthless as compared to others.

3. I feel utterly worthless.

15. Loss of Energy

0. I have as much energy as ever.

1. I have less energy than I used to have.

2. I don't have enough energy to do very much.

3. I don't have enough energy to do anything.

16. Changes in Sleeping Pattern

0. I have not experienced any change in my sleeping.

1a. I sleep somewhat more than usual.

1b. I sleep somewhat less than usual.

2a. I sleep a lot more than usual.

2b. I sleep a lot less than usual.

3a. I sleep most of the day.

3b. I wake up 1-2 hours early and can't get back to sleep.

17. Irritability

0. I am not more irritable than usual.

1. I am more irritable than usual.

2. I am much more irritable than usual.

3. I am irritable all the time.

18. Changes in Appetite

0. I have not experienced any change in my appetite.

1a. My appetite is somewhat less than usual.

1b. My appetite is somewhat greater than usual.

2a. My appetite is much less than before.

2b. My appetite is much greater than usual.

3a. I have no appetite at all.

3b. I crave food all the time.

19. Concentration Difficulty

0. I can concentrate as well as ever.

1. I can't concentrate as well as usual.

2. It's hard to keep my mind on anything for very long.

3. I find I can't concentrate on anything.

20. Tiredness or Fatigue

0. I am no more tired or fatigued than usual.

1. I get more tired or fatigued more easily than usual.

2. I am too tired or fatigued to do a lot of the things I used to do.

3. I am too tired or fatigued to do most of the things I used to do.

21. Loss of Interest in Sex

0. I have not noticed any recent change in my interest in sex.

1. I am less interested in sex than I used to be.

2. I am much less interested in sex now.

3. I have lost interest in sex completely.

Short health anxiety Inventory (SHAI)

The following questions have four responses, read each group of responses carefully and circle what most describe your feelings in the last one week. If more than one statement applies to your, tick all those you feel are applicable.

1.Health concern

(a) I do not worry about my health

(b) I occasionally worry about my health

(c) I spend much of my time worrying about my health

(d) I spend most of my time worrying about my health

2. Perception of pains in comparison to people of the same age.

(a) I notice aches / pains less than most other people (of my age)

(b)I notice aches / pain are much as most other people (of my age)

(c) I notice aches/ pains more than most other people (of my age)

(d) I am aware of aches / pains in my body all the time

3. Awareness of bodily sensations/changes.

(a) As a rule I am not aware of bodily sensations or changes

(b) Sometimes I am aware of bodily sensations or changes

(c) I am often aware of bodily sensations or changes

(d) I am constantly aware of bodily sensations or changes

4. Ability to resist thoughts of illness

(a) Resisting thoughts of illness is never a problem

(b) Most of the time I can resist thoughts of illness

(c) I try to resist thoughts of illness but am often unable to do so

(d) Thoughts of illness are so strong that I no longer even try to resist them

5. Fear of having serious illness

(a) As a rule I am not afraid that I have a serious illness

(b) I am sometimes afraid that I have a serious illness

(c) I am often afraid that I have a serious illness

(d) I am always afraid that I have a serious illness

6. Imagine being sick

(a) I do not have images (mental pictures) of myself being ill

(b) I occasionally have images of myself being ill

(c) I frequently have images of myself being ill

(d) I constantly have images of myself being ill

7. Ability to take mind off health thoughts

(a) I do not have any difficulty taking my mind off thoughts about my health

(b) I sometimes have difficulty taking my mind off thoughts about my health

(c) I often have difficulty in taking my mind off thoughts about my health

(d) Nothing can take my mind off thoughts about my health

8. Relieved if doctor says nothing’s wrong.

(a) I am lastingly relieved if my doctor tells me there is nothing wrong

(b)I am initially relieved but the worries sometimes return later

(c) I am initially relieved but the worries always return later

(d) I am not relieved if my doctor tells me there is nothing wrong

9. Hear about an illness makes me think I can have it.

(a) If I hear about an illness I never think I have it myself

(b)If I hear about an illness I sometimes think I have it myself

(c) If I hear about an illness I often think I have it myself

(d) If I hear about an illness I always think I have it myself

10. Wonder what body sensations/changes mean.

(a) If I have a bodily sensation or change I rarely wonder what it means

(b)If I have a bodily sensation or change I often wonder what it means

(c) If I have a bodily sensation or change I always wonder what it means

(d) If I have a bodily sensation or change I must know what it means

11. Perceived risk of getting a disease

(a) I usually feel at very low risk for developing a serious illness

(b) I usually feel at fairly low risk for developing a serious illness

(c) I usually feel at moderate risk for developing a serious illness

(d) I usually feel at high risk for developing a serious illness

12. Belief of having a serious illness

(a) I never think I have a serious illness

(b) I sometimes think I have a serious illness

(c) I often think I have a serious illness

(d) I usually think that I am seriously ill

13. Ability to think of other things if I notice unexplained body sensation/changes.

(a) If I notice an unexplained bodily changes I don’t find it difficult to think about other things.

(b) If I notice an unexplained bodily change I sometimes find it difficult to think about other things

(c) If I notice an unexplained bodily change I often find it difficult to think about other things

(d) If I notice an unexplained bodily change I always find it difficult to think about other things.

14. Family/friends say I worry about my health

(a) My family / friends would say I do not worry enough about my health

(b) My family / friends would say I have a normal attitude towards my health

(c) My family / friends would say I worry too much about my health

(d) My family / friends would say I have extreme worry about my health

15. Ability to enjoy life even with a disease

(a) Even with a serious illness I still enjoy things in my life quite a lot

(b) Even with a serious illness I am still be able to enjoy things in my life a little

(c) With this serious illness I am almost completely unable to enjoy things in my life

(d) With this serious illness, I am completely unable to enjoy my life at all.

16. Chance of medical cure if have an illness.

(a) There is a good chance that modern medicine would be able to cure me

(b) There is a moderate chance that modern medicine would be able to cure me

(c) There is a very small chance that modern medicine would be able to cure me

(d) There is no chance that modern medicine would be able to cure me.

17. Illness would ruin aspects of life

(a) A serious illness would ruin some aspects of my life

(b) A serious illness would ruin many aspects of my life

(c) A serious illness would ruin almost every aspect of my life

(d) A serious illness would ruin every aspect of my life

18. Loss of dignity with an illness

(a) Having an illness does not make me feel that I had lost my dignity

(b) Having an illness makes me feel that I lost a little of my dignity

(c) Having an illness makes me feel that I lost quite a lot of my dignity

(d) Having an illness makes me feel that I totally lost my dignity

Avoidance and Fusion Questionnaire AFQ-Y8

We want to know more about what you think, how you feel, and what you do. Read each sentence. Then, circle a number between 0-4 that tells how true each sentence is for you.

| Question | Not at all True | A little True | Pretty True | True | Very True |
| --- | --- | --- | --- | --- | --- |
| 1. My life won’t be good until I feel happy. | 0 | 1 | 2 | 3 | 4 |
| 1. My thoughts and feelings mess up my life. | 0 | 1 | 2 | 3 | 4 |
| 1. The bad things I think about myself must be true. | 0 | 1 | 2 | 3 | 4 |
| 1. If my heart beats fast, there must be something wrong with me. | 0 | 1 | 2 | 3 | 4 |
| 1. I stop doing things that are important to me whenever I feel bad. | 0 | 1 | 2 | 3 | 4 |
| 1. I do worse in school when I have thoughts that make me feel sad. | 0 | 1 | 2 | 3 | 4 |
| 1. I am afraid of my feelings. | 0 | 1 | 2 | 3 | 4 |
| 1. I can’t be a good friend when I feel upset. | 0 | 1 | 2 | 3 | 4 |

Morisky Medication Adherence Scales: MMAS‐8

For this section, **Tick** inside the box that best explains your current situation.

| 1. | Do you sometimes forget to take your pills/medicine? | Yes | No |
| --- | --- | --- | --- |
| 2. | People sometimes miss taking their medications for reasons other than forgetting. Thinking over the past two weeks, were there any days when you did not take your medicine? | Yes | No |
| 3. | Have you ever cut back or stopped taking your medicine without telling your doctor because you felt worse when you took it? | Yes | No |
| 4. | When you travel or leave home, do you sometimes forget to bring along your medicine? | Yes | No |
| 5. | Did you take all your medicine yesterday? | Yes | No |
| 6. | When you feel like your symptoms are under control, do you sometimes stop taking your medicine? | Yes | No |
| 7. | Taking medicine every day is a real inconvenience for some people. Do you ever feel hassled about sticking to your treatment plan? | Yes | No |
| 8 | How often do you have difficulty remembering to take all your medicine? | Never/rarely  Once in a while  Sometimes  Usually  All the time | |
| **Total scores** | |  | |

Internalized Aids related Stigma Scale

For this section, **Tick** inside the box that best explains your situation.

| 1. | It is difficult to tell people about my HIV infection | Yes | No |
| --- | --- | --- | --- |
| 2. | Being HIV positive makes me feel dirty | Yes | No |
| 3. | I feel guilty that I am HIV positive | Yes | No |
| 4. | I am ashamed that I am HIV positive | Yes | No |
| 5. | I sometimes feel worthless because I am HIV positive | Yes | No |
| 6. | I hide my HIV status from others | Yes | No |
| **Total scores** | |  | |

Age; ………………………Gender; ……………………………. Health center; ……………………………Class if in school…………………

Study identification number; ……………………………………………………………….

END.

Thank you for your time.
