# Supplementary material for: Effectiveness of a mindfulness and acceptance-based intervention for improving the mental health of adolescents with HIV in Uganda: An open-label trial
Source: PLoS One. 2024 May 9;19(5):e0301988. doi: 10.1371/journal.pone.0301988 (PMC11081388; doi:10.1371/journal.pone.0301988)
Supplement: S3 Text — Detailed clinical trial information. (PDF) [file pone.0301988.s004.pdf]

Acceptability, feasibility and effectiveness of a mindfulness and acceptance-based intervention on improving the mental health of adolescents on antiretroviral therapy in Kampala.

By: Khamisi Musanje,

2020/HD07/21429U

Supervisors

Professor Moses Kamya

Dr. Rosco Kasujja

Professor Carol Camlin

A doctoral research proposal submitted to the school of medicine research and ethics committee (SOMREC), for ethics approval.

Version 2

Table of Contents

Abstract 5

Background 5

Purpose 5

Methods 5

Utility of findings 5

List of abbreviations 6

Operational Definitions 7

Introduction 8

Problem statement 10

Justification 11

Theoretical Framework 12

Conceptual Model 13

Research Questions 14

Purpose Statement 14

Research Objectives 14

Hypotheses. 14

Study period 14

Literature review 15

Introduction ..... **Error! Bookmark not defined.**

HIV among adolescents 15

Role of ART in treatment of HIV 16

Adherence to ART among adolescents 16

Factors associated with adherence to ART among adolescents 17

Interventions to support adherence to ART among adolescents. 23

Psychosocial interventions 23

Mindfulness and acceptance based support interventions for adolescents 25

Methods 30

Overall Study Design 30

Study Setting 31

Sub-studies 31

Sub-study One: Qualitative study. 31

Eligibility Criteria for sub-study 1 33

Sampling for sub-study 1 33
Sample size 33
Sampling strategy 33
Data collection 33
Domains of Inquiry 34
Data analysis 34
Sub-study Two: Quantitative study 34
Sub-study design. 34
Randomization 35
Allocation concealment 35
Allocation implementation 35
Blinding: 35
Population for sub-study Two 35
Eligibility criteria 35
Sampling for sub-study two 36
Sampling method 37
Data collection and measures 37
Study Schema 38
Study instruments 38
Study intervention 39
ACT-DNA-v 39
Outcome measures. 40
Statistical analysis 41
Schedule of activities 42
Intervention fidelity 42
Ethical Approval 43
Confidentiality and Privacy 43
Consent procedure and documentation 43
Strategies for retaining participants in the trial 44
Data Safety and Monitoring Committee (DSMC) 44
Community Engagement Plan 44
COVID-19 Risk Management Plan 45
Publication Plan 45

References 46
Appendix .... **Error! Bookmark not defined.**
Study tools .... **Error! Bookmark not defined.**
Research Questionnaire (English version) .... **Error! Bookmark not defined.**
Interview guide (Health Care Providers) English version .... **Error! Bookmark not defined.**
Interview guide (Users-Adolescents) English version .... **Error! Bookmark not defined.**
Participant consent form (English-Qualitative study) .... **Error! Bookmark not defined.**
Participant consent Form-English version (Quantitative study) .... **Error! Bookmark not defined.**
Translated forms (Luganda Versions) .... **Error! Bookmark not defined.**
Study Questionnaire (Luganda Version) .... **Error! Bookmark not defined.**
Ekibuuzo .... **Error! Bookmark not defined.**
Kituufumu katono .... **Error! Bookmark not defined.**
Interview Guide for Users (Luganda Version) .... **Error! Bookmark not defined.**
Participant Consent form (Luganda Version)-Qualitative Study .... **Error! Bookmark not defined.**
Participant Consent form Luganda Version-Quantitative Study. .... **Error! Bookmark not defined.**

Abstract

Background

Adolescents represent a growing share of people living with HIV in sub-Saharan Africa (SSA), yet they have lower engagement in care, poor adherence to medication and viral suppression (VS) compared to adults due to a high mental health burden they experience. We postulate that to achieve optimal adherence, interventions that promote mental health and are tailored to the dynamic social and cognitive needs of adolescents as they pass through life stages need to be tested and promoted. Mindfulness and acceptance-based interventions are slowly gaining traction as appropriate for adolescents.

Purpose

This study adapts and explores the acceptability of mindfulness and acceptance-based psychosocial intervention (acceptance and commitment therapy-ACT-Discoverer, noticer, advisor-values model-DNA-v), among providers (health care practitioners -HCPs) and users (adolescents living with HIV/AIDS). Further, it endeavours to measure the feasibility and effectiveness of ACT-DNA-v in reducing psychosocial barriers /mental health challenges (anxiety, depression & stigma) among adolescents living with HIV (ALWHAs). The study will be carried out at two Kampala Capital City Authority-KCCA health centres (Kisenyi and Kitebi) with ALWHA and their HCPs.

Methods

Sequential multi-methods design will be used, commencing with a formative phase where ACT-DNA-v will be adapted to fit the specific needs of users and providers in Kampala and using semi-structured interviews, explore the acceptability of ACT-DNA-v among users and providers, as well as implementation related factors. This phase will be followed by measuring the feasibility and effectiveness of the adapted ACT-DNA-v in improving mental health outcomes among ALWHA with a randomized control trial (RCT). The trial will involve quantitative surveys at baseline, post-intervention and follow-up, testing the effects of the intervention on process and clinical outcomes among ALWHA. Thematic analysis that involves coding, analysis and interpretation of findings utilizing qualitative data analysis software (QDA) will be used for the qualitative study, while T-test, Fisher's exact, regression, Chi-square tests and analysis of variance (ANOVA) respectively will be used in the quantitative phase to ascertain average mean differences between the two study groups on the outcome parameters of depression symptoms, anxiety, and stigma at pre-post intervention and psychological flexibility at follow-up.

Utility of findings

Study findings will be used to justify the integration of mindfulness and acceptance into psychosocial support services offered to adolescents living with HIV.

List of abbreviations

ACT: Acceptance and Commitment Therapy

AFQ-Y: Avoidance and Fusion Questionnaire for youth

AIDS: Acquired Immunodeficiency Syndrome.

ALWHA: Adolescents Living with HIV/AIDS

ANCOVA: Analysis of Covariance

ART: Antiretroviral Therapy.

CBTs: Cognitive Behavioral Therapies

DNA-v: Discoverer, Noticer and Advisor-values model

FGDs: Focus Group Interviews

HBM: Health Belief Model

HCPs: Health Care Providers (Counselors)

HIV: Human Immunodeficiency Virus.

IARSS: Internalized AIDS-Related Stigma Scale

IDI: In-Depth Interviews

KCCA: Kampala Capital City Authority

KHCIV: Kisenyi Health Center IV

KTHCIII: Kitebi Health Center iii

RFT: Relational Frame Theory

SAS-A-SF: Social anxiety scale for adolescents, short version.

SOM-REC: School of Medicine Research and Ethics Committee.

SSA: Sub-Saharan Africa

UNCST: Uganda National Council for Science and Technology.

VLS: Viral Load Suppression

Operational Definitions

ACT: Third wave cognitive behavioral therapy that combines mindfulness, acceptance, values clarification and behavioral goal setting to improve wellbeing through a process called psychological flexibility. ACT fosters psychological flexibility through six sub-processes; experiential acceptance, contact with the present moment, defusion, self-as-context, values, and committed action [1].

DNA-v: An evidence-based model that promotes psychological strength, values consistent living, vitality, and success. It is designed to help young people cope with challenges, stress, and change. DNA-V is a combination of Acceptance and Commitment Therapy (ACT) and positive psychology, and is underpinned by contextual behavioral science [2].

Adolescent: The world Health Organization (WHO) defines an adolescent as someone within the age range of 10 years – 19 years. For this particular study, we shall focus on older adolescents 15 – 19 years of age [3].

Psychological Flexibility: Contacting the present moment fully as a conscious human being, and based on what the situation affords, changing or persisting in behavior in the service of chosen values[4].

Cognitive fusion: Process that involves attaching a thought to an experience. This results in entanglement and believing thoughts as if they are real. The mindfulness and acceptance intervention is targeted at creating a detachment between thoughts and experiences [4].

Mindfulness: Being intensely aware of what you're sensing and feeling in the moment, without interpretation or judgment [7].

Acceptability: Exploring perceptions and experiences of providers (HCPs) and users (adolescents) regarding the intervention. Exploration will be done following the 7 domains of the theoretical framework for acceptability (TFA; affective attitude, burden, perceived effectiveness, ethicality, intervention coherence, opportunity cost and self-efficacy [8]. Narratives from providers and users will determine if the intervention is acceptable.

Feasibility: Degree of convenience for use. If 70% of participants contacted consent/assent to participate, are retained in study for 6 months, adhere to the protocol, complete at least 4 out of 6 sessions and assignments, and recruitment happens in one month as planned, then intervention will be considered feasible [9].

Introduction

In sub-Saharan Africa (SSA), where 82% of the global HIV-positive young people live, adolescents are persistently among the most at-risk populations (MARP) for HIV [10,11]. In Uganda which is 10th among SSA countries with the highest HIV prevalence (6.7%), an estimated 1.4 million people are living with HIV/AIDS (PLHIV) [12,13]. The HIV prevalence in Uganda is even higher among young people, who presented about 19% of all new HIV infections in 2015 [12]. With many children born with HIV reaching adolescence in concurrence with the youth bulge in SSA[10], the importance of contextually appropriate HIV care and prevention strategies focused on adolescents living with HIV/AIDS (ALWHA) needs to be emphasized. In Uganda specifically, 34.8% of the population is between 14-24 years of age[14]; the same group also accounts for almost 50% of HIV cases, warranting a focus on this vulnerable group [15].

Despite efforts to improve HIV care among ALWHA in SSA, current interventions have not optimized the intended impact[16]. In Uganda, young people continue to be the only group with increasing HIV-related mortality amidst general declines in other groups [17]. Among the individual, interpersonal, community and health systems challenges, the increase in morbidity has been attributed to poor medication adherence among young ALWHA [18]. Successful antiretroviral therapy (ART) programs are highly dependent on adherence to medication and sustained care engagement; further, slowed progression to AIDS, sustained viral load suppression (VLS), reductions to ART resistance, and lengthened survival rates are achieved through maintenance of a >95% daily oral dosing of ART [19]. Studies using medication possession ratios and clinical counts have found a higher proportion of older people to be more adherent to medical regimens compared to adolescents (72% adherence among adults compared to 68% adherence among young people) [20]. Furthermore, VLS data, a key indicator of medication success among PLHIV, shows that in 2017, only 39.6% of young people had achieved VLS, compared to 74.2% of adults [15].

Barriers to ART adherence among ALWHA include socio-economic (poverty, lack of food, loss of parents, and school environment), psychosocial and individual (perceived and experienced anxiety, fear, discrimination, lack of family support, peer influence, depression, privacy concerns in schools & stigma), and treatment-related factors (drug fatigue, pill burden, lack of clinical support, medication side effects and distance to health facilities)[18] [21-23]. Although a clear categorization of barriers exist, psychosocial factors are often embedded within other barriers [24] and seem predominant among ALWHA since they are exacerbated by the onset of puberty [25].

The unique developmental changes at the onset of puberty play an important role in shaping perceptions, beliefs, attitudes, self-efficacy, evaluation of severity of disease and pathways to care[26]. Biologically, the start of puberty remodels the brains dopaminergic system, resulting in increased risk-taking, emotionality, and egoistic tendencies, making adolescence a time of

heightened vulnerability to risk and reckless behaviors such as taking medication holidays [27]. Adolescence is further complicated by self-regulatory challenges, preference for immediate gratification, exploration, secretiveness and concrete thinking [28]; this period also coincides with changes in the social environment, such as spending less time with parents and more with peers, as well as an increase in autonomy [29]. A combination of these changes affects psychosocial pathways to health[30] by inducing increased entanglement with thoughts (cognitive fusion) and continuous attempts to control thought experiences (experiential avoidance)[31], resulting in psychopathological experiences such as depression, anxiety, and internalized stigma; these factors are documented causes of ART non-adherence among ALWHA [32,33,34]. Since psychosocial challenges impact all aspects of treatment among ALWHA [24], there is a need to promote developmentally appropriate and culturally sensitive psychosocial support interventions (PSI) if treatment efficacy is to be improved in this population. The shortage of such interventions will continue to undermine other structural or health systems-level efforts to improve health outcomes among ALWHA.

Psychosocial support interventions such as; short message services (SMS)[35], cognive behavioral group therapy services[36], individual counselling, individual + group counselling[37], treatment supporters [38], Suubi+ [39] and many others, have been posited to promote ART adherence among PLHIV in Uganda. However, interventions that resonate with the unique needs of adolescence as a developmental stage are scarce [24]. Further, a majority of these previously tested interventions were designed for adult populations[18], and research has shown that adult-focused interventions have limited utility and efficacy among young people[40]. Further, many of the reviewed interventions such as SMS are costly to sustain with adolescents [28], and have been implemented without adaptation[41] and the mechanism of their proposed action and resulting behavior change, remains unclear, limiting replicability and scalability [42].

Evidence-based, contextually appropriate and theory-informed psychosocial interventions focused on targeted pathways that address adolescent's complex demands need to be adapted, developed and tested to improve optimal adherence in resource-limited settings like Uganda [28] [40].

Mindfulness and acceptance-based psychosocial interventions are increasingly gaining traction as appropriate and effective support interventions for young people[43] [44] [45]. These interventions focus on increasing self-regulation of attention and with curiosity, openness and acceptance as a way of dealing with painful thoughts, feelings and conditions. Developmental changes in adolescence are mostly impacted by emotions, yet executive functions such as judgement impulse control and emotion regulation are still underdeveloped. Since mindfulness can regulate emotions, it can counteract this imbalance[46]. Acceptance and Commitment Therapy (ACT), is one particular mindfulness-based intervention that has produced promising results when used with adolescents with chronic conditions[47] [48] [43].

ACT is an intervention that improves mental health through psychological flexibility[42]. Considered as part of the "third wave" Cognitive Behavioral Therapies (CBTs) [49], ACT focuses on context sensitivity and functions of psychological phenomenon [50]. It aims to change the function of events and relationships individuals develop with events using metaphors and experiences that support psychological flexibility[51]. ACT-based interventions have been shown to improve quality of life [52] and mental health among adults LWHIV [53,54] and young people with chronic diseases [55,56,57]. It has also proved to be feasible and acceptable among adults living with HIV/AIDS [58]; however, no study has assessed its relevance and appropriateness for ALWHA.

One particular ACT model that is used with young people is the; Discoverer, Noticer and Advisor-Values model (DNA-v model) [59]. This model integrates three functional classes of skills to promote psychological flexibility, the main tenant of ACT-based therapies. DNA-v is based on the assumption that, unlike adults, young people have limited well-established values to guide behaviours. Rather, they are often discovering and creating or improving values as they journey into adulthood[59]. DNA-v helps young people develop strength, overcome unhelpful thoughts, adjust behaviors without attempting to control thoughts and live fully in the present moment, making choices that help them reach their full potential [60].

ACT-DNA-v has proved effective with adolescents in other settings[61], however, to enhance its potential for inclusion in mainstream HIV care for adolescents in Uganda, there is a need for exploring its acceptance among HCPs and ALWHA, testing its feasibility and preliminary effectiveness among ALWHA, and potential contextual adaptations to fit the experiences, belief systems and assumptions in Uganda

Problem statement

The success of ART is highly dependent on adherence and persistent care engagement. However, despite efforts to improve sustained ART use among ALWHA, non-adherence persists. Studies using medication possession ratios and clinical counts have found adherence to ART among adults to be at 72% compared to 68% among young people[20], furthermore, statistics on VL measures in 2017 show that 74.2% of adults above 50 years had achieved viral load suppression compared to 39.6% of adolescents [15]. Additionally, almost 50% of HIV cases in Uganda are among young people[62]. Poor adherence among ALWHA undermines the HIV care cascade and efforts to end the AIDS epidemic by 2030. As a consequence, young people are the only group in Uganda among whom HIV mortality is increasing. The unique developmental changes at the onset of adolescence have been cited as the main factors influencing psychosocial pathways into health, resulting in psychopathological barriers to medication adherence among ALWHA[24]. Increased risk-taking, heightened vulnerability, emotionality, and egoistic tendencies that come with the onset of adolescence, tend to dominate over other sources of behavioral regulation resulting in cognitive fusion (getting entrapped with unhelpful thoughts)[63]. Cognitively fused ALWHA get attached to internal events and begin to predict fear, develop negative beliefs about pathways

to care and engage in experiential avoidance: try to regulate and avoid their thoughts, feelings and body sessions even when doing so causes more harm. Cognitive fusion and experiential avoidance result in psychopathological outcomes such as; depression, anxiety and stigma which affect adherence to medication [64]. To achieve successful adherence and care engagement among ALWHA, interventions tailored to the dynamic social and cognitive needs of adolescents as they pass through life stages must be tested and promoted.

To date, psychosocial support interventions targeted for the unique developmental changes in adolescence remain limited. Most interventions in use with young people are developed for adult populations, despite adult-focused interventions being shown to have limited effectiveness among young people[40]. While ACT-DNA-v has successfully helped young people manage cognitive fusion and experiential avoidance effectively through proper management of emotions, connecting with values, and utilizing mindfulness to build health relationships (factors that are proximal to medication adherence), it has yet to be culturally adapted and evaluated for use among ALWHV in Uganda. The disparities in HIV transmission and HIV-related mortality among young people underscore the need for evidence-based psychosocial interventions to increase ART adherence and subsequent health outcomes among this group.

Justification

The National Antiretroviral Treatment Guidelines for Uganda (2020), require all young people initiated or continuing ART to be provided psychosocial support services which are developmentally appropriate, acceptable, visible, flexible, affordable, contextually appropriate and universally available to improve medication adherence. Most of the available psychosocial support services offered to young people such as short messaging services, treatment supporters, Suubi+, adherence counselling and others, were originally developed for adult populations and not specifically based on the unique developmental needs of ALWHA[65]. Further, the mechanisms of change used by these interventions remain unclear, making it hard to replicate or scale up these interventions and some are costly to sustain in resource-limited settings.

Mindfulness and acceptance-based interventions, specifically ACT-DNA-v, are developmentally appropriate for adolescents because they are designed to promote emotion regulation and values consistent living, counteracting the imbalance created by developmental changes amidst undeveloped executive functions such as judgment[66]. ACT-DNA-v also fosters the development of healthy relationships, which are critical for medications and treatment, and have been shown to facilitate self-efficacy and medication adherence. Further, adaptations based on psychological, process and social developmental factors support the appropriateness of ACT-DNA-v for adolescents[67]. Psychologically, symbolic reasoning develops around age eleven and DNA-v relies on art and metaphors which are aids to symbolic reasoning[55]. Secondly, DNA-v is built on assumption that, unlike adults, young people have limited well-established values to

guide behaviors yet they are struggling with identity at the same time. Rather than fully focusing on value classification, DNA-v combines both values formulation and clarification because young people are often discovering and creating their values as they journey into adulthood[59]. Finally, the ability to have group-based ACT therapy has the potential to address various dimensions of peer influence, which is known for driving both positive and negative behaviours among adolescents[55]. Studies conducted in the developed world have demonstrated that mindfulness and acceptance-based interventions are promising for use with adolescents across a variety of health topics [60 68 69 70]. The fact that ACT-DNA-v is developmentally appropriate for adolescents, justifies adapting the intervention for the Ugandan context, exploring its acceptance among users (ALWHA) and providers (HCPs), and measuring its feasibility and effectiveness in improving ART adherence among ALWHA for potential inclusion in care settings. Additionally, ACT-DNA-v can be delivered by lay providers/support staff, following proper supervision, and is potentially scalable in resource-limited settings. Upon effective evaluation, the intervention will be used by HCPs (counsellors) during one counselling session or in group therapy with adolescents as part of clinical support.

Theoretical Framework

The underlying theories linking ACT-DNA-v to ART adherence are the Health Belief Model (HBM)[71] and the Relational Frame Theory (RFT) [49]. According to the HBM, modifying factors such as demographic characteristics and their accompanying psychological states indirectly affect sick role behaviors and clinic use by influencing threat perception and behavioral evaluation[72]. Threat perception involves the anticipated severity of the consequences of illness, while behavioral evaluation involves beliefs concerning benefits and efficacy plus costs and barriers to enacting sick role behavior. Thus, the perceived benefits of preventive behavior and the potential costs of neglecting such a behavior are the key beliefs that shape health-related behavioral outcomes such as medication adherence[72].

RFT relays that threat perception and behavioral evaluation are made possible by language function and precede cognitive entanglements[31]. The development and use of language to communicate create contextual relations among events[63], making humans experience events even in their absence[49]. These derived relations tend to dominate over other sources of behavioral regulation, making humans experience thoughts in form of change they produce rather than as processes occurring in the moment, resulting into cognitive fusion (getting entangled with thoughts). Cognitively fused individual attach temporal and evaluative relations to internal events, begin to predict fear, try to regulate or avoid their own thoughts, feelings and body sessions even when doing so causes more harm (experiential avoidance). Such individuals further develop and maintain painful self-descriptions and treat such descriptions as right, resulting into psychopathological outcomes such as stress, depression and anxiety[73] which are proximal to poor adherence[30]. ACT targets cognitive fusion, experiential avoidance by modifying

the relationship an individual form with thoughts by skillfully and mindfully normalizing experiences in an open, curious and elaborate way that promotes values consistent behaviors such as adhering to medication even in presence of difficult thoughts and experiences[1].

Conceptual Model

Figure 1 shows a conceptual model of the hypothesized relationships between barriers to ART uptake among adolescents and psychosocial pathways to health (enhanced by ACT intervention) and ART adherence/viral suppression.

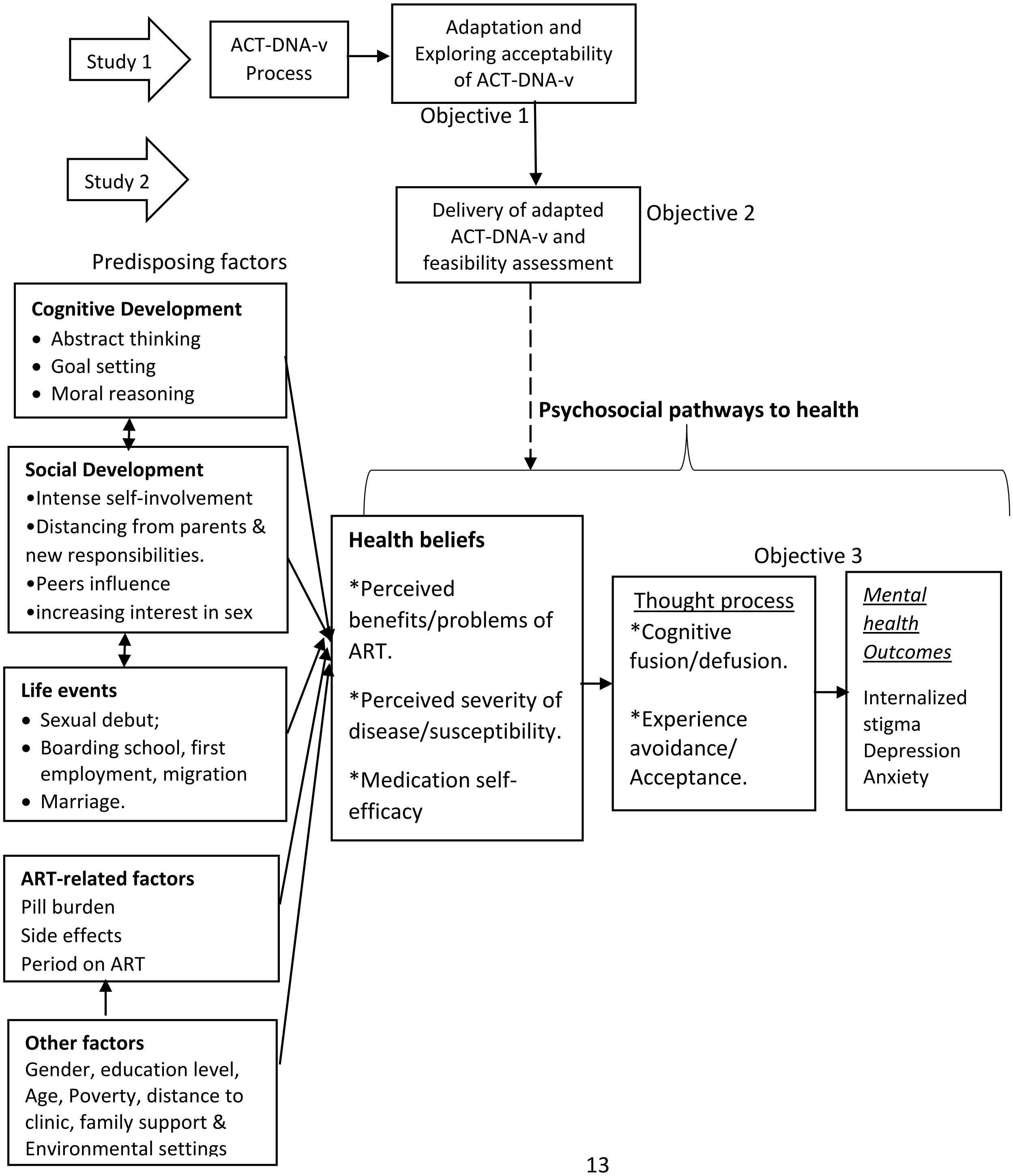

Concepts drawn from Sawyer et al.[74], the Health Belief Model [75] and the Relational Frame Theory [76].

Research Questions

1. How acceptable is the adapted ACT-DNA-v among providers (HCPs) and users (ALWHA)?
2. How feasible is the adapted ACT-DNA-v intervention for use with ALWHA?
3. What is the impact of the adapted ACT-DNA-v on reducing depression, anxiety and stigma as psychosocial barriers to ART adherence among ALWHA?

Purpose Statement

The purpose of this sequential multi-methods study is to explore the acceptability of a mindfulness-based psychosocial intervention among providers (HCPs) and users (ALWHA) and measure its feasibility and preliminary effectiveness in improving the mental health of ALWHA.

Research Objectives

1. To adapt and explore the acceptability of ACT-DNA-v among users (ALWHA) and providers (HCPs).
2. To measure the feasibility of the adapted ACT-DNA-v for use with ALWHA.
3. To examine the impact of ACT-DNA-v on reducing proximal psychosocial/mental health barriers to medication adherence (depression, anxiety and stigma) among ALWHA.

Hypothesis.

(i) Participants receiving the ACT-DNA-v intervention will report a significant reduction in depression, anxiety & stigma at post-intervention assessment as compared to participants in the control group.

Study period

The study will run for nine (9) months. The first two months will involve the adaptation of ACT-DNA-v and exploring acceptability among users and providers, this will be followed by organizing and conducting a randomized control trial lasting for three.

Literature review

Introduction

In the section, a review of literature and arguments around barriers to ART adherence among adolescents, possible interventions used in Uganda and the appropriateness of a mindfulness-based intervention for adolescents is done. The section is organized into different sub-themes, starting with an overview of HIV among adolescents, the role of ART in the treatment of HIV, adherence to ART among adolescents, factors associated with sub-optimal adherence, available interventions supporting adherence among adolescents, and rationale/justification for using mindfulness-based intervention with adolescents having chronic conditions.

HIV among adolescents

Globally, over 1,700,000 adolescents aged 10-19 years are estimated to be living with HIV/AIDS, and account for 5% of the world's total number of people living with HIV [77]. With close to 170,000 new infections among adolescents worldwide and an average of 34,000 HIV-related deaths each year, HIV is still a big challenge among young people [77]. The burden is even worse in Sub-Saharan Africa (SSA) where an estimated 1,500,000 HIV-positive adolescents live (88% of the world's total). In Uganda, one of the countries in the SSA region, 260,000 young people are estimated to be living with HIV: 160,000 aged between 15-24 years and 100,000 aged between 0-14 years respectively[78], accounting for approximately 18.5% of the estimated national total of people living with HIV/AIDS[79]. The national HIV prevalence among young people stands at 2.1%, with higher proportions in girls compared to boys[79]. Adolescents acquire HIV either vertically (prenatally) from their HIV-positive mothers who could have missed diagnosis as a result of loss to follow-up or improper mother-to-child transmission programs and horizontally; either through early sexual intercourse, cross-generational sex and female sex gender-based violence or non-sexual means [80] [77].

The general improvement in HIV treatment and care has seen many children born with HIV reach adolescence-which is a stage of heightened activation, necessitating more intensified adolescent-focused intervention[78]. The national effort to enrol HIV-positive adolescents on medication as a way of reducing deaths and transmission has seen over 92.5% of adolescents diagnosed with HIV get initiated on ART[79], however, out of those enrolled, only 65.5% report adherence and just 54.8% achieve viral suppression[79]. The percentage is below the UNAIDS ambitious goal of 95-9-95 as a way of ending HIV by 2030, calling for increased understanding of adolescence as a stage and developing support interventions that are developmentally appropriate[81].

In planning care for adolescents, the efficacy of clinical medicine must be calibrated against the background of accelerated physical, psychological and developmental changes since they influence uptake of services, pattern of diseases and maintenance of relationships with providers[82]. Notable changes include: "achievement of biological and sexual maturation, development of personal identity, development of intimate sexual relationships, establishment of independence, puberty and completion of growth, developing of a sexually attractive body, abstract thinking, having a clearer sense of sexual and personal identity, emotional independence, strong peer identification and a craving for autonomy"[82]. Dealing with such changes most likely make adolescents vulnerable to psychological and behavioral problems like

emotional regulation, relationship challenges, inability to concentrate, experiences of anxiety and identity formation[73]. It is evident that even without a disease condition, adolescence is already is a stressful stage involving dealing with many vulnerabilities and undertaking risks that have lifelong consequences for health and well-being [83].

Adolescents living with HIV suffer a double burden of keeping on daily medication while at the same time negotiating life changes. Thus, support initiatives for this group should be given having this burden in mind "if issues of adherence, identity, consent and confidentiality are to be managed"[82].

Role of ART in the Treatment of HIV

Research on the treatment of HIV has advanced[84]. The first therapy to work against HIV was the nucleoside reverse transcriptase inhibitor zidovudine in the early 90s, however, single-dose treatments were later observed not to be effective in slowing the progression of the virus and also resulted in mutation of the virus [84]. Advances in research suggested that combining medications could come with added advantages and better impact [84]. This resulted in the creation of a combination regimen termed Antiretroviral therapy (ART)[85]. This is a daily treatment of multiple HIV medications, with a typical ART regimen usually comprising a "backbone" of 2 nucleoside reverse transcriptase inhibitors (NRTIs) and a 3rd "Anchor" ARV from another class[62]. "Although not curative, ART provides longer lives for patients and reduces transmission of HIV"[84].

HIV attacks and destroys disease-fighting cells of the body called CD4 cells[85]. The reduction in disease-fighting cells compromises body immunity, allows HIV to make copies and opens up the door to all infections that make the body weak and susceptible[77]. A combination of medicines stops the virus from making copies of itself, lowering the amount of virus in the body while at the same time allowing the body a chance to recover and produce more CD4 cells, hence improving body immunity[80]. The overall aim of HIV treatment is to reduce viral load to undetectable levels. People with undetectable viral loads, live healthier, do not transmit the virus to others and develop better immunity[80]. Following the benefits arising out of ART, the consolidated guidelines for the prevention and treatment of HIV in Uganda, have recommended that all people diagnosed with HIV should immediately be enrolled on ART as a way of reducing HIV-related deaths and transmissions [81].

Adherence to ART among adolescents

The success of ART depends on proper adherence by maintaining a daily dosing and receiving 95% or more of prescribed doses in a given period[19 85]. Adherence "is the extent to which a person's behavior (taking medication, following diet and or executing life style changes) corresponds with agreed recommendations from a health care provider"[5]. Adherence extends to filling prescriptions, taking medication appropriately, attending follow-up appointments and executing behavioral modifications[5]. Successful adherence results in viral suppression which is key in reducing HIV related mortality, morbidity and transmission[81].

Even though the benefits of ART in treatment of HIV are numerous and known [86], adherence is still a challenge in the general population and worse among adolescents [87]. Compared to children under age 10 and adults above 20 years, adolescents still stand out as a group that is less likely

to achieve viral suppression (an outcome of adherence to ART) [88]. Much as young people are more likely to `seek' and `test', they are less likely to engage and retain in HIV care [89]. Evidence reporting low levels of adherence among adolescents is vast [89]. In an earlier study on self-reported adherence to ART among adolescents, only 28.3% of participants reported having followed prescriptions in the previous month, with the majority skipping a dose once or twice[26].

According to the Uganda population-based HIV impact assessment (UPHIA-2017), viral load suppression (VLS) is generally lower among adolescents compared to adults [79]. VLS is reported at 49% for older adolescents (14-19 years), 59% for adults (30-34 years), 67% for adults (40-44 years) and 74% for adults (45-54 years)[79]. In a related systematic review and meta-analysis on adherence to ART across Africa, overall, adolescents were less adherent (68%) compared to adults (72%)[90]. A similar result was also reported in a Rwanda study where adherence among adolescents was reported at 45% [91]. However, contradicting results reporting better adherence among young people have also been identified. In a study carried out in 10 Ugandan districts, 90% of adolescents on ART achieved over 95% adherence rating, with adolescents in urban districts reporting better adherence than adolescents in rural areas[92]. Similarly, in a cross-sectional study on factors associated with adherence to ART in Kabale district, 79% of adolescents in the study reported being adherent [93]. Although varying levels of adherence have been identified [93,94] [95], concession remains that adherence to ART is still suboptimal in young people compared to adults.

Factors associated with adherence to ART among adolescents.

To achieve the UNAIDS 95-95-95 target and end HIV in the world by 2030, adherence to ART among adolescents must be encouraged and supported. Since adolescence is a unique developmental stage with spontaneous physical, psychological and social changes[82], the interplay of such factors should be understood if adolescent-focused interventions are to be promoted.

The World Health Organization (WHO) created 5 dimensions of adherence which are ideal guides in identifying and classifying factors associated with adherence to ART across the general population [85]. Much as these dimensions are not adolescent-specific, they can still provide a framework and a starting point for classifying adolescence-centered factors. The WHO dimensions suggest looking at adherence as systemic to have a comprehensive understanding of interconnections. The 5 dimensions include socio-economic, health care system-related factors, clinical condition related, therapy-related and patient-related [85].

Socio-economic factors related to adherence to ART among adolescents

ALWHA have been identified as experiencing a double burden of dealing with developmental changes as they transit into adulthood, while at the same time maintaining daily medication [96]. Adolescence as a developmental stage is comprised of physical, pubertal, cognitive and psychosocial changes such as: identity formation, independence, and abstract reasoning [37]. These changes also coincide with social re-arrangements such as moving from home to school, or starting life on your own. These new realities alternate responsibilities and result in situations where adolescents are expected to take charge of their own health and not solely depend on care givers[37]. Even with increased independence, the transition from concrete to abstract

reasoning makes adolescence a higher-risk stage. Abstract reasoning encourages believing in what one has experienced not what is logical, thus, behaviors that serve immediate gratification be prioritized and whatever has a long time gain can be delayed. Taking ART does not yield such immediate observable results and thus, may not be treated as priority [37]. Besides, irrationality results in generating quicker ways to deal with challenges such as avoiding medication to put the burden aside [91], yet adolescents still have undeveloped risk control skills and impulse control skills[37]. Relatedly, adolescence is also an identity formation stage. ALWHA find it challenging to incorporate a chronic condition in their identity formation. As a way of sculpting an ideal identity, adolescents opt to detach from treatment and care to feel normal like others [91].

Developmental changes aside, social relations and support are also pivotal in influencing adherence among adolescents. These are relationships with peers, parents or other members of the community. Peer evaluation and acceptance are often prioritized and adolescents will gladly adjust life to fit in friendship circles [97]. Young people are drawn more to their peers and are consumed with the desire to fit in and be accepted so may be embarrassed to take their medication in the presence of peers [97]. Besides, young people may avoid medication to protect romantic relationships or to please their partners [88]. Most of these factors around peer evaluation and appraisal are related to perceived or experienced stigma [91], many young people would rather skip doses, pause treatment or hide their status out of fear of losing friends or getting segregated due to status[91]. Furthermore, support from caretakers or parents also promotes adherence among adolescents[98]. Young people with higher adherence have a better acceptance of diagnosis and treatment because they are less likely to experience discrimination and isolation at home, signifying that caregiver/parental attitude and experience are pivotal in influencing adherence [98]. Caregivers' sense of obligation and commitment in the form of sacrificing time to care, signing up and attending programs, giving resources and sometimes sacrificing their food for the adolescent is important to support [99]. Caregivers who are also living with HIV have been found to offer more support to ALWHA[93].

Related to social relations; concerns about privacy and changes in the physical environment are interrelated factors that have widely been identified to influence adherence among adolescents [91]. In one study, 19 out of 42 participants reported to be living in foster homes or orphanages and 30 out of 42 were living in boarding schools. Foster homes and boarding schools lacked privacy to enable adolescents to take medication without experiencing stigma. Many were reported to have abandoned medication for fear of being seen[91]. Foster homes are congested households that offer little privacy and besides, caretakers in such households do not offer appropriate social support they would otherwise have received from their biological parents [91]. Privacy is further complicated by difficulties in packing medicines safely to school, changes in routines; like during examination times when adolescents cannot leave the exam room to take medication, and being on the move from one relative's home to another or one foster home to another, getting exposed to new people all the time [100].

Another factor related to privacy is fear of loss of confidentiality. Situations that include the disclosure of sero-status deter adolescents from continuing with treatment. Many would rather not go to a community clinic for fear of being seen in the line or opening up about their status to teachers at school [92]. This quest for privacy denies adolescents opportunities for social support.

Furthermore, breach of confidentiality by health workers or exposing patient's medical records is another concern to ALWHA and most times this leads to not keeping medical appointments, going for refills or engaging in care [89].

The social construction of HIV also affects adherence among ALWHA. Society still identifies HIV as a death sentence and categorizes people living with HIV as social misfits. In a conceptual model derived from a study on community beliefs, stigma and depression among adolescents in rural Uganda, "community perceptions about presumed early mortality, predisposed ALWHA to experience enactment and internalization of stigma that led to depression and ART non-adherence"[101]. Community members and peers especially in schools intentionally discriminate ALWHAs. They are often given sorts of names that depict them as risks to others and social misfits [101]. The pain is worsened by experiences of discrimination from their parents who openly prioritize other children at the expense of ALWHA since they are seen as a wasted investment without a future. This continued witch-hunt and externalization results in internalized feelings of shame and guilt, lowering the quality of mental health and altering health-seeking behaviours. Adolescents abandon medication because they do not see the relevance of remaining alive when they cannot be accepted by society [101]. The model identifies psychosocial support and early disclosure of status to be protective factors [101].

Poverty and the related logistical challenges also influence adherence among adolescents in resource-constrained settings[99] . Since young people do not have a source of income, the burden is often experienced by caregivers who report transportation costs to and from clinics and pharmacies to be a big problem. This is even worsened when the treatment plan requires to make frequent visits [102]. Sometimes supplemental medicines have to be skipped because caregivers cannot afford to get them [99]. Besides transport, poverty also compromises the ability to afford food daily yet medicines must be taken after meals [93] [103]. The situation is even worse for youths who come from child-headed families where they are not only expected to provide for themselves but also for their siblings. The desire to attend to family first comes at the expense of giving up on yourself and thus end up missing medication [103].

Health care-related factors

Related to social factors, conditions and experiences at health centres also affect adherence to medication among adolescents. Distance to the health facilities has frequently been identified as either a facilitator or a common barrier to staying in care and making medication refills[93]. This challenge is common with adolescents living in rural areas where health centres are few which requires walking long distances or spending a lot of money on transport to and from the health centre to pick pills[104]. Relatedly, the long lines at clinics sometimes discourage adolescents from turning up for refills. Adolescents fear being seen lining up for medication besides the discomfort of having to wait for long[102]. Furthermore, the conduct of health care workers also impacts uptake of care services and success of adherence interventions [104]. Health care workers are the door way to care, their opinion on adherence, quality of interaction and recommendations play a vital role in encouraging adherence among adolescents. Health care workers need to be mindful of the approach they use with adolescents. Most times one way communication of prescribing may not encourage adherence, but rather, engaging adolescents in open discussions about their medicines results in better adherence [97]. Creation of a good client provider relationship is very

crucial [89]. Such relationships facilitate open communication and informative discussions which invoke trust and honesty, encouraging loyalty to treatment [89].

In a study conducted in Brazil, the establishment of good interactions with healthcare staff and caregivers' trust regarding the benefits of proper treatment adherence was a key element of optimal adherence [98]. This was however in stark contrast to families of non-adherent patients who had limited relationships with healthcare professionals and usually mistrusted the potential benefits of treatments. This mistrust and distancing from health professionals originated mostly from experiences of AIDS/HIV stigma and poor communication which further worsened non-adherence [98].

Therapy (ART) related factors

Factors associated with taking ART also influence adherence among adolescents. ART side effects such as drug toxicity, abnormal physical changes and related psychological reactions are still salient barriers to adherence among adolescents [86]. ALWHA who feel drugged, and nauseated after taking ART sometimes avoid taking medication again[86]. Medication side effects exhaust morale to keep on drugs, while better effects improve confidence to keep on drugs[105]. In a Peruvian study, more than half of the participants reported having suffered side effects after taking ART regimens [106]. The most commonly reported effects were: heartburn/stomach pain and skin rash [106]. In addition to physical effects, psychological side effects such as shame, embarrassment and perception of the drug as a reminder of HIV status are also prominent among ALWHA and hinder proper adherence [26]. Adolescents perceive taking ART as a reminder of their "abnormality" and therefore skip doses to feel "normal" like others [91]. Related to ART side effects is pill burden. Medications are many and burdensome to swallow daily [87] [91], this often leads to treatment fatigue [107], boredom [88] and "drug holidays" [103]. Drugs are also reported to be difficult to swallow [91]. However, with the new improved ART regimens which are simpler and tolerant, experiences of pill burden and drug toxicity have reduced over time[94].

Lack of information about how ART should be taken, the relevance of keeping on medication and what constitutes appropriate dose also hinders adolescents from complying with the regimen[88]. Young people who are misinformed or uninformed about HIV and ART are more likely not to adhere to treatment [88], similarly, people living with HIV who have little or no continuous ART adherence education have less than optimum adherence levels [107], while those who are satisfied with the information given by health workers report higher adherence [89]. Relatedly, being on ART for so long makes adolescents skip doses out of exhaustion.

Clinical conditions-related factors

Clinical outcomes associated with taking ART also influence adherence among adolescents. Notable of these outcomes include: achieving viral suppression, feeling well after taking medication and reduction in HIV related symptoms[108]. Adolescents that receive feedback on their viral load that indicates suppression are more likely to keep on medication [109], also when ALWHA observe noticeable changes in health outcomes such as feeling well, increasing body weight, and improved immunity, they get motivated to keep on medication [108]. Relatedly, is the history of declining health due to suboptimal adherence, many adolescents would opt to keep on medication to avoid a similar circumstance that befell them when they attempted to skip doses in the past. This negative reinforcement gives ALWHAs a reason to take their drugs[88].

Patient-related and psychosocial factors

Since adolescence is a period of transition from being a child to assuming adult identity, most often the responsibility of care is moved from caretakers to ALWHAs. This is the point when individual-related factors mostly become prominent in influencing health-seeking behaviors[97]. The interaction of ALWHs with the social environment impacts their psychological and physical states which might also influence adherence-related behaviors[73]. This assertion takes into consideration that "the mind is the doorway through which individuals experience environmental events"[30]. Thus, social factors such as losing parents, disclosure of status, victimization and discrimination coupled with effects of developmental changes such as attenuated risk-taking, abstract reasoning and desire for immediate gratification[37], affect cognition and also evoke psychopathological experiences such as stress, anxiety, depression, and stigma which are proximal barriers to medication adherence [88].

Experiencing depression is very common among ALWHA [110], and it's always associated with mortalities. According to WHO, 'depression is a mental disorder characterized by persistent sadness and a lack of interest or pleasure in previously rewarding or enjoyable activities. It can disturb sleep, loss of appetite and cause tiredness and poor concentration. Depression is caused by the interplay of social, psychological and biological factors. Higher levels of depression have been identified among ALWHA in Uganda; in a cross-sectional study conducted in Western Uganda, the prevalence of depressive symptoms among adolescents (10-19) was high (46%) and highest among older adolescents (62.3%) [110]. The most reported depressive symptoms are: "feeling unhappy", "feeling like crying" and "lack of appetite"[110]. Similarly, another study carried out in rural Uganda found higher levels of depression among ALWHA. Participants reported experiencing greater psychological pain due to resentment and feeling sad, this often results in self-isolation and sometimes the development of suicidal thoughts like 'taking rat poison', 'throwing self in the lake' and 'cutting the neck'[101]. Evidence of experiences of depression among ALWHA is enormous[85, 111, 98, 88].

Depression is often associated with poor adherence to ART among ALWHA[112]. Numerous studies have reported a negative relationship between depression and adherence to ART[5 106 91]: In a longitudinal multivariable study on adherence among young people in Uganda, depression was the only factor associated with poor ART adherence[113]. In this study, ALWHA's coming from families that had a better support network which was crucial in normalizing experience of depression, reported better adherence than those that did not have [113]. However, a systematic review synthesizing evidence of longitudinal associations between psychosocial predictors and Chronic Preventive Maintenance Medication generated contradicting findings. In this review, depression was identified as a significant predictor of adherence [114]. The authors support their contradicting findings based on the argument that most studies done on depression are cross-sectional and thus focus on momentary experiences, yet if depression is studied longitudinally varied experiences can be obtained[114]. The authors however contradict themselves when they acknowledge that some of the studies they reviewed had bias in study sample, were poorly designed and some used non-validated questionnaires [114], thus, the negative effect of depression on adherence among ALWHAs cannot be underestimated.

Another personal factor influencing adherence among ALWHA is anxiety[115]. Anxiety is a psychiatric condition which is often provoked by worries and fear of adverse HIV-related consequences including death[115], and at one point in the life of a person living with HIV/AIDS, experiences of anxiety occur and most likely alongside other related psychiatric disorders [116]. In Uganda, anxiety is usually reported in around 70% of AIDS patients and it is always associated with sub-optimal adherence to ART[116]. Panic-related anxiety manifesting symptoms such as; difficulty in breathing, fear of dying and heart palpitation relates more to poor adherence than other forms of anxiety [117]. In a Ugandan study, young people with higher levels of panic-related anxiety were 0.71 times less likely to be 90% adherent compared to those who reported low levels of panic anxiety [117]. Furthermore, people experiencing anxiety most likely opt for avoidant coping mechanisms such as denials as a way of normalizing feelings[117]. Avoidant mechanisms are associated with poor health responses and outcomes[117]. In a study on adherence among children and adolescents in Brazil, higher scores on self-reported adherence came from participants who were experiencing mild anxiety (19%) based on a standard scale, while poor adherence scores came from participants who reported experiencing moderate to severe anxiety (average of 44%)[98].

Surprisingly, in a UK study, adolescents reported that living with HIV was not a condition that evoked experiences of anxiety [118], rather, social situations triggered anxiety regardless of status [118]. The study further identified factors such as moving from one caretaker to another, low self-esteem, pre-occupation about life not being worth living and lower social function to be proximal to anxiety and poor adherence among ALWHA [118]. It is however important to observe that the prevalence of mental health often differs across populations, and it would be premature to conclude one study conducted in a resource-rich setting since the lived experiences of ALHWA's in such settings differ from those in low-resource settings.

Experience of stigma is also associated with poor adherence among ALWHA[91]. Goffman conceptualizes stigma as 'an attribute that is deeply discrediting and seen to confirm the uselessness of another person and is neither credible nor dis-credible as a thing in its self'[119]. Society crafts identity that is accepted or rejected and people often make anticipations of others based on societal normative expectations, resulting in distinctions of what is normal from abnormal, desirable from undesirable, and narrowing others in simplified lenses that are tainted or discounted[119]. Stigma among ALWHA is experienced in all corners of social life but most profoundly from homes and schools, which ideally should be protective environments [120]. Stigma can be enacted (experiencing unfair treatment from others), anticipated (belief that prejudice and discrimination will be directed to you) or internalized (absorbing and believing negative messages)[121]. Kimera identified five themes that represent stigma among adolescents; 'being devalued, experiencing fear, experiencing injustice, feeling lonely and lack of future perspectives' [121] and these themes were found to have negative effects on the uptake of HIV care services including adhering to medication. A stigmatized person will always try to avoid experiences or encounters that attenuate it [92], and most times ALWHA's experiencing stigma abandon care [92 122 101 123 97].

Related to stigma is stress. In a Ugandan study on the relationship between psychological distress and adherence to anti-retroviral therapy among adolescents, the impact of stress on ART was

emphasized[124]. These findings are consistent with other research on psychological distress and non-adherence to ARVs[65].

Adherence self-efficacy, goal setting and perception of one's health are other personal factors that influence adherence to medication among ALWHA. Adherence self-efficacy defined as 'confidence in one's ability to adhere to treatment plans'[95] can influence behavioral modification, setting adherence-related goals and changing the way adolescents perceive their lives[95], yet such positive attempts facilitate adherence [88]. Nabunya studied adherence self-efficacy and adherence among adolescents in southwestern Uganda and found that ALWHA's who showed positive adherence efficacy reported better adherence to ART[88], similarly, in a South African study, adolescent girls who participated in a peer support club reported having gained increased self-efficacy which was pivotal in influencing adherence[125]. Relatedly, ALWHA's who live in unstable families with compromised support or who face discrimination because of being the only children living with HIV in the home, experience 'insecure attachment' affecting their self-efficacy and image which are crucial in building resilience that is key to adhering to long term treatments[123]. Regarding the perception of one's health, ALWHA's who view themselves as healthy and serving a purpose in life, tend to follow medication prescriptions more than those who perceive themselves as unhealthy [115]. Also perception towards the ineffectiveness of ART or negative outcome expectations that ART will make one sicker, have an inverse relationship with adherence to ART [88]. These perceptions mainly develop due to lack of proper information and fear of side effects[88 107 89].

While adherence to treatment is an interplay of situations, personal related factors triggered by psychosocial antecedents, are predominant among adolescents and should be given much consideration when designing or recommending interventions [126]

Interventions to support adherence to ART among adolescents.

Several interventions supporting adherence to ART among adolescents have been developed and tested, with varying levels of effectiveness, sustainability and appropriateness. Notable of these include; Education, counselling, Adherence clubs, short message services, electronic dose monitoring, health care delivery restructuring, economic incentives, and food security incentives [127]. Additional interventions such as standardized counseling through multi-media technology, and decentralized and differentiated models of care have also been suggested [127]. These interventions target different barriers from physical, economic, and social to psychological. Since psychosocial-related challenge tend to have much influence over adolescent's behaviours, a review of available psychosocial interventions used with adolescents in Uganda is appropriate.

Psychosocial interventions

When it comes to explaining well-being in long-term conditions such as HIV, It is more than just the diseases and their functions[128]. Living with HIV involves many daunting self-management behaviors like adhering to daily medications, attending appointments, and amending activities/diets which affect the quality of life [128]. These self-management behaviors can be hard for adolescents who have psychological distress as a result of their diagnosis. Fortunately, there have been interventions that have been developed to address psychological issues in individuals with long-term conditions. These interventions can improve adherence, Quality of Life (QoL) and

disease self-management.[128] In a Ugandan study on the relationship between psychological distress and adherence to anti-retroviral therapy among adolescents, the role of therapy was emphasized in the management of chronic conditions[124].

While evidence supporting the efficacy of psychosocial support interventions for promoting adherence among adults is enormous, a dearth of similar evidence among adolescents still exists [126]. Most of the available psychosocial interventions are originally and purposely designed for adults, even when they are being used with adolescents with less cognizance of their uniqueness[126]. 'Psychosocial support interventions are interpersonal/informative activities, strategies or techniques that target biological, behavioural, cognitive, emotional, interpersonal, social and environmental factors to improve an individual's health functioning and mental wellbeing'[126]. Some of the most notable psychosocial interventions used with ALWHA in Uganda include:

Suubi +: This is a psychosocial program that was developed following the assets theory. The underlying message is the economic empowerment of adolescents as a means of reducing experiences of depression and anxiety which are proximal to poor adherence to ART[129]. The intervention was premised on the logic that ALWHA's without material resources find it difficult to access necessities of life such as food which hinders uptake of ART. Suubi+ was further constructed to promote higher levels of self-efficacy and increase the degree of hopefulness about the future[129]. While economic empowerment is very promising in improving HIV care uptake, the sustainability of such interventions in resource-restrained settings beyond the study is questioned. Besides, Suubi is hinged on the assumption that adolescents can easily make rational business decisions, and investment choices, however, possessing such a high level of prioritization or conceptualization is questionable [82] .

Relatedly, the provision of health and psychosocial education also improves adherence to medication among adolescents[130]. Information is usually provided through training and workshops that address concerns such as; how to live with a positive diagnosis, HIV treatment, navigating stigma and the benefits of proper dosing. The overall aim is to use information to influence choices and health-seeking behaviors[126]. Much as having adequate knowledge about medication regimens are among the most prominent factors influencing adherence among adolescents living with chronic illnesses, multifaceted interventions that combine information sharing and other factors or skills tend to be more effective [130]. Besides, knowing alone may not be sufficient to drive action or cause a change of behaviors[108]. Support interventions that provide information, create knowledge, impart skills, encourage goal setting and involve experiential exercises can cause immediate impact[2].

Short message services (SMS) have also been tested with young people in resource-limited settings as cues to action that promote medication adherence. These are reminder messages on antiretroviral adherence[35]. adolescents find SMS to be effective reminders of when to take medicine and a great source of social support[131]. However, 'despite existing evidence supporting the effectiveness of SMS in promoting adherence among young people', some studies have not found SMS to be effective in achieving behavior change in this population [35]. Concerns of confidentiality have always characterized the use of SMS, with many worries around unintended disclosure. Besides, mobile phone reach and costs associated with usage still limit their uptake

among young people in resource-limited settings[131]. Interventions that are cost-effective, more private and developmentally appropriate for young people need to be prioritized.

Group counselling using Cognitive Behavioral Therapy (CBT): This intervention is based on the assumption that 'problematic, cognitions and emotions' are nurtured through learning and thus, the same path can be used to 'modify' such behaviors when an individual is exposed to new knowledge and skills [132]. CBT enables reflection on own processes and the development of new 'perspectives' utilizing social, emotional and behavioral skills to improve wellbeing[132]. Most efficacy trials on CBT-guided counselling in Uganda have been done with adults[133], however, Senyonyi conducted a randomized trial to examine the effectiveness of 'Teens linked to care' (a psychosocial support intervention that uses CBT) on reducing depression, anxiety and alcohol use- (which are proximal to poor adherence) among prenatally infected adolescents attending care at Mild May clinic. Both depression and alcohol use showed no significant difference between groups post-intervention, although the experimental group reported lower levels of anxiety [134]. This study prompts a look into the effectiveness of CBT for adolescents.

The success of CBT in improving mental health in the general population cannot be underestimated, however, appropriateness for adolescents and CBT's mechanism of change needs further clarification. Traditional CBT creates improvement through aiming at 'changing, disputing and restructuring content of thoughts', this however can result in a paradoxical reoccurrence of the same thought or avoiding the experience[4]. Adding a component that focuses on improving the relationship between the thought and the person can help address experiential avoidance and this is what 'third wave behavioral therapies do'[4]. Hayes (2004) writes that one of the biggest failures of first-wave cognitive therapies, like CBT, is that they fail to deal directly with cognition[31]. Mindfulness-based therapies that focus on changing the context in which thoughts occur rather than the content of the thoughts can help address weaknesses with CBT's [135].

Mindfulness and acceptance-based support interventions for adolescents

Bishop (2004) defines mindfulness in two-fold, first 'as a process of self-regulating attention that is maintained by immediate experience and allows increased recognition of mental events in the present moment. Secondly, as a particular orientation characterized by curiosity, openness and acceptance'[7]. Fortuna et al. also define mindfulness as "maintaining a moment-by-moment awareness of thoughts, feelings, bodily sensations, and surrounding environment and can help support specific behaviors, experiences, and psychological processes"[136]. Mindfulness and acceptance-based interventions have generated positive clinical outcomes among adolescents with chronic conditions [137] [138], with acceptance and commitment therapy standing out as the most prominent[139].

What is Acceptance and Commitment Therapy (ACT)

ACT is 'a third-wave behavioral therapy' that combines mindfulness, acceptance, values clarification and behavioral goal setting[140, 141]. It focuses on influence of language on behavior and is informed by a relational frame theory (RFT)[128, 142].

RFT is a modern behavioral theory on language and notes that psychological suffering is based on the way language works[143]. The way we think about our experiences influences how we handle

or perceive what is going on. They note down the FEAR acronym from RFT-based ACT, which stands for Fusion, Evaluation, Avoidance and Reason-giving. Cognitive fusion is when we allow our thoughts to influence our perception of a situation, instead of basing it on empirical evidence. This fusion then leads to experiential avoidance. Evaluation is the way we look at our internal states, and practices like comparing ideals to present reality. This involves negative self-evaluation which leads to suffering. Avoidance is actively avoiding or suppressing unpleasant experiences or thoughts. Something that is known to only lead to these unpleasant thoughts dominating one's life. The reason giving is when one allows reasoning through language, such as "I am worthless", to shape their life. These reasons usually come from experiences one may have had, nonetheless, this type of reasoning based on one's experience is usually untrue[143].

ACT uses metaphors and mindfulness exercises to transcend the limitations of words it has been compared to Buddhism since they both aim at finding inner peace by taking more control of thoughts, emotions and behavior[4]. ACT sets out to target entanglement with painful thoughts and accompanying tendencies of avoiding or running away from experiences [144] [145]. This is achieved through increased psychological flexibility which is 'the ability to fully contact the present moment and the psychological reactions it produces as a conscious person and to persist or change behavior in the situation in the service of chosen values'[4]. ACT sees many forms of painful thoughts, feelings and emotions as a natural consequence of being human, so it takes on the form of trying to increase one's ability to have meaningful activities in their life [140] [128]. Rather than categorizing behaviors as negative or positive, it is better to evaluate them to align with the values and morals of the person[146].

Psychological flexibility as a change mechanism in ACT is achieved through six sub-processes; experiential acceptance, contact with the present moment, defusion, self-as-context, values, and committed action [128]. Josefson also defines these six sub-processes and mentions that they can address diverse difficulties that are relevant to HIV patients [146]. These six core processes are targeted using behavioral and experiential techniques[147].

Using ACT with people living with HIV

Just like any other chronic condition, living with HIV/AIDS evokes painful thoughts, emotions and experiences, thus, self-regulation is necessary in the management of such a condition[115]. ACT is one intervention that has been tried with people living with HIV to normalize experiences and improve mental health and has produced clinically significant outcomes [148]. Acceptability of ACT as an intervention that reduces drinking alcohol (a factor associated with suboptimal adherence) among people living with HIV is high[140]. Moitra conducted a brief acceptance-based behavior therapy rooted in ACT for highly active antiretroviral therapy adherence (HAART). The participants in this study were of an average age of 52 and had an HIV diagnosis for an average of 12.1 years. Their study noted significant changes in adherence as a result of the ACT therapeutic intervention [149]. In another study that aimed at promoting psychological flexibility among HIV-positive women in Nigeria, participants reported better adherence as a strategy to prevent mother-to-child transmission of HIV after being exposed to 3 sessions of ACT. Relatedly, 8 sessions of ACT lasting 60 minutes each were found to have reduced experiences of depression in a cohort of people living with HIV [148]. Contrary, a cross-sectional study that tested psychological flexibility (PF) and intention or non-intentional adherence, found no significant impact of PF on

adherence. This study however had its methodological challenges, necessitating further investigations.

From a deeper analysis, some of the psychological factors that might lead one to not adhere to HIV medications are beliefs and thoughts[149]: some people believe they do not have control over their condition and its treatment options, plus a fearful response to diagnosis can lead someone to let go of wanting to live a value-driven life so they exhibit value-inconsistent behavior. The lack of acceptance of the condition can hinder the ability to accept the realities of a condition and result in improper health-seeking behaviors or thought suppression [149]. All the factors mentioned above are components that ACT tackles. Thus, 'interventions targeting avoidance of both psychological avoidance of distressing experiences and behavioral avoidance of health-promoting behaviors are warranted when dealing with people living with a chronic condition'[149].

Surprisingly, even with promising results on the effectiveness of ACT and other mindfulness-based interventions for improving the mental and physical conditions of people living with HIV, trials have only been done with adults. To my knowledge, no research has tested the effectiveness of ACT with adolescents, specifically targeting to improve adherence with ART although a few studies have been done about other chronic conditions and they are so far promising.

Challenges of using ACT for HIV

Even though research on the use of ACT among people living with HIV is growing, numerous challenges and gaps still exist. Because of the stigma surrounding HIV, some people find it difficult to share personal experiences during ACT sessions without first establishing therapeutic relationships[140]. Accessing those deep emotions became emotionally overwhelming for some people who consider it to be "too much, too soon"[140]. This points to the importance of longer interventions which Graham pointed out is lacking in research on ACT for HIV[128]. Future research and delivery of ACT might have to consider beginning with introducing self-compassion and acceptance before moving into experiential exercises. Moitra further observes that ACT intervention might be too abstract for a low-functioning cohort because of metaphors and experiential examples. For their study, they made sure to connect their main points back to HIV and HAART to avoid confusion [149]. Besides, culturally sensitive interventions are necessary for certain populations rather than an outright ACT program [146]. Adaptation of ACT is required.

The quality of studies on ACT for long-term conditions is also still low which limits ACT's added value over traditional methods [128]. Graham measured the quality of ACT studies in a review using the Psychotherapy Outcome Study Methodology Rating Form (POSMRF) and only 6 of the studies they reviewed scored more than half of the points on the POSMRF. There is a need for high-quality studies if evidence for ACT effectiveness is to be strengthened[128]. Relatedly, Small sample sizes are noticed in research on ACT for HIV. This compromises the generalizability of findings and also affects quality of results[149]. Also in many studies of ACT and HIV, there is a low number of sessions and low-intensity. Yet, we know there is a marked difference in outcomes when the dose of a psychotherapy is higher and more intense [128].

Finally, research on ACT and HIV has been limited to adults, missing out on adolescents as a subset [140]. Research done with adolescents has not specifically targeted HIV related

symptomology and besides, it's been based on small sample sizes[147 149]. RCTs conducted with adolescents are few with most studies being cross-sectional self-repots [44]. Mindfulness interventions for youths also need to be evaluated [135]. Lastly, there is a need to explore research on ACT for adolescents of different ages and in different contexts, this is because insight can be different at different points in adolescence [150].

Using mindfulness-based interventions with adolescents/effectiveness, feasibility and acceptability.

ACT and other mindfulness-based therapies have produced promising clinical outcomes such as; improved medication adherence, mental health, quality of life, pain management and reduced anxiety, depression, and stress when used with young people having chronic conditions[44 151]. These interventions have also been found to be feasible and acceptable for use with and among adolescents[44 45 150].

Faulkner writes that adolescent interventions do not usually have empirical support[152]. It is important to find out the feasibility and acceptability of the interventions we want to apply to problems that adolescents might be facing. They conducted a study looking at psychological flexibility in youth using the DNA-v model and found that over half of the participants felt that they had gained something of value. They also found that psychological inflexibility was reduced at post-intervention and follow-up. They concluded that overall, ACT-based interventions such as DNA-v are feasible and satisfactory to adolescents[152].

Perry-Parrish et al. further emphasize that stressors in adolescents have been linked to negative mental health outcomes. The literature they explore shows that mindful-based therapies are feasible, well-accepted and beneficial for child and adolescent populations. They cite studies where mindfulness-based instructions led to reductions in elevated blood pressure, anxiety, stress and an increase in self-awareness [135].

In an RCT comparing a mindfulness-based stress reduction program for adolescents (MBSR) with treatment as usual (TAU), adolescents in MBSR reported reduced symptoms of anxiety, depression and somatic stress and at the same time also experienced increased self-esteem and sleep quality[44]. The completion rate was also high, meaning it was deemed necessary and acceptable by adolescents. Relatedly, Fortuna tested a cognitive and mindfulness-based therapy for adolescents with co-occurring post-traumatic stress and substance use disorders and significant improvements were observed after 6 weeks of treatment. Adherence to the intervention was high showing acceptance [136]. Woidneck et al. also used ACT for treating Posttraumatic stress (PTS) in adolescents. They looked at ACT's influence on behavioral change and quality of life. In this study, ACT reduced PTS symptomatology, decreased experiential avoidance and increased psychological flexibility. There was also a high rating of treatment acceptability among participants [147].

Hadlandsmyth compared CBT, ACT and a waitlist in treatment of anxiety among young people and found both ACT and CBT to be having superior outcomes compared to waitlist, with some evidence supporting ACT to have a greater effect size. They also found higher completion rates in all groups. The authors however caution that this does not mean ACT is superior to CBT, but rather has promising results[48]. Furthermore, a paper proposing use of ACT in management of diabetes among adolescents note that; cognitive fusion and experiential avoidance promote

suboptimal adherence to daily medication and thus, since ACT targets such problems, it might provide promising outcomes[47].

Relatedly, a study on ACT for youth with neurofibromatosis Type 1 (NF1) and chronic pain, which also included their parents assessed the efficacy and feasibility of a 2-day ACT workshop for this cohort of people. Some of their secondary aims looked to find any changes in pain intensity, pain-related anxiety depression and pain acceptance. The intervention had 3 -5 patients per group, with a 2-hour session over 2 days with a" booster" session after a month and then completed a 3-month post-intervention follow-up questionnaire. The study found that pain interference and pain intensity were significantly lower post-intervention. Parents reported their children's pain acceptance to have increased. Patient satisfaction for the intervention was 3.9/5 and parental satisfaction was 4.6/5. Both patients and parents expressed that it taught them great strategies for dealing with pain[153].

Livheim et al. compared ACT to TAU (school counselling), for adolescents with depression and stress in Australia and Sweden. The study had a good retention rate (66 enrolled and 51 assessed post-intervention). They found greater improvement in depression and stress and also noted a decrease in anxiety. Acceptability was high in both sites, with 91% of participants giving exclusively positive feedback, saying that they would recommend this to a friend and found it valuable. They concluded that ACT interventions can be especially helpful for adolescents[43]. It is also important to note that ACT-based therapies are values-driven, thus interventions that promote values among adolescents can have significant outcomes. Bryan et al. note that adolescents will usually do what their peers are doing, in the hopes of maintaining the same values. So harnessing values in peers can help others get on board, this is where a value-based intervention can be useful [151]. Some other ways ACT can be useful for adolescents include; dealing with rebellion, handling negative social comparisons, developing career aspirations and

Studies that have shown ACT-based therapies to reduce anxiety, depression, and other clinical outcomes among adolescents are many, but literature is still quite young [150]. Besides, almost all effectiveness and acceptance trials of ACT for adolescents have been done in high-resource contexts (developed world) with lesser burdens of disease creating a dearth of literature to support the relevance of ACT in low-resource contexts. Studies testing the effectiveness of ACT among adolescents in low-resource settings are needed. However, ACT should be adapted to fit cultural requirements in such settings. Murrell emphasizes that intervention effectiveness in a different context can only be improved by adaptation [141]. Various studies have also showed that adapted intervention produce better results than imparted interventions[154 150].

Effectiveness of culturally/contextually adapted mindfulness based interventions

Improving the 'congruence' between end users and the psychotherapy is known to increase uptake of such an intervention[41]. Even though there has been a debate on where altering a well-developed intervention to fit 'cultural sensitivity' won't break its core scientific rigor, but such arguments are watered down by the realization that culture influences all circumstances surrounding an individual and thus, treating it as un existent in the interest of keeping a therapy intact is unreasonable[41].

Bernal defines cultural adaptation as the ‘systematic modification of an evidence-based treatment or intervention protocol to consider language, culture and context in such a way that is compatible with the targeted user’s cultural patterns, meanings and values’ [41]. In a meta-analysis that made a direct comparison of culturally adapted psychotherapy vs. un-adapted therapy, adapted therapies were found to be more effective than the un-adapted and authors concluded that adapting psychotherapies is the future[155]. Furthermore, Rose-Clarke notes that; adolescent-targeted interventions developed from high-income countries need to be adapted before use in low-income countries. This should not only target cultural aspects but also the developmental needs of such adolescents[156].

Even when there is growing evidence supporting the relevance of mindfulness and acceptance-based interventions in reducing human suffering, their utilization with ‘non-dominant cultures’ is still limited, calling for cultural adaptation of such interventions[138]. There is a need to ‘understand the context in which a person is experiencing the distress before encouraging value-consistent behaviors’[138]. Murrell et.al adapted ACT for various immigrants in the US and results showed that ACT has better results when adjusted to become culturally competent[157]. Relatedly, Perry explored the effects of a culturally adapted ACT for Turkish-speaking people in the UK. Overall results indicated an improvement in patient outcomes such as reduced depression, but above all, qualitative narratives showed that participants found adapted ACT to be enjoyable, useful and accessible [158]. In a review of a wide range of cultural adaptations of acceptance-based therapies, Rochie supports the notion that psychotherapeutic cultural adaptations are more effective [159].

In conclusion, the current gaps in the literature justify the need to adapt, test and evaluate adolescent-focused psychosocial interventions that are cognizant of ALWHA’s needs, context and values.

Methods

Overall Study Design

To achieve the study objectives, we shall use a sequential multi-methods design. According to Morse[160], a multi-methods study is where two or more related research projects are conducted, each complete in itself to address research questions or hypothesis or program of interest. Such studies may combine qualitative methods, quantitative methods or both and can be implemented concurrently or sequentially with each study phase/ project independently planned and conducted to answer a particular sub-question [160]. We shall conduct the study in two distinct phases: In phase one, we shall use qualitative methods to culturally adapt the intervention and explore its acceptability among users and providers and in phase two, we shall use quantitative methods to evaluate the effectiveness of the adapted intervention on improving mental health outcomes among AWH.

Study Setting

The study will be carried out in Kampala. As a central business district and capital of Uganda, Kampala serves as a destination for diverse groups of people from regions within and outside the country. The diversity makes the setting appropriate for adapting and testing a novice intervention. The estimated population size in Kampala is about 1.65 million although the day population is much higher since people move from surrounding districts (Wakiso, Mukono and Luwero) to work in Kampala[161]. By 2017, HIV prevalence in Kampala was estimated to be 6.9%[79]. Administratively, Kampala is headed by a city authority and is divided into 5 divisions; Kawempe, Central, Makindye, Nakawa and Rubaga, with at least one public health centre in each division. The study will specifically be conducted at the two largest public health centre facilities managed by the city authority (Kisenyi Health Center iv and Kitebi Health Centre iii). These two study sites are preferred because we already have a working relationship with them, have a sizeable number of adolescents in care, distinct adolescent clinics, gazetted facilities where study sessions can be carried out and provide comprehensive HIV care services, plus serving diverse populations with larger catchment areas.

> **Kitebi Health Center III (KTHCIII),** is located in Mutundwe parish, Rubaga division, serving a population of about 4886 people in Mutundwe, Natete, Busega and the neighborhood. The ART clinic at KTHCIII serves approximately 400 ALWHA.
>
> **Kisenyi Health Centre IV (KHCIV)**, is an urban clinic located in Kisenyi, Rubaga division, serving a catchment population of about one million people within the central business district (CBD), mainly traders, refugees, as well as slum dwellers in the neighbourhood. The HIV clinic at KHCIV serves about 590 ALWHA.

Both health centres are open to the public, serve bigger catchment areas compared to other KCCA health centres, are dominated by the urban poor, have approved ART/Tuberculosis(TB) clinics, and manage a cosmopolitan population with a high HIV prevalence (6.9%)[79].

Sub-studies

Sub-study One: Qualitative study.

The purpose of this exploratory qualitative study is to adapt and explore provider's (HCP) and user's (ALWHA) acceptability of an adapted mindfulness based psychosocial intervention. This sub-study will happen in two phases, commencing with culturally adapting ACT-DNA-V as phase one and assessing acceptability of the culturally adapted treatement as phase two.

Adaptation stage

An exploratory qualitative inquiry following a five-step formative method for adapting psychotherapy (FMAP-Hwang 2009)[162] will guide cultural adaptation. FMAP uses a bottom-up approach for culturally adapting psychotherapy. Step1: will involve generating information through collaborating with stakeholders (ALWHA-Young Adolescent Peers, HCPs, psychiatrists, social workers, child and adolescent counsellors and Psychology or counselling students at the master level): This step will commence with a review of the ACT-DNA-V manual and asking stakeholders for their impression regarding the different aspects of the treatment and how best to modify ACT-DNA-V to meet the needs of adolescents in Uganda. To guide the discussions, the three elements of adapting psychological interventions highlighted in the framework for cultural adaptation of psychological interventions (Heim and Kohrt)[163] will be used. These include cultural conceptualization of distress, treatment components and treatment delivery. Step 2: will involve integrating generated information from the stakeholder sessions into the treatment manual. Because not all stakeholders have similar opinions regarding modifications, the most recurring themes will be considered. The research team will then write a new culturally adapted ACT-DNA-V manual following modifications. Step 3: will involve reviewing the culturally adapted manual by stakeholders. Feedback at this level will guide revising of the manual for improvement.

The last two steps of FMAP form the second phase of the Qualitative study (assessing acceptability). Step 4: will be about assessing the acceptability of the culturally adapted ACT-DNA-V among providers and users and Step 5: will involve incorporating feedback from the acceptability assessment into the manual and finalizing the cultural adaptation process.

Exploring the acceptability of the adapted ACT-DNA-V

At this stage in the research, acceptability will be generally defined as; 'the extent to which people delivering or receiving a health care intervention understand it, are satisfied with it, intend to use it and consider it to be appropriate, based on anticipated or experienced cognitive and emotional responses to the intervention'[8 164]. This sub-study will be conducted among HCPs and selected ALWHA one week after exposure to the intervention to address objective one; exploring the acceptability of a mindfulness-based intervention among providers and users. Data will be collected at a one-time point.

Population for sub-study 1 (Exploring acceptability)

Target population

The target population for this sub-study will be HCPs (counsellors) working in public health centers in Kampala and ALWHA's living in Kampala.

Accessible population

HCP's at Kitebi health center (KTHC-iii) working directly with adolescents and ALWHA's attending care at KTHCIII.

Study population

There is a total of 10 HCPs (counsellors) who directly interface with adolescents and a total of 12 ALWHA (15-19 years) attending care at Kitebi HCIII will be considered to be expert patients (They are classified as young adolescent peers-YAPS or adolescent peers-AP at the study sites; these are adolescents living with HIV/AIDS who are knowledgeable about the disease, have confidence and motivation to take control over their health and can influence other adolescents).

Eligibility Criteria for sub-study 1

To be considered in sub-study 1 as a provider, participants should be counsellors working in ART clinics or directly interfacing with ALWHAs at study site one (Kitebi), not participating in adapting the treatment and willing to provide written informed consent.

To be considered in sub-study 1 as a user, the participant should be an expert patient (YAPS and AP) attending care at study site one (Kitebi). These participants should not have participated in adapting therapy and should be willing to provide informed consent. The consolidated guidelines for the prevention and treatment of HIV/AIDS (2020) allow adolescents above 12 years to consent to HIV testing and care[165].

Sampling for sub-study 1

Sample size

Seven (7) HCPs and 8 ALWHAs will be considered for the study. Both groups will be separately exposed to the intervention protocol and then be required to share opinions/perspectives regarding the acceptability and perceived appropriateness of the intervention. According to Moser and Korstjens (2018), exploratory studies require fewer than 10 participants[166]. Groenewald (2004) also emphasize that 2-10 participants are sufficient to reach saturation in a qualitative study[167].

Sampling strategy

Criterion sampling will be used to select participants who meet the pre-determined criterion of importance. For the case of providers, having experience with ALWHAs will be the criteria for selection, while among users, being an expert patient (having mastery over HIV) is the criteria of importance although it will be combined with maximum variation to have a heterogeneous group of participants balanced by age and gender. Criterion sampling is appropriate for an exploratory study [166].

Data collection

Data will be collected using a semi-structured interview with study participants. Interviews are preferred for this sub-study because they provide more details about an individual's understanding, judgement and perception about the domains of interest. Besides, interviews will be purposed to discover subjective meanings and interpretation an individual attach to the intervention.

The design of the interview guide will draw upon the Theoretical Framework of Acceptability (TFA-Sekhon)[8], although questions will be kept open to be able to elicit meanings that participants bring to the experiences.

Domains of Inquiry

The main domain of inquiry for this sub-study is acceptability. We will be exploring participants' experiences with and perceptions of the intervention drawing upon constructs of the TFA. According to Sekhon, TFA can provide a good guide for exploring the acceptability of healthcare interventions. The framework looks at acceptability as multifaceted with a total of seven constructs which include: affective attitude (how an individual feels about the intervention), burden (perceived amount of effort required to participate), perceived effectiveness (the extent to which intervention is perceived as likely to achieve its purpose), ethicality (the extent to which the intervention has a good fit with an individual's value system), intervention coherence (understanding of the intervention and how it works), opportunity cost (the extent to which benefits, or values must be given up to engage in the intervention) and self-efficacy (participant's confidence that they can perform the behavior required)[8]. Besides domains of the TFA, the guides will also permit the exploration of additional emergent themes that are salient to the research.

Data analysis

Thematic analysis will be conducted since it has been widely used in exploratory qualitative studies[168]. Thematic analysis is about identifying of emergent themes through inductive coding informed by theory (TFA) while allowing surprising findings and identification of phenomena not initially expected.

The procedure for data analysis will involve: organizing data, applying codes to textual excerpts that either are consistent with predetermined themes or suggest new emergent categories of phenomena, "testing" emergent understandings of data as analyses proceed, exploring alternative explanations of data (including counter-evidence and inconsistencies), and writing up results [169].

Sub-study Two: Quantitative study

The purpose of this quantitative sub-study is to measure feasibility (actual fit, suitability for everyday use and practicability[170]) and preliminary effectiveness of ACT-DNA-v to address psychosocial/mental health barriers to adherence (depression, anxiety & stigma).

Sub-study design.

An open label randomized controlled study will be used to achieve the sub-study objectives. The design will involve randomly allocating ALWHA's to either the intervention or control group. The intervention group will be exposed to the adapted mindfulness and acceptance-based intervention while the control group will receive standard of care (ART related counselling). At

baseline, data on proximal psychosocial barriers to adherence (depression, anxiety, stigma) and process factors (psychological flexibility) will be collected.

Randomization

Participants will be randomly allocated to the intervention or control group using computer-generated random numbers. A block size of six with equal individuals going to the two arms per block will be considered sufficient to obtain individuals allocated in a ratio of 1:1 in terms of intervention to the control groups.

Allocation concealment

The sequence will be concealed using sealed serialized brown opaque envelopes that will be kept by the independent statistician who is not part of the study.

Allocation implementation

Trained research assistants will pick the envelopes in consecutive ascending order and allocate the participants to the different arms upon opening the envelope.

Blinding:

This will be an open-label randomized study because the staff will be involved in the care of the participants, assessment of the outcome and the nature of the psychosocial interventions to be administered.

Population for Sub-study Two

Target population

The target population for the quantitative sub-study will be ALWHAs (15-19 years of age) living in Kampala.

Accessible Population

ALWHA's (15-19 years of age), receiving care at the selected KCCA health centers (Kisenyi HCIV and Kitebi HCIII) at the time of sampling.

Study population

Approximately 480 ALWHA's (15-19 years of age) actively engaged in care at KHCIV and KTHCIII with clinical records showing viral load measures.

Eligibility criteria

To participate in the Quantitative study, an individual must meet the following criteria.

Inclusion Criteria:

Participant should be 15-19 years of age, diagnosed HIV positive, attending care at Kisenyi Kitebi health center for the last 6 months, on first or second line of treatment, can speak/understand

Luganda or English and willing to provide informed consent/assent. All records will be confirmed via clinic medical charts.

Exclusion criteria:

A participant will not be eligible to be part of the study if he/she plans to move out of the catchment area within six months, or if he/she is participating in another study related to HIV and care improvement.

Sampling for sub-study two

Sample size

The following assumptions are considered for determining the appropriate sample size for the quantitative study.

<u>Assumptions</u>:

$$N = 2\frac{[Z\alpha + Z\beta]^2 S^2}{(\mu1-\mu2)^2}$$

where:

$Z\alpha$ is standard normal value corresponding to level of significance

$Z\beta$ is standard normal value corresponding to power of the study

α=acceptable type 1 error

β= type II error

$\mu1$ is mean in control group and $\mu2$ is mean intervention

$\mu1-\mu2$ =effect size (minimum meaningful difference between the means in the control and intervention groups)

S=standard deviation of the outcome in the control group

N is the sample size per group

$Z\alpha = 1.96$, $Z\beta = 0.84$, $\alpha = 0.05$ in 2 tails, $\beta = 0.20$ (at 80% power).

Considering a study evaluating effectiveness of Acceptance and Commitment Therapy (ACT) on reducing the depressive symptomatology among Swedish and Australian adolescents (Livheim et.al, 2015)[43], the difference in means of the post treatment RADS2 scores between the ACT arm and control arm was 4.09 while the standard deviation in the control arm was 5.13.[43]

Substituting into the formula,

$N = (2(1.96+0.84)^2 x 5.13^2) / (4.09^2)$

N=25.1

N=26 participants in each group

If we consider a possible clustering in any of the two sites and a design effect of 2.0, the new minimum sample size is 26*(2) =52. If we consider a 10% loss to follow-up, the revised sample size will become (52*(1/ (1-0.10)) =58 participants. We, therefore, aim to recruit, **a total of 116 participants** at 80% power of detecting a mean difference of 4.09 or more in the intervention.

Sampling method

To obtain participants for the Quantitative study, convenience sampling will be used at the screening stage, adolescents will be approached for potential enrollment into the study. Only those who consent/assent will be enrolled. Consent from guardians will also be sought for participants below the age of 18.

Data collection and measures

For feasibility assessment: observation, checklists and survey [170] will be used to collect data. The feasibility measures will include; i) enrollment (number of ALWHA's referred, proportion of those who are eligible and the number that consent), ii) attendance (number of sessions attended and completed and reasons for non-attendance iii) Retention in the study (number of participants completing all sessions and assignments) iv) number of assessments completed and missing data and v) self-rated program acceptability (helpful, meaningful, relevant and interesting)[171].

When evaluating the effectiveness of a mindfulness and acceptance-based intervention, standardized survey instruments and document review will be used to collect data at baseline and post-intervention respectively.

Study Schema

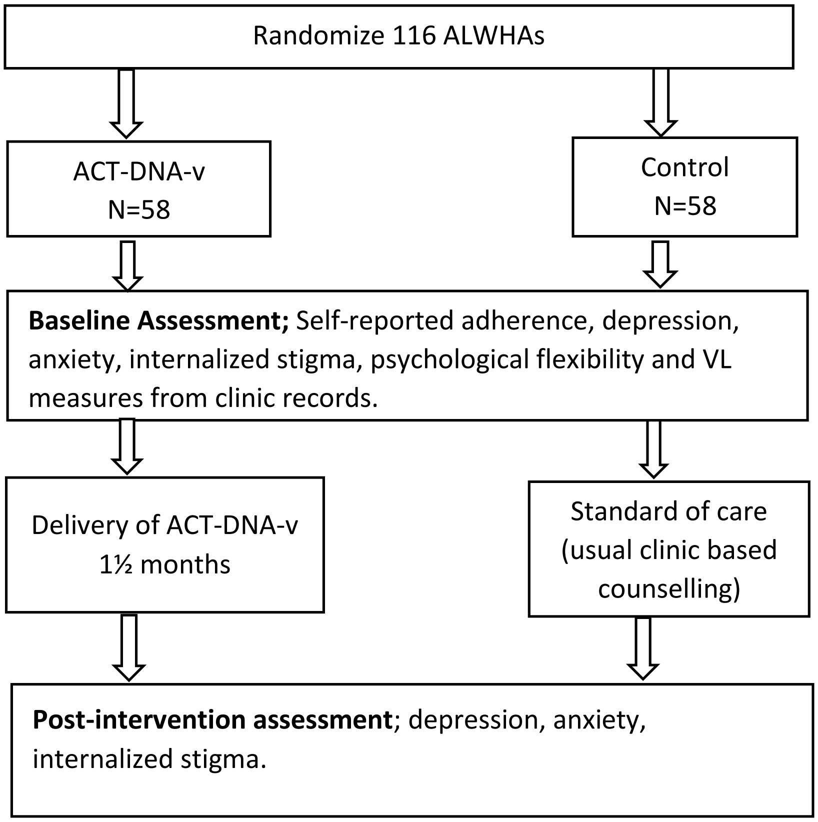

Study instruments

Beck's Depression Inventory ii (BDI ii, Beck et.al, 2006)[172]. BDI-ii is a 21 item self-report rating inventory widely used to measures the presence and degree of depression among adolescents and adults. The BDI-ii includes both cognitive and somatic symptoms of depression. It has a high test retest reliability r=.93 and high internal consistence α=.91[172]. In scoring the BDI-ii, each of the 21 items is summed up to give a single score. Each item has a 4-point scale ranging from 0-3, except items 16&18 that have 7 options indicating either an increase or decrease of appetite and sleep. The total score in the range of 0-13 =minimal range, 14-19=mild, 20-28=moderate and 29-63= severe.[172]. The cut-off guidelines for BDI-ii are adjusted basing on characteristics of the sample[173].

Avoidance and Fusion Questionnaire for youths (AFQ-Y8; Greco et.al, 2008)[175]. This is a measure of psychological inflexibility among children and adolescents. It is an 8-item instrument where participants rate their level of agreement with statements targeting cognitive fusion and experiential avoidance such as "my thoughts and feelings mess up my life". AFQ-Y8 is scored on a 5-point Likert scale made up of a single factor. Scores range from 0-24, with higher scores indicating higher levels of psychological inflexibility. The instrument has shown excellent internal

consistency (α=.90, and test-retest validity r=.80) [176]. It also correlates well with measures of anxiety, depression and anger and is better recommended for use among adolescents[176].

Short health anxiety inventory (SHAI-Salkovskis et.al, 2002)[177]. This is an 18-item measure of health-related anxiety independent of physical health status. It has commonly been used to assess health anxiety among adolescents and has good psychometric properties. The items of SHAI assess worry about health, awareness of body sensessions or changes, and feared consequences of having an illness. Its overall reliability co-efficient is α=.93, then fear of illness (items 1-14) have α=.84 and negative consequences of an illness (items 15-18) α=.67. All items show corrected item-total correlations ≥.30 which is considered acceptable[178]. Responses from the instrument are scored on a 4-point Likert scale, where 0 =no symptom, 1 =mild symptom, 2 =severe symptoms and 3 =very severe symptoms. If more than one statement is selected, the higher-scoring statement is considered. The obtained total score ranges between 0-54 with higher scores representing higher anxiety. A cut-off of 40.5 (total score) distinguishes between non-clinical to severe anxiety[179].

Internalized AIDS-related stigma scale (IARSS-Kalichman 2009)[180]. This is a 6-item scale reflecting self-defeating beliefs and negative perceptions of people living with HIV/AIDS. This instrument has been used and validated in a Ugandan context[181]. Items of the IARSS are assessed with a binary response of YES/NO and a total scale score is computed as the sum of all the 6-items. Higher scores indicate higher levels of internalized HIV/AIDS-related stigma. IARSS has demonstrated acceptable internal consistency (α=.75) and time stability when used in Uganda. Construct validity shows that the scale correlates with related constructs such as depression and mental health-related quality of life[181].

Study intervention

ACT-DNA-v

Uses language, metaphors, mindfulness and experiential learning to encourage young people to defuse distressing psychological experiences and adopt an accepting stance towards one's experience as it unfolds in real-time while pursuing behavioral goals derived from personal life value[149]. This enables them to engage in value-led behaviors such as medication adherence [59].

The intervention involves four sessions delivered in six weeks, with each session lasting two hours a week, for a total of 12 hours. The sessions will be delivered by HCPs (counsellors) together with the research team. All four sessions strengthen the appropriate use of discover, noticer and advisor skills in line with values to improve psychological flexibility.

Table 1; showing sessions of the ACT-DNA-v

Session 1	About DNA-V, being yourself, using social DNA to build groups, the challenge of choosing
Session 2	The Noticer: normalizing noticer, practice awareness, noticer sessions, responding to signals
Session 3	The Advisor: normalizing adviser, helpfulness of advisor, games to unhook from advisor
Session 4	Discoverer: meeting your discoverer, build strength, try it-track it-build it, DNA-V in action
Session 5	Values: small things matter, introduction of values, value is success
Session 6	Who you are, making yourself, ways of well-being, social DNA, and closure.

Outcome measures.

Measured clinical primary outcomes will include Experiences of depression, anxiety and internalized stigma. Then process outcomes will include; Psychological flexibility (cognitive fusion and experiential avoidance) to test the mechanism of change and secondary outcomes will be; self-reported adherence and viral load (obtained from clinical charts).

Table 2; Showing study measures, analysis and outcomes.

**Objective**	**Variables**	**Informant**	**Measures**	**Analysis & Outcomes**
To adapt and measure the feasibility of ACT-DNA-v for use with ALWHA.	*Enrollment<br>*Attendance<br>*Retention in the study.<br>*Completion of sessions and assignments.<br>*Assessments completed and missing data.	ALWHA	*Number of ALWHA's referred, the percentage who are eligible and the number consented.<br>*Number of sessions attended and reasons for non-attendance.<br>*Number of participants who stay in the study till the end (screening baseline, intervention & follow-up) and reasons for withdrawal.<br>*Number of participants completing all assessments (including participant feedback regarding acceptability of data collection methods, duration& content).	*Analysis; Descriptive statistics; summarizing recruitment, demographics, attendance, attrition and intervention adherence.<br>*Outcomes will include; % of participants consented out of those contacted, % of participants retained, % of participants completing all sessions, % of participants completing assignments, % of participants dropping out of study and reasons for withdrawal.

To examine the impact of ACT-DNA-v on reducing proximal psychosocial barriers to medication adherence (depression, anxiety and stigma) among ALWHA	Depression<br>Anxiety<br>Internalized stigma	ALWHA	Qualitative surveys using standardized data collection instruments.<br>* Reynolds Adolescents depression scale (RADS-2)<br>* Social anxiety scale for adolescents-short form (SA-A-SF).<br>* Internalized AIDS-related stigma scale (IARSS-6).	**Analysis**<br>We shall run a T-test, linear regression, and Fisher's exact and Chi-square tests respectively to obtain mean differences between groups.<br><br>**Outcomes** we hypothesize that;<br>*The average means score of ALWHA in the intervention arm should be in the mild depression range of the BDI-ii (14-19) at post-intervention and follow-up, adjusting for baseline compared to the mean score of ALWHA in the control group.<br>* The average mean score of ALWHA in the intervention arm should be below the cutoff score of 40.5 as measured by the SHAI at post-intervention and follow-up.<br>* After dichotomizing the IARSS-6 at median value (0-2 & 3-6), average mean scores of participants in the intervention arm should fall in category1 (0-2) at post-intervention and follow-up.
To measure the effectiveness of a mindfulness-based intervention (ACT-DNA-v) on psychological flexibility among ALWHA in Kampala.	**Process variable**:<br>*Psychological flexibility.	ALWHA	Qualitative surveys using standardized self-administered instruments<br><br>* Avoidance and Fusion Questionnaire for youths (AFQ-Y8).	**Analysis**<br>We shall run a T-test, linear regression, and Fisher's exact and Chi-square tests respectively to obtain mean differences between groups.<br><br>**Outcomes**<br>***The average mean scores of participants in the intervention group will be above the cut-off score on psychological inflexibility using the AFQ-8 at post-intervention and follow-up adjusting for baseline scores.

Statistical analysis

All participant data will be entered into Epidata Manager or RedCap software to facilitate electronic capture of the data. After data cleaning using programmed logic checks and assessment for logic accuracy and consistence, the final database will be locked. The data will be analyzed using the Statistical Package for Social Scientists (SPSS) version 26.0. A table of baseline

characteristics will be drawn with continuous and categorical variables between the intervention and control arm compared using T-test, linear regression, Fisher's exact and Chi-Square tests respectively. A p-value of less than will be considered statistically significant. The individuals will be compared on the outcomes shown in table 2 above of this sub-study following an Intention to treat analysis (ITT) approach. The ITT population will be defined as all randomized patients with data for the outcomes. However, per protocol analysis will also be carried out and results compared with the ITT. The results will be reported following the CONSORT 2010 guidelines[182].

Schedule of activities

	1st Month	2nd Month	3rd month	4th month	5th month	6th month	7th month	8th month	9th month	10th month	11th month
INTERVENTION AND CONTROL GOUPS											
Eligibility Screen	x										
Informed Consent	x										
Adapting and Exploring acceptability of the adapted ACT-DNA-V	x	X									
Recruiting for Quantitative study			X								
Baseline assessment			X								
Post intervention assessment						X					
Assessment at 4 months Follow											x
INTERVENTION GROUP											
Mindfulness and ACT				X							
The noticer-Increasing noticing skill				x							
The advisor-Normalizing experiences				X							
Discoverer-Building resilience					X						
Values clarification-connecting to values					X						
Connecting the disc-bringing it to life					X						
CONTROL GROUP											
Standard Care				x	x						

Intervention fidelity

To ensure that delivery of the intervention adheres to the underlying theoretical premises and the protocol, sessions will be audiotaped and reviewed by two independent raters (ACT experts) to determine if all critical components are delivered. The aim will be to achieve a fidelity rating of at least 85% for all sessions.

Ethical Approval

The study proposal, protocol, informed consent form(s), recruitment materials, and all participant materials will be submitted to the Makerere University School of Medicine Research and Ethics Committee (SOM-REC) for review and approval. Permission to carry out the study will also be sought from the Clinical Epidemiology Unit of the School of Medicine, the Uganda National Council of Science and Technology (UNCST) and the Kampala Capital City Authority (KCCA). Participant's written consent/assent will be sought for ALWHA and HCPs.

***Inclusion of children*:** adolescents aged 14-19 years will be enrolled in the study, therefore minors <18 years of age will be included. Much as the study is considered to be of minimal risk to participants including minors, their written consent/assent will be obtained before enrolling into the study. The consolidated guidelines for the prevention and treatment of HIV in Uganda allow adolescents above 12 years to make personal consent for HIV testing and care. All participants will be given sufficient time to read the assent form in their language and to ask any questions.

Special consideration: adolescents who may become pregnant during the study will be given additional psychosocial support on top of the intervention and standard of care. The team will have a standby counsellor to take care of such special needs.

Confidentiality and Privacy

Given the private nature of the HIV status, confidentiality and privacy will be treated with utmost importance. To protect the identity of participants the use of identifiers such as names, birthdays, specific locations and other potentially participant-specific information that can reveal their identity to people outside the study will be prohibited. Participants will be assigned numbers rather than using names. Secondly, no personally identifiable information from the study will be released to any unauthorized third party and all research activities will be conducted in as private a setting as possible. All information obtained from the study will be recorded using study identification numbers and kept in encrypted computer folders. All computers used will be password-protected. Data collected will strictly be used for study/research purposes only.

Consent procedure and documentation

All consent forms will be translated into the local language (Luganda) and back-translated into English to ensure the correct use of the language. Consent forms will be read aloud to participants by trained staff. The informed consent will describe the purpose of the study, all the procedures involved, and the risks and benefits of participation. Interviewers will ask participants to summarize the study and explain the reasons why they want to participate. Either a signature or a thumbprint (for those who cannot write) will be acceptable to confirm informed consent for participation in the study, in the case of written consent forms.

Strategies for retaining participants in the trial

To be able to retain participants in the trial, the research team will be sending weekly short messages (sms) to participants as study reminders, emphasize benefits of completing sessions during the briefing, schedule sessions on days that are most convenient to participants, provide incentives such as transport refund and breakfast for every session and keep sessions interactive and interesting.

Data Safety and Monitoring Committee (DSMC)

The DSMC will be composed of five independent members including; a psychologist, a physician, a statistician, an epidemiologist and an ethicist.

No investigator directly involved in the care of the subjects will be recruited as a member of the DSMC. All severe adverse events that occur will be notified to the DSMC. The DSMC will meet at midway, three-quarters of the study to ensure the scientific integrity of the study, adherence to the protocol and conduct an interim analysis. The interim analysis may be conducted at the halfway stage of the study or as deemed necessary by the DSMC. Depending on the number of interim analyses, adjustments for the multiple comparisons will be made. The interim analysis P value adjustment will be done using the conservative method of Bonferroni to cater for multiple testing and avoid type 1 errors.

Stopping rules to be applied to the proposed trial will include:

1.) Availability of new efficacious psychosocial intervention when the trial is still ongoing.

2.) When treatment hazards outweigh the benefits of the intervention.

3.) The presence of very strong evidence of a treatment difference with a very small P value.

4.) The futility of the study due to the failure to recruit the required number of participants.

The presence of any or combination of these listed criteria as guided by the DSMC will lead to the stopping of the trial.

Community Engagement Plan

To be able to conduct research that can be beneficial to the intended community, stakeholder engagement will be prioritized. The study will commence with a formative stage that will involve adapting the protocol to the specific needs of the community. Two stakeholder workshops will be conducted. During the workshops, various stakeholders will provide feedback on the approperiateness of language, content, metaphors, and language in the ACT-DNA-v protocol. This feed back will be used to make adjustments to the protocol. We intend to use a deliberative community engagement approach where community members will be offered an opportunity to consider the protocol in depth, challenge each others opinions and reach an informed position on what is appropriate.

COVID-19 Risk Management Plan

The study will be carried out at health centres that have standard operating procedures to be followed as a response to COVID-19. However, on top of the health centre SOPs, we shall take off temperatures before access to the training room, provide hand sanitiser and face masks to all participants, set up a hand washing point outside the premises, have sessions with smaller groups and work from a large training venue to maximize social distancing. In addition to these precautionary measures, it will be a requirement that both researchers and participants present their COVID-19 vaccination cards to the principal investigator ahead of the study.

Publication Plan

The study will result in three publications:

i) Exploring the acceptability of mindfulness and acceptance-based psychosocial support intervention among providers (HCPs) and users (ALWAH).

ii) Assessing the feasibility of an adapted mindfulness and acceptance-based intervention used among ALWHA in Kampala.

iii) Examining the potential of a mindfulness and acceptance-based intervention to reduce the experience of anxiety, depression and internalized stigma among ALWHA in Kampala.

References

1. Hayes SC, Luoma JB, Bond FW, Masuda A, Lillis J. Acceptance and commitment therapy: model, processes and outcomes. *Behav Res Ther.* 2006;44(1):1-25.
2. Hayes L.L, Ciarrochi J.V. *The Thriving Adolescent.* New harbinger; 2015.
3. Organization WH. *Orientation programme on adolessents health for heallth care provider.* Geneva Switzerland 2006.
4. Fletcher L, Hayes SC. Relational frame theory, acceptance and commitment therapy, and a functional analytic definition of mindfulness. *Journal of Rational-Emotive and Cognitive-Behavior Therapy.* 2005;23(4):315-336.
5. Walt G. WHO's World Health Report 2003. *BMJ (Clinical research ed).* 2004;328(7430):6.
6. Morisky DE, Ang A, Krousel-Wood M, Ward HJ. Predictive validity of a medication adherence measure in an outpatient setting. *Journal of clinical hypertension (Greenwich, Conn).* 2008;10(5):348-354.
7. Bishop R.S, Lau M, Shapiro S, et al. Mindfulness: A Proposed Operational Definition. *Clinical Psychology Science and Practice.* 2006;11(3).
8. Sekhon M, Cartwright M, Francis J. *Application of a theoretical framework to assess intervention acceptability: A semi –structured interview study.* 2016.
9. Cassidy S, Okwose N, Scragg J, et al. Assessing the feasibility and acceptability of Changing Health for the management of prediabetes: protocol for a pilot study of a digital behavioural intervention. *Pilot and Feasibility Studies.* 2019;5(1):139.
10. UNAIDS. United Nations Programme on HIVAIDS World AIDS Day Report 2013. In. Geneva Switzerland 2013.
11. Ammon N, Mason S, Corkery JM. Factors impacting antiretroviral therapy adherence among human immunodeficiency virus-positive adolescents in Sub-Saharan Africa: a systematic review. *Public health.* 2018;157:20-31.
12. UNFPA. United Nations Population Fund (UNFPA). Uganda Annual Report, 2017. In:2017.
13. WHO. *The World Health Report 2008 - primary Health Care (Now more than ever).* 2008.
14. UBOS. Uganda Demographic and Health Survey 2016. In: Statisctics, ed. Kampala2016.
15. UPHIA. UGANDA POPULATION-BASED HIV IMPACT ASSESSMENT 2016–2017 SUMMARY SHEET: PRELIMINARY FINDINGS. In:2017.
16. Mafigiri R, Matovu JK, Makumbi FE, et al. HIV prevalence and uptake of HIV/AIDS services among youths (15-24 Years) in fishing and neighboring communities of Kasensero, Rakai District, South Western Uganda. *BMC public health.* 2017;17(1):017-4166.
17. Nabukeera-Barungi N, Elyanu P, Asire B, et al. Adherence to antiretroviral therapy and retention in care for adolescents living with HIV from 10 districts in Uganda. *BMC Infect Dis.* 2015;15(520):015-1265.
18. Adejumo OA, Malee KM, Ryscavage P, Hunter SJ, Taiwo BO. Contemporary issues on the epidemiology and antiretroviral adherence of HIV-infected adolescents in sub-Saharan Africa: a narrative review. *J Int AIDS Soc.* 2015;18(1):20049-20049.
19. Paterson DL, Potoski B, Capitano B. Measurement of adherence to antiretroviral medications. *J Acquir Immune Defic Syndr.* 2002;15(31):00126334-200212153.
20. Soomro N, Fitzgerald G, Seeley J, Schatz E, Nachega J.B, Negin J. Comparison of Antiretroviral Therapy Adherence Among HIV-Infected Older Adults with Younger Adults in Africa: Systematic Review and Meta-analysis. *AIDS Behav.* 2019;23(2):445–458.
21. Inzaule SC, Hamers RL, Kityo C, Rinke de Wit TF, Roura M. Long-Term Antiretroviral Treatment Adherence in HIV-Infected Adolescents and Adults in Uganda: A Qualitative Study. *PLoS One.* 2016;11(11):e0167492-e0167492.

22. Hudelson C, Cluver L. Factors associated with adherence to antiretroviral therapy among adolescents living with HIV/AIDS in low- and middle-income countries: a systematic review. *AIDS Care.* 2015;27(7):805-816.
23. MacCarthy S, Saya U, Samba C, Birungi J, Okoboi S, Linnemayr S. "How am I going to live?": exploring barriers to ART adherence among adolescents and young adults living with HIV in Uganda. *BMC Public Health.* 2018;18(1):1158-1158.
24. Vreeman R.C, McCoy B.M, Lee S. Mental health challenges among adolescents living with HIV. *Journal of the International AIDS Society.* 2017;20.
25. Genberg BL, Lee Y, Rogers WH, Wilson IB. Four types of barriers to adherence of antiretroviral therapy are associated with decreased adherence over time. *AIDS Behav.* 2015;19(1):85-92.
26. Murphy DA, Sarr M, Durako SJ, Moscicki AB, Wilson CM, Muenz LR. Barriers to HAART adherence among human immunodeficiency virus-infected adolescents. *Arch Pediatr Adolesc Med.* 2003;157(3):249-255.
27. Steinberg L. A Social Neuroscience Perspective on Adolescent Risk-Taking. *Dev Rev.* 2008;28(1):78-106.
28. Rana Y, Haberer J, Huang H, et al. Short message service (SMS)-based intervention to improve treatment adherence among HIV-positive youth in Uganda: focus group findings. *PLoS One.* 2015;10(4).
29. Gutgesell M.E, Payne N. Issues of Adolescent Psychological Development in the 21st Century. *Pediatrics in review.* 2004;25 (3):79-85.
30. Bell R. *Psychosocial pathways and health outcomes: Informing action on health inequalities.* London SE1 8UG: PHE publications;2017.
31. Hayes S.C. Acceptance and commitment therapy, relational frame theory, and the third wave of behavioral and cognitive therapies. *Behavior therapy.* 2003;35(4):639-665.
32. Katz IT, Ryu AE, Onuegbu AG, et al. Impact of HIV-related stigma on treatment adherence: systematic review and meta-synthesis. *J Int AIDS Soc.* 2013;16(3 Suppl 2):18640-18640.
33. Michliga G.J, Westergaard R.P, Lama Y, et al. Avoidance, meaning and grief: psychosocial factors influencing engagement in

HIV care. *AIDS Care.* 2018:1360-10451.
34. Kerrigan D, Grieb SM, Ellen J, Sibinga E. Exploring the dynamics of ART adherence in the context of a mindfulness instruction intervention among youth living with HIV in Baltimore, Maryland. *AIDS Care.* 2018;30(11):1400-1405.
35. Linnemayr S, Huang H, Luoto J, et al. Text Messaging for Improving Antiretroviral Therapy Adherence: No Effects After 1 Year in a Randomized Controlled Trial Among Adolescents and Young Adults. *Am J Public Health.* 2017;107(12):1944-1950.
36. Senyonyi R.M, Underwood L.A, Suarez E, Musisi S, Grande T.L. Cognitive behavioral therapy group intervention for HIV transmission risk behavior in perinatally infected adolescents. *Journal of Health.* 2012;4(12):1334-1345.
37. Taddeo D, Egedy M, Frappier JY. Adherence to treatment in adolescents. *Paediatrics & child health.* 2008;13(1):19-24.
38. Nakamanya S, Mayanja BN, Muhumuza R, Bukenya D, Seeley J. Are treatment supporters relevant in long-term Antiretroviral Therapy (ART) adherence? Experiences from a long-term ART cohort in Uganda. *Glob Public Health.* 2019;14(3):469-480.
39. Ssewamala FM, Byansi W, Bahar OS, et al. Suubi+Adherence study protocol: A family economic empowerment intervention addressing HIV treatment adherence for perinatally infected adolescents. *Contemp Clin Trials Commun.* 2019;16:100463-100463.

40. Casale M, Carlqvist A, Cluver L. Recent Interventions to Improve Retention in HIV Care and Adherence to Antiretroviral Treatment Among Adolescents and Youth: A Systematic Review. *AIDS Patient Care STDS.* 2019;33(6):237-252.
41. Bernal G, Jiménez-Chafey MI, Domenech Rodríguez MM. Cultural adaptation of treatments: A resource for considering culture in evidence-based practice. *Professional Psychology: Research and Practice.* 2009;40(4):361-368.
42. Hayes S. C, Luomaa B.J, Bond W.F, Masuda A, Lillis j. Acceptance and Commitment Therapy: Model, processes and outcomes. *Journal of Behaviour Research and Therapy.* 2006;44:1-25.
43. Livheim F, Hayes L, Ghaderi A, et al. The Effectiveness of Acceptance and Commitment Therapy for Adolescent Mental Health: Swedish and Australian Pilot Outcomes. 2015;24(Journal of Child and Family Studies):1016-1030.
44. Biegel G.M, Brown K. W, Shapiro S. L, Schubert C.M. Mindfulness-based stress reduction for the treatment of adolescent psychiatric outpatients: A randomized clinical trial. *Journal of Consulting and Clinical Psychology.* 2009;5(77):855–866.
45. Coyne LW, McHugh L, Martinez ER. Acceptance and commitment therapy (ACT): advances and applications with children, adolescents, and families. *Child Adolesc Psychiatr Clin N Am.* 2011;20(2):379-399.
46. Burckhardt R, Manicavasagar V, Batterham PJ, Hadzi-Pavlovic D, Shand F. Acceptance and commitment therapy universal prevention program for adolescents: a feasibility study. *Child and Adolescent Psychiatry and Mental Health.* 2017;11(1):27.
47. Hadlandsmyth K, White K.S, A.E; N, Greco L. A. Proposing an Acceptance and Commitment Therapy Intervention to Promote Improved Diabetes Management in Adolescents: A Treatment Conceptualization. *International Journal of Behavioral Consultation and Therapy.* 2013;7(4):12-15.
48. Hancock KM, Swain J, Hainsworth CJ, Dixon AL, Koo S, Munro K. Acceptance and Commitment Therapy versus Cognitive Behavior Therapy for Children With Anxiety: Outcomes of a Randomized Controlled Trial. *J Clin Child Adolesc Psychol.* 2018;47(2):296-311.
49. Hayes S.C, Barnes-Holmes.D, Roche B. *Relational frame theory: A post-Skinnerian account of human language and cognition.* Kluwer Academic/Plenum Publishers.; 2001.
50. Hayes S.C. Acceptance and Commitment Therapy, Relational Frame Theory, and the Third Wave of Behavioral and Cognitive Therapies. *Behaviour Therapy.* 2004;35: 639–665.
51. Hayes S. C, Strosahl K. D., Wilson K. G. Acceptance and commitment therapy: An experiential approach to behavior change. 1999.
52. Josefson L. *Acceptance and Commitment Therapy for women affected by HIV, Pain and Sexual abuse. A pilot study in Sierra Leone* [Masters]: Psychology, Uppsala Universitet; 2012.
53. Faezipour M, Ghanbaripanah A, Seyedalinaghi S, Hajiabdolbaghi M, Voltarelli F. Effectiveness of Acceptance and Commitment Therapy on Reducing Depression among People Living with HIV/AIDS. *Journal of International Translational Medicine.* 2018;6:125.
54. Ishola A.G, Chipps J. The use of mobile phones to deliver acceptance and commitment therapy in the prevention of mother–child HIV transmission in Nigeria. *Journal of Telemedicine and Telecare.* 2015;21(8):423–426.
55. Biegel G.M, Brown K.W, Shapiro S.L, Schubert C.M. Mindfulness-Based Stress Reduction for the Treatment of Adolescent Psychiatric Outpatients: A Randomized Clinical Trial. *Journal of Consulting and Clinical Psychology.* 2009;77 855– 866.
56. Fortuna L.R, Porche M.V, Padilla A. A treatment development study of a cognitive and mindfulness-based therapy for adolescents with co-occurring post-traumatic stress and substance use disorder. *Psychology and Psychotherapy.* 2018;91:42–62.

57. Hadlandsmyth K, White S.K, Nesin E. A, Greco A. L. Proposing an Acceptance and Commitment Therapy Intervention to Promote Improved Diabetes Management in Adolescents: A treatment Conceptualization. *Journal of Behavioral Consultation and Therapy.* 2013;7(4):12-15.
58. Woolf-King S.E, Sheinfil A.Z, Babowich J.D, Siedle-Khan B, Loitsch A, Maisto S.A. Acceptance and Commitment Therapy (ACT) for HIV-infected Hazardous Drinkers: A Qualitative Study of Acceptability. *Alcoholism Treatment Quarterly.* 2019;37(3):342–358.
59. Hayes L.L, Ciarrochi J. *The thriving adolescent, Using Acceptance and Commitment Therapy and Postive Psychology to help teens manage emotions, Achieve goals and build connections* Oakland, Canada. : New Harbinger Publications; 2015.
60. Hayes L, Boyd C.P, Sewell J. Acceptance and Commitment Therapy for the Treatment of Adolescent Depression: A Pilot Study in a Psychiatric Outpatient Setting. *Mindfulness* 2011;2:86–94.
61. Armstrong AB, Morrison KL, Twohig MP. A preliminary investigation of acceptance and commitment therapy for adolescent obsessive-compulsive disorder. *Journal of Cognitive Psychotherapy.* 2013;27(2):175–190.
62. UNAIDS. *United Nation Proramme on HIV/AIDS report.* Geneva, Switzerland 2020.
63. Fletcher L, Hayes C.S. Relational Frame Theory, Acceptance and Commitment Therapy, and a Functional Analytic definision of Mindfulness. *Journal of Rational-Emotive & Cognitive-Behavior Therapy.* 2005;23:315-336.
64. MacCarthy S, Mendoza-Graf A, Huang H, Mukasa B, Linnemayr S. Supporting Adolescents to Adhere (SATA): Lessons learned from an intervention to achieve medication adherence targets among youth living with HIV in Uganda. *Child Youth Serv Rev.* 2019;102:56-62.
65. Adejumo O.A, Malee K.M, Ryscavage P, Hunter S.J, B.0 T. Contemporary issues on the epidemiology and antiretroviral adherence of HIV-infected adolescents in sub-Saharan Africa: a narrative review. *International Aids society.* 2015;1(18).
66. Burckhardt R, Manicavasagar V, Batterham PJ, Hadzi-Pavlovic D, Shand F. Acceptance and commitment therapy universal prevention program for adolescents: a feasibility study. *Child Adolesc Psychiatry Ment Health.* 2017;11(27):017-0164.
67. Halliburton A.E, Cooper L.D. Applications and adaptations of Acceptance and Commitment Therapy (ACT) for adolescents. *Journal of Contextual Behavioral Science.* 2015;4:1-11.
68. Livheim F, Hayes L, Ghaderi A, et al. The Effectiveness of Acceptance and Commitment Therapy for Adolescent Mental Health: Swedish and Australian Pilot Outcomes. *Child family studies.* 2015;24:1016–1030.
69. Perry-Parrish C, Copeland-Linder N, Webb L, Sibinga E.M.S. Mindfulness-BasedApproachesfor Children andYouth. *Pediatric Adolescent Health Care.* 2016;46:172-178.
70. Woidneck M. R, Morrison K. L, Twohig M. P. Acceptance and Commitment Therapy for the Treatment of Posttraumatic Stress Among Adolescents. *Journal of Behavior Modification.* 2014;38:451-476.
71. Jones CL, Jensen JD, Scherr CL, Brown NR, Christy K, Weaver J. The Health Belief Model as an explanatory framework in communication research: exploring parallel, serial, and moderated mediation. *Health Commun.* 2015;30(6):566-576.
72. Conner M, Norman P. *Predicting and changing health behaviour. Research and Practice with Social Cognition Models.* Vol Third edition. Berkshire, England: McGraw Hill; 2015.
73. Kumar R, Prinja S, Lakshmi P.V.M. Health Care Seeking Behavior of Adolescents: Comparative Study of Two Service Delivery Models. *Indian Journal of Pediatrics.* 2008;75.
74. Sawyer SM, Afifi RA, Bearinger LH, et al. Adolescence: a foundation for future health. *Lancet (London, England).* 2012;379(9826):1630-1640.

75. Rosenstock IM. Historical Origins of the Health Belief Model. *Health Education Monographs.* 1974;2(4):328-335.
76. *Relational frame theory: A post-Skinnerian account of human language and cognition.* New York, NY, US: Kluwer Academic/Plenum Publishers; 2001.
77. UNICEF. HIV and AIDS in adolescents. . 2020.
78. Commission UA. Presidential fast-track initiative on ending HIV/AIDS in Uganda. . In:2019.
79. UPHIA. *Uganda population-based HIV impact assessment 2016–2017 summary sheet: preliminary findings* 2017.
80. Avert. Global information and eductaion on HIVand AIDS. 2020.
81. Health Mo. CONSOLIDATED GUIDELINES FOR THE PREVENTION AND TREATMENT OF HIV AND AIDS IN UGANDA. In. Uganda2020.
82. Christie D, Viner R. Adolescent development. *BMJ (Clinical research ed).* 2005;330(7486):301-304.
83. WHO. *HIV and adolescents: guidance for HIV testing and counselling and care for adolescents living with HIV.* World Health Organization;2013.
84. Kemnic TR, Gulick PG. HIV Antiretroviral Therapy. In: *StatPearls.* Treasure Island (FL): StatPearls Publishing

Copyright © 2020, StatPearls Publishing LLC.; 2020.
85. Alvi Y, Khalique N, Ahmad A, Khan HS, Faizi N. World Health Organization Dimensions of Adherence to Antiretroviral Therapy: A Study at Antiretroviral Therapy Centre, Aligarh. *Indian journal of community medicine : official publication of Indian Association of Preventive & Social Medicine.* 2019;44(2):118-124.
86. Murray J, Whitehouse K, Ousley J, et al. High levels of viral repression, malnutrition and second-line ART use in adolescents living with HIV: a mixed methods study from Myanmar. *BMC Infect Dis.* 2020;20(1):020-04968.
87. Bijker R, Jiamsakul A, Kityo C, et al. Adherence to antiretroviral therapy for HIV in sub-Saharan Africa and Asia: a comparative analysis of two regional cohorts. *J Int AIDS Soc.* 2017;20(1):21218.
88. Galea JT, Wong M, Muñoz M, et al. Barriers and facilitators to antiretroviral therapy adherence among Peruvian adolescents living with HIV: A qualitative study. *PLoS One.* 2018;13(2).
89. Koirala S, Deuba K, Nampaisan O, Marrone G, Ekström AM. Facilitators and barriers for retention in HIV care between testing and treatment in Asia-A study in Bangladesh, Indonesia, Lao, Nepal, Pakistan, Philippines and Vietnam. *PLoS One.* 2017;12(5).
90. Soomro N, Fitzgerald G, Seeley J, Schatz E, Nachega JB, Negin J. Comparison of Antiretroviral Therapy Adherence Among HIV-Infected Older Adults with Younger Adults in Africa: Systematic Review and Meta-analysis. *AIDS Behav.* 2019;23(2):445-458.
91. Mutwa PR, Van Nuil JI, Asiimwe-Kateera B, et al. Living situation affects adherence to combination antiretroviral therapy in HIV-infected adolescents in Rwanda: a qualitative study. *PLoS One.* 2013;8(4):3.
92. Nabukeera-Barungi N, Elyanu P, Asire B, et al. Adherence to antiretroviral therapy and retention in care for adolescents living with HIV from 10 districts in Uganda. *BMC Infect Dis.* 2015;15(520):015-1265.
93. Wadunde I, Tuhebwe D, Ediau M, Okure G, Mpimbaza A, Wanyenze RK. Factors associated with adherence to antiretroviral therapy among HIV infected children in Kabale district, Uganda: a cross sectional study. *BMC Res Notes.* 2018;11(1):018-3575.
94. Costa JM, Torres TS, Coelho LE, Luz PM. Adherence to antiretroviral therapy for HIV/AIDS in Latin America and the Caribbean: Systematic review and meta-analysis. *J Int AIDS Soc.* 2018;21(1):25066.

95. Nabunya P, Bahar OS, Chen B, Dvalishvili D, Damulira C, Ssewamala FM. The role of family factors in antiretroviral therapy (ART) adherence self-efficacy among HIV-infected adolescents in southern Uganda. *BMC Public health.* 2020;20(1):020-8361.
96. Patel K, Seage GR, 3rd, Burchett SK, Hazra R, Van Dyke RB. Disparities in HIV Viral Suppression Among Adolescents and Young Adults by Perinatal Infection. *Am J Public Health.* 2019;109(7):e9.
97. Dawson L.A. What factors affect adherence to medicines? *Arch Dis Child Educ Pract Ed.* 2019;104:49–52.
98. Cruz ML, Cardoso CA, Darmont MQ, et al. Children and Adolescents with Perinatal HIV-1 Infection: Factors Associated with Adherence to Treatment in the Brazilian Context. *Int J Environ Res Public Health.* 2016;13(6).
99. Olds PK, Kiwanuka JP, Ware NC, Tsai AC, Haberer JE. Explaining antiretroviral therapy adherence success among HIV-infected children in rural Uganda: a qualitative study. *AIDS Behav.* 2015;19(4):584-593.
100. MacCarthy S, Saya U, Samba C, Birungi J, Okoboi S, Linnemayr S. "How am I going to live?": exploring barriers to ART adherence among adolescents and young adults living with HIV in Uganda. *BMC Public health.* 2018;18(1):018-6048.
101. Ashaba S, Cooper-Vince CE, Vořechovská D, et al. Community beliefs, HIV stigma, and depression among adolescents living with HIV in rural Uganda. *African journal of AIDS research : AJAR.* 2019;18(3):169-180.
102. Maskew M, Fox MP, Evans D, et al. Insights into Adherence among a Cohort of Adolescents Aged 12–20 Years in South Africa: Reported Barriers to Antiretroviral Treatment. *AIDS Research and Treatment.* 2016;2016:4161738.
103. MacCarthy S, Saya U, Samba C, Birungi J, Okoboi S, Linnemayr S. How am I going to live?": exploring barriers to ART adherence among adolescents and young adults living with HIV in Uganda. *BMC Public health.* 2018;18(1158).
104. Moucheraud C, Stern AF, Ahearn C, et al. Barriers to HIV Treatment Adherence: A Qualitative Study of Discrepancies Between Perceptions of Patients and Health Providers in Tanzania and Uganda. *AIDS patient care and STDs.* 2019;33(9):406-413.
105. Lee WK, Milloy MJ, Walsh J, Nguyen P, Wood E, Kerr T. Psychosocial factors in adherence to antiretroviral therapy among HIV-positive people who use drugs. *Health Psychol.* 2016;35(3):290-297.
106. Leyva-Moral JM, Loayza-Enriquez BK, Palmieri PA, et al. Adherence to antiretroviral therapy and the associated factors among people living with HIV/AIDS in Northern Peru: a cross-sectional study. *AIDS Res Ther.* 2019;16(1):019-0238.
107. Bukenya D, Mayanja BN, Nakamanya S, Muhumuza R, Seeley J. What causes non-adherence among some individuals on long term antiretroviral therapy? Experiences of individuals with poor viral suppression in Uganda. *AIDS Res Ther.* 2019;16(1):018-0214.
108. Reif LK, Abrams EJ, Arpadi S, et al. Interventions to Improve Antiretroviral Therapy Adherence Among Adolescents and Youth in Low- and Middle-Income Countries: A Systematic Review 2015–2019. *AIDS and Behavior.* 2020;24(10):2797-2810.
109. Natukunda J, Kirabira P, Ong KIC, Shibanuma A, Jimba M. Virologic failure in HIV-positive adolescents with perfect adherence in Uganda: a cross-sectional study. *Tropical Medicine and Health.* 2019;47(1):8.
110. Kemigisha E, Zanoni B, Bruce K, et al. Prevalence of depressive symptoms and associated factors among adolescents living with HIV/AIDS in South Western Uganda. *AIDS Care.* 2019;31(10):1297-1303.
111. Benton TD, Kee Ng WY, Leung D, Canetti A, Karnik N. Depression among Youth Living with HIV/AIDS. *Child Adolesc Psychiatr Clin N Am.* 2019;28(3):447-459.

112. Kim SH, Gerver SM, Fidler S, Ward H. Adherence to antiretroviral therapy in adolescents living with HIV: systematic review and meta-analysis. *Aids.* 2014;28(13):1945-1956.
113. Byakika-Tusiime J, Crane J, Oyugi JH, et al. Longitudinal antiretroviral adherence in HIV+ Ugandan parents and their children initiating HAART in the MTCT-Plus family treatment model: role of depression in declining adherence over time. *AIDS Behav.* 2009;13 Suppl 1:82-91.
114. Zwikker HE, van den Bemt BJ, Vriezekolk JE, van den Ende CH, van Dulmen S. Psychosocial predictors of non-adherence to chronic medication: systematic review of longitudinal studies. *Patient Prefer Adherence.* 2014;8:519-563.
115. Calvetti U.P, Giovelli R.G, Gauer J.G, Duarte de Moraes F.J. Psychosocial factors associated with adherence to treatment and quality of life in people living with HIV/Aids in Brazil. *Brasileiro de psiquiatria.* 2014;63(1):8-15.
116. Hardon A, Davey S, Gerrits T, et al. *From access to adherence: the challenges of antiretroviral treatment. Studies from Botswana, Tanzania and Uganda.* World Health Organization.;2006.
117. Willie TC, Overstreet NM, Sullivan TP, Sikkema KJ, Hansen NB. Barriers to HIV Medication Adherence: Examining Distinct Anxiety and Depression Symptoms among Women Living with HIV Who Experienced Childhood Sexual Abuse. *Behav Med.* 2016;42(2):120-127.
118. Le Prevost M, Arenas-Pinto A, Melvin D, et al. Anxiety and depression symptoms in young people with perinatally acquired HIV and HIV affected young people in England. *AIDS Care.* 2018;30(8):1040-1049.
119. Goffman E. *Stigma: Notes on the management of spoilt identity.* Simon and Schuster; 1986.
120. Kimera E, Vindevogel S, Reynaert D, et al. Experiences and effects of HIV-related stigma among youth living with HIV/AIDS in Western Uganda: A photovoice study. *PLoS One.* 2020;15(4):e0232359.
121. Ben-Zeev D, Young MA, Corrigan PW. DSM-V and the stigma of mental illness. *Journal of mental health (Abingdon, England).* 2010;19(4):318-327.
122. Madiba S, Josiah U. Perceived Stigma and Fear of Unintended Disclosure are Barriers in Medication Adherence in Adolescents with Perinatal HIV in Botswana: A Qualitative Study. *BioMed Research International.* 2019;2019:9623159.
123. Knizek BL, Mugisha J, Osafo J, Kinyanda E. Growing up HIV-positive in Uganda: “psychological immunodeficiency”? A qualitative study. *BMC Psychology.* 2017;5(1):30.
124. Mutumba M, Musiime V, Lepkwoski JM, et al. Examining the relationship between psychological distress and adherence to anti-retroviral therapy among Ugandan adolescents living with HIV. *AIDS Care.* 2016;28(7):807-815.
125. Baron D, Scorgie F, Ramskin L, et al. You talk about problems until you feel free”: South African adolescent girls' and young women’s narratives on the value of HIV prevention peer support clubs. *BMC Public health.* 2020;20(1016):2-13.
126. Okonji EF, Mukumbang FC, Orth Z, Vickerman-Delport SA, Van Wyk B. Psychosocial support interventions for improved adherence and retention in ART care for young people living with HIV (10–24 years): a scoping review. *BMC Public Health.* 2020;20(1):1841.
127. Haberer JE, Sabin L, Amico KR, et al. Improving antiretroviral therapy adherence in resource-limited settings at scale: a discussion of interventions and recommendations. *J Int AIDS Soc.* 2017;20(1).
128. Graham CD, Gouick J, Krahé C, Gillanders D. A systematic review of the use of Acceptance and Commitment Therapy (ACT) in chronic disease and long-term conditions. *Clin Psychol Rev.* 2016;46:46-58.
129. Ssewamala FM, Byansi W, Bahar OS, et al. Suubi+Adherence study protocol: A family economic empowerment intervention addressing HIV treatment adherence for perinatally infected adolescents. *Contemporary clinical trials communications.* 2019;16:100463.

130. Fernandez-Lazaro CI, García-González JM, Adams DP, et al. Adherence to treatment and related factors among patients with chronic conditions in primary care: a cross-sectional study. *BMC Family Practice.* 2019;20(1):132.
131. Rana Y, Haberer J, Huang H, et al. Short message service (SMS)-based intervention to improve treatment adherence among HIV-positive youth in Uganda: focus group findings. *PLoS One.* 2015;10(4):e0125187.
132. Jones C, Hacker D, Cormac I, Meaden A, Irving CB. Cognitive behaviour therapy versus other psychosocial treatments for schizophrenia. *The Cochrane database of systematic reviews.* 2012;4(4):Cd008712.
133. Martin F, Nalukenge W, Lazarus O, Birungi J, Seeley J. "Vital": HIV counselling and testing staff's views of addressing mental health with HIV in Uganda. *BMC Health Services Research.* 2020;20(1):1027.
134. Senyonyi R. Cognitive behavioral therapy group intervention for HIV transmission risk behavior in perinatally infected adolescents. *Health.* 2012;04:1334-1345.
135. Perry-Parrish C, Copeland-Linder N, Webb L, Sibinga EM. Mindfulness-Based Approaches for Children and Youth. *Curr Probl Pediatr Adolesc Health Care.* 2016;46(6):172-178.
136. Fortuna LR, Porche MV, Padilla A. A treatment development study of a cognitive and mindfulness-based therapy for adolescents with co-occurring post-traumatic stress and substance use disorder. *Psychol Psychother.* 2018;91(1):42-62.
137. Hafeman DM, Ostroff AN, Feldman J, et al. Mindfulness-based intervention to decrease mood lability in at-risk youth: Preliminary evidence for changes in resting state functional connectivity. *J Affect Disord.* 2020;276:23-29.
138. Fuchs C, Lee JK, Roemer L, Orsillo SM. Using Mindfulness- and Acceptance-Based Treatments With Clients From Nondominant Cultural and/or Marginalized Backgrounds: Clinical Considerations, Meta-Analysis Findings, and Introduction to the Special Series. *Cognitive and behavioral practice.* 2013;20(1):1-12.
139. Harrison A, Scott W, Timmins L, Graham CD, Harrison AM. Investigating the potentially important role of psychological flexibility in adherence to antiretroviral therapy in people living with HIV. *AIDS Care.* 2020;29:1-10.
140. Woolf-King SE, Sheinfil AZ, Babowich JD, Siedle-Khan B, Loitsch A, Maisto SA. Acceptance and Commitment Therapy (ACT) for HIV-infected Hazardous Drinkers: A Qualitative Study of Acceptability. *Alcohol Treat Q.* 2019;37(3):342-358.
141. Murrell AR, Scherbarth AJ. State of the research & literature address: ACT with children, adolescents and parents. *International Journal of Behavioral Consultation and Therapy.* 2006;2(4):531-543.
142. Masuda A, Hayes SC, Sackett CF, Twohig MP. Cognitive defusion and self-relevant negative thoughts: examining the impact of a ninety year old technique. *Behav Res Ther.* 2004;42(4):477-485.
143. Ciarrochi J, & Blackledge, J. T. Mindfulness-Based Emotional Intelligence Training: A New Approach to Reducing Human Suffering and Promoting Effectiveness. In:2015.
144. Hayes L, Boyd C. P, Sewell J. Acceptance and commitment therapy for the treatment of adolescent depression: A pilot study in a psychiatric outpatient setting. *Mindfulness.* 2011;2(2):86-94.
145. Forman EM, Herbert JD, Moitra E, Yeomans PD, Geller PA. A randomized controlled effectiveness trial of acceptance and commitment therapy and cognitive therapy for anxiety and depression. *Behav Modif.* 2007;31(6):772-799.
146. Josefson L. Acceptance and Commitment Therapy for women affected by HIV, Pain and Sexual abuse. A pilot study in Sierra Leone. *Applied psychology.* 2012.

147. Woidneck M.R, Morrison K.L, Twohig M.P. Acceptance and Commitment Therapy for the Treatment of Posttraumatic Stress Among Adolescents. *Behavior Modification.* 2014;38(4):451-476.
148. FAEZIPOUR M, GHANBARIPANAH A, SEYEDALINAGHI S, HAJIABDOLBAGHI M, VOLTARELLI F. Effectiveness of Acceptance and Commitment Therapy on Reducing Depression among People Living with HIV/AIDS. *International Translational Medicine.* 2018;6(3):125-129.
149. Moitra E, Herbert JD, Forman EM. Acceptance-based behavior therapy to promote HIV medication adherence. *AIDS Care.* 2011;23(12):1660-1667.
150. Halliburton AE, Cooper LD. Applications and adaptations of Acceptance and Commitment Therapy (ACT) for adolescents. *Journal of Contextual Behavioral Science.* 2015;4(1):1-11.
151. Bryan CJ, Yeager DS, Hinojosa CP, et al. Harnessing adolescent values to motivate healthier eating. *Proc Natl Acad Sci U S A.* 2016;113(39):10830-10835.
152. Falkner S, O'Dell S, Golden J. Targeting Psychological Flexibility, sleep hygiene, and physical activity in high school students using DNA-V model. Annual conference for the Association of Contextual Behavioral Science; July 24-29, 2018, 2017; Montréal, Québec, Canada.
153. Martin S, Wolters PL, Toledo-Tamula MA, et al. Acceptance and commitment therapy in youth with neurofibromatosis type 1 (NF1) and chronic pain and their parents: A pilot study of feasibility and preliminary efficacy. *American journal of medical genetics Part A.* 2016;170(6):1462-1470.
154. Clarke K, Patterson P, McDonald FEJ, Wakefield CE, Sansom-Daly U, Zebrack B. The Development and Process Evaluation of a 3-Day Acceptance and Commitment Therapy Group Program for Adolescent Cancer Survivors. *Child & Youth Care Forum.* 2020.
155. Benish SG, Quintana S, Wampold BE. Culturally adapted psychotherapy and the legitimacy of myth: a direct-comparison meta-analysis. *Journal of counseling psychology.* 2011;58(3):279-289.
156. Rose-Clarke K, Pradhan I, Shrestha P, et al. Culturally and developmentally adapting group interpersonal therapy for adolescents with depression in rural Nepal. *BMC Psychology.* 2020;8(1):83.
157. Murrell AR, Rogers L.J. From shy lamb to loaring lion. An acceptance and commitment therapy case study. *Behaviour and social science* 2009(18):81-98.
158. Perry A, Gardener C, Oliver JE, Taş Ç, Özenç C. Exploring the cultural flexibility of the ACT model as an effective therapeutic group intervention for Turkish speaking communities in East London. *The Cognitive Behaviour Therapist.* 2019;12:e2.
159. Roche M.L. Being Mindful About the Assessment of Culture: A Cultural Analysis of Culturally Adapted Acceptance-Based Behavior Therapy Approaches. *Cognitive behavioral practice* 2013(20):60-63.
160. Morse JM. Principles of mixed methods and multi-method research design. In: *Handbook of mixed methods in social and behavioral research* Thousand Oaks, CA: Sage Publication; 2003:189-208.
161. Authority KCC. *Statistical abstract for Kampala City* 2019.
162. Hwang WC. The Formative Method for Adapting Psychotherapy (FMAP): A community-based developmental approach to culturally adapting therapy. *Professional psychology, research and practice.* 2009;40(4):369-377.
163. Heim E, Kohrt B. Cultural Adaptation of Scalable Psychological Interventions: A New Conceptual Framework. *Clinical Psychology in Europe.* 2019;1.
164. Proctor E, Silmere H, Raghavan R, et al. Outcomes for implementation research: conceptual distinctions, measurement challenges, and research agenda. *Administration and policy in mental health.* 2011;38(2):65-76.
165. Ministry of Health. Consolidated guidelines for the prevention and treatment of HIV/AIDS in Uganda. . In. Kampala2020.

166. Moser A, Korstjens I. Series: Practical guidance to qualitative research. Part 3: Sampling, data collection and analysis. *The European journal of general practice.* 2018;24(1):9-18.
167. Groenewald T. A Phenomenological Research Design Illustrated. *International Journal of Qualitative Methods.* 2004;3(1):42-55.
168. Sundler J.A, Lindberg E, Nilsson C, Palmér L. Qualitative thematic analysis based on descriptive phenomenology. *Nursing open.* 2019;6:733-739.
169. Fox N. What is the difference between content analysis and thematic analysis as methods used in qualitative data analysis? 2014.
170. Smith JD, Hasan M. Quantitative approaches for the evaluation of implementation research studies. *Psychiatry Research.* 2020;283:112521.
171. Beck AK, Baker A, Jones S, et al. Exploring the feasibility and acceptability of a recovery-focused group therapy intervention for adults with bipolar disorder: trial protocol. *BMJ Open.* 2018;8(1):e019203.
172. Beck AT, Steer RA, Ball R, Ranieri W. Comparison of Beck Depression Inventories -IA and -II in psychiatric outpatients. *Journal of personality assessment.* 1996;67(3):588-597.
173. Wang YP, Gorenstein C. Psychometric properties of the Beck Depression Inventory-II: a comprehensive review. *Revista brasileira de psiquiatria (Sao Paulo, Brazil : 1999).* 2013;35(4):416-431.
174. De Las Cuevas C, Peñate W. Psychometric properties of the eight-item Morisky Medication Adherence Scale (MMAS-8) in a psychiatric outpatient setting. *Int J Clin Health Psychol.* 2015;15(2):121-129.
175. Greco LA, Lambert W, Baer RA. Psychological inflexibility in childhood and adolescence: Development and evaluation of the Avoidance and Fusion Questionnaire for Youth. *Psychological Assessment.* 2008;20(2):93-102.
176. Jenny Thorsell C, Sandra W, JoAnne D, Gustaf L. A preliminary validation of the Swedish short version of the Avoidance and Fusion Questionnaire for Youth (AFQ-Y8) for children and adolescents with cancer. *Journal of Contextual Behavioral Science.* 2018;10:103-107.
177. Salkovskis PM, Rimes KA, Warwick HM, Clark DM. The Health Anxiety Inventory: development and validation of scales for the measurement of health anxiety and hypochondriasis. *Psychological medicine.* 2002;32(5):843-853.
178. Morales A, Espada JP, Carballo JL, Piqueras JA, Orgilés M. Short health anxiety inventory: factor structure and psychometric properties in Spanish adolescents. *Journal of health psychology.* 2015;20(2):123-131.
179. Arnáez S, García-Soriano G, López-Santiago J, Belloch A. The Spanish validation of the Short Health Anxiety Inventory: Psychometric properties and clinical utility. *International Journal of Clinical and Health Psychology.* 2019;19(3):251-260.
180. Kalichman SC, Simbayi LC, Cloete A, Mthembu PP, Mkhonta RN, Ginindza T. Measuring AIDS stigmas in people living with HIV/AIDS: the Internalized AIDS-Related Stigma Scale. *AIDS Care.* 2009;21(1):87-93.
181. Tsai A.C, Weiser S, Steward W.T, et al. Evidence for the Reliability and Validity of the Internalized AIDS-Related Stigma Scale in Rural Uganda. *AIDS and Behavior* 2012;1(17):427–433.
182. Schulz KF, Altman DG, Moher D, the CG. CONSORT 2010 Statement: updated guidelines for reporting parallel group randomised trials. *BMC Medicine.* 2010;8(1):18.
