# Supplementary material for: Effectiveness of a mindfulness and acceptance-based intervention for improving the mental health of adolescents with HIV in Uganda: An open-label trial
Source: PLoS One. 2024 May 9;19(5):e0301988. doi: 10.1371/journal.pone.0301988 (PMC11081388; doi:10.1371/journal.pone.0301988)
Supplement: Peer review history

# Peer review: Decision Letter 0

**DOI:** 10.1371/journal.pone.0301988.r001

**Authors:** Chiedu Eseadi

2 Jan 2024

PONE-D-23-25851Effectiveness of a mindfulness and acceptance-based intervention for improving the mental health of adolescents with HIV in Uganda: An open-label trialPLOS ONE

Dear Dr. Musanje,

Thank you for submitting your manuscript to PLOS ONE. After careful consideration, we feel that it has merit but does not fully meet PLOS ONE’s publication criteria as it currently stands. Therefore, we invite you to submit a revised version of the manuscript that addresses the points raised during the review process.

Please submit your revised manuscript by Feb 16 2024 11:59PM. If you will need more time than this to complete your revisions, please reply to this message or contact the journal office at plosone@plos.org. When you're ready to submit your revision, log on to https://www.editorialmanager.com/pone/ and select the 'Submissions Needing Revision' folder to locate your manuscript file.

Please include the following items when submitting your revised manuscript:

- A rebuttal letter that responds to each point raised by the academic editor and reviewer(s). You should upload this letter as a separate file labeled 'Response to Reviewers'.
- A marked-up copy of your manuscript that highlights changes made to the original version. You should upload this as a separate file labeled 'Revised Manuscript with Track Changes'.
- An unmarked version of your revised paper without tracked changes. You should upload this as a separate file labeled 'Manuscript'.

If you would like to make changes to your financial disclosure, please include your updated statement in your cover letter. Guidelines for resubmitting your figure files are available below the reviewer comments at the end of this letter.

If applicable, we recommend that you deposit your laboratory protocols in protocols.io to enhance the reproducibility of your results. Protocols.io assigns your protocol its own identifier (DOI) so that it can be cited independently in the future. For instructions see: https://journals.plos.org/plosone/s/submission-guidelines#loc-laboratory-protocols. Additionally, PLOS ONE offers an option for publishing peer-reviewed Lab Protocol articles, which describe protocols hosted on protocols.io. Read more information on sharing protocols at https://plos.org/protocols?utm_medium=editorial-email&utm_source=authorletters&utm_campaign=protocols.

We look forward to receiving your revised manuscript.

Kind regards,

Chiedu Eseadi, PhD

Academic Editor

PLOS ONE

Journal Requirements:

When submitting your revision, we need you to address these additional requirements.

1. Please ensure that your manuscript meets PLOS ONE's style requirements, including those for file naming. The PLOS ONE style templates can be found at

https://journals.plos.org/plosone/s/file?id=wjVg/PLOSOne_formatting_sample_main_body.pdf and

https://journals.plos.org/plosone/s/file?id=ba62/PLOSOne_formatting_sample_title_authors_affiliations.pdf

2. We note that your Data Availability Statement is currently as follows: "All relevant data are within the manuscript and its Supporting Information files."

Please confirm at this time whether or not your submission contains all raw data required to replicate the results of your study. Authors must share the “minimal data set” for their submission. PLOS defines the minimal data set to consist of the data required to replicate all study findings reported in the article, as well as related metadata and methods (https://journals.plos.org/plosone/s/data-availability#loc-minimal-data-set-definition).

For example, authors should submit the following data:

- The values behind the means, standard deviations and other measures reported;

- The values used to build graphs;

- The points extracted from images for analysis.

Authors do not need to submit their entire data set if only a portion of the data was used in the reported study.

If your submission does not contain these data, please either upload them as Supporting Information files or deposit them to a stable, public repository and provide us with the relevant URLs, DOIs, or accession numbers. For a list of recommended repositories, please see https://journals.plos.org/plosone/s/recommended-repositories.

If there are ethical or legal restrictions on sharing a de-identified data set, please explain them in detail (e.g., data contain potentially sensitive information, data are owned by a third-party organization, etc.) and who has imposed them (e.g., an ethics committee). Please also provide contact information for a data access committee, ethics committee, or other institutional body to which data requests may be sent. If data are owned by a third party, please indicate how others may request data access.

[Note: HTML markup is below. Please do not edit.]

Reviewers' comments:

Reviewer's Responses to Questions

**Comments to the Author**

1. Is the manuscript technically sound, and do the data support the conclusions?

The manuscript must describe a technically sound piece of scientific research with data that supports the conclusions. Experiments must have been conducted rigorously, with appropriate controls, replication, and sample sizes. The conclusions must be drawn appropriately based on the data presented. 

Reviewer #1: Yes

Reviewer #2: Yes

Reviewer #3: No

**********

2. Has the statistical analysis been performed appropriately and rigorously? 

Reviewer #1: Yes

Reviewer #2: Yes

Reviewer #3: No

**********

3. Have the authors made all data underlying the findings in their manuscript fully available?

The PLOS Data policy requires authors to make all data underlying the findings described in their manuscript fully available without restriction, with rare exception (please refer to the Data Availability Statement in the manuscript PDF file). The data should be provided as part of the manuscript or its supporting information, or deposited to a public repository. For example, in addition to summary statistics, the data points behind means, medians and variance measures should be available. If there are restrictions on publicly sharing data—e.g. participant privacy or use of data from a third party—those must be specified.

Reviewer #1: Yes

Reviewer #2: Yes

Reviewer #3: Yes

**********

4. Is the manuscript presented in an intelligible fashion and written in standard English?

PLOS ONE does not copyedit accepted manuscripts, so the language in submitted articles must be clear, correct, and unambiguous. Any typographical or grammatical errors should be corrected at revision, so please note any specific errors here.

Reviewer #1: Yes

Reviewer #2: Yes

Reviewer #3: Yes

**********

5. Review Comments to the Author

Please use the space provided to explain your answers to the questions above. You may also include additional comments for the author, including concerns about dual publication, research ethics, or publication ethics. (Please upload your review as an attachment if it exceeds 20,000 characters)

Reviewer #1: dear authors, this is an interesting and innovative study and much needed in the context of support AWH.

My comments are as follows:

A few typos, spaces, please read carefully and fix

Please add a description of the adaptation to Ugandan culture and context. At the moment, I am not clear on that. Did you have a participatory process to adapt it and if yes, how exactly was that done?

Why only 4 sessions: add an explanation to why the local mh experts suggested it and was it based on evidence from other studies?

The sentence around the "14y old clients being young not old" is a bit confusing. Please rephrase, can you add any evidence to your choice of excluding the 14y olds.

Write out abbreviations from time to time (such as DNA-v) for better understanding of the reader

Can you add more information about the trainers. What core competencies did they have?

How do you insure replicability, can the intervention be task-shifted and if yes to whom in the local context?

In standard of care:

How much time on average do/did health care providers spend per adolescent? The program you describe is quite comprehensive and how is fidelity assured?

Tools

Have the BDI ii amd SHAI been validated in your setting? You didnt mention it so I suppose not. You should then discuss this in the limitations.

Discussion

I would appreciate if you had more integration of the program details into the Ugandan culture and context to better understand the applicability of it. It would be good in studies like this to have a qualitative component post intervention to understand the experience of the participants. What barriers did you experience or do you anticipate should such a program be rolled out.

Please also discuss social desirability as a potential bias.

Thank you!

Reviewer #2: PLOS Review

The paper is important and timely. It describes results of a MABI, specifically an ACT-acceptance and commitment therapy model called DNA-v as a way to address mental health distress and life skills of adolescents living with HIV in Uganda. The results are impressive based on four sessions. As authors point out in limitations, an immediate effect is not necessarily sustained. It would be interesting to see if the effects last over time, beyond a four-week period.

Intro: Reads very well, no comments

Methods:

Please clarify the grouping. Were groups mixed gender. How were the sub-groups determined? Did the 3 sub-groups of 4 interact as a bigger group of 11-12? If not, then why did you not randomized into groups of 4 as opposed to 11-12?

Please clarify the DNA-v model v. mindfulness and acceptance. Were both trainers leading different components of the intervention or working together with the three sub-groups during each of the 4 sessions?

What is required to be trained in delivery of the DNA-v model? How is fidelity ensured?

Can you say more as to why in the adaptation phase, 4 sessions were preferred to 6, and what content was cut out to accommodate this change?

For the SHAI. How does the overall reliability have α=.93. When the components are less: The fear of illness sub-scale (items 1-14) has a co-efficient of α=.84 and the negative consequences of an illness sub-scale (items 15-18) has a co-efficient of α=.67?

How were data from youth who were randomized to intervention but never came or missed many sessions handled? Were they still allowed to come for post-intervention visits and where were they grouped? How was missing sessions handled? Were youth allowed to come in any session or was there a criteria for them to attend the first, or a certain number to stay in the group?

Sample size powered for 116, but enrolled 122.

Results:

1. A consort flow diagram might be helpful. More transparency in stating in the text that there was 30% attrition post-assessment? [Doing the math it looks like intervention went from 61 pre to 44 post; control 61 pre to 42 post?]. Were those who did not come for follow up in any way different than those who did? This is somewhat addressed in limitations, but should be addressed in the results section.

2. What was the attendance rate of the intervention group? Did all 44 complete all four sessions?

3. Do you have any HIV RNA viral load data to link the mental health to HIV outcomes?

Discussion:

1. What would need to happen to roll this out in Uganda more broadly? Should it be integrated into clinical care?

2. The discussion statement: line 345 the results can be interpreted that “AWH who received the intervention became open to life experiences non-judgementally, developed self-compassion, and learned how to make choices in ways that bring meaning to them”. I am not sure these conclusions can be made based on the 3 scales of depression, anxiety and stigma presented. Do you have specific data not presented to support this. Otherwise, perhaps it is more accurate to say it is possible that being open to life experiences non-judgmentally, developing self-compassion, and learning how to make choices in ways that bring meaning may be the mechanism by which participants in the intervention arm experienced less mental distress and reduced internal stigma at post-intervention.

3. Any concern of spillover of intervention content between AWH who received the intervention compared to those in the control group given they all attend the same clinic?

4. Consider Simms PMID 34986170, Dow PMID 32887558, in the references of line 392 regarding other psychotherapeutic approaches to improve the MH of adolescents living with HIV in LIC.

Reviewer #3: General comments:

This study reports the results of a mental health intervention for adolescents in Uganda with HIV.

Regarding the study design, I have specific comments below regarding aspects of the study. One major comment I have is, since the intervention was delivered in small groups, I believe this is an individual randomized group treatment trial (IRGT doi: 10.2105/AJPH.2007.127027). In IRGTs, the composition of the groups could influence the participant's outcome; in other words, within group responses may be correlated. The sample size calculations and analyses do not appear to have taken into account this correlation in the analyses. Unfortunately, this is a shortcoming of the power analyses and, depending on the magnitude of the correlation and the small number of subgroups, may mean the study is underpowered.

Regarding the methods, you need to make changes to the analyses to address the correlation between participants in the same subgroup in order for the analyses to be robust. Most likely, this involves switching to a mixed model.

It is also not entirely clear to me whether the regression models analyze change scores or the responses at each survey. There is quite a bit of literature on changes scores and, unfortunately, a good portion of it came out before the widespread availability of methods to fit longitudinal models. The efficiency of change scores can be quite low (e.g., https://doi.org/10.1136/bmj.323.7321.1123). I strongly encourage you not to analyze change scores and instead analyze both pre- and post-test measurements together in a mixed model. That means two rows for each person who completed both assessments and then using a random effect to control for the correlation between those observations.

I also think, if the clinical threshold outcomes are important enough to have a table (Table 6) and devote a portion of the discussion to, multivariable analyses need to be performed on them. It seems strange to not have regression analyses of the clinical threshold outcomes.

The reporting in Table 5 is very confusing (see specific comments below). I also believe more discussion is needed on the magnitude of the decreases. What do the beta coefficients mean? How clinically relevant are these decreases? Continuous scale outcomes are nice from a research perspective because they provide greater power, but I think the changes need to be described more fully.

I think the language of saying that your study "contradicts" other studies (line 351) is too strong.

Specific comments:

1. (line 153) "protocal" should be "protocol".

2. (lines 168-169) Please indicate the program utilized for randomization and the random number algorithm.

3. (line 254) I have many questions about the power analysis. Is the mean decrease of 4.09 based on BDI or all measures? Why did you choose 4.09? What is the relevance of that mean decrease? What did you assume as a variance or standard deviation estimate?

4. (lines 254-255) What did you use for your power calculations, e.g., formulas, software packages, etc.?

5. (lines 262-264) These two statements do not make sense together since an ITT approach assumes anyone randomized gets analyzed. You are following a per protocol (PP) or according to protocol (ATP) approach by excluding all people who withdrew before the end of the protocol.

6. (Table 1) Significance testing in these situations is generally frowned upon because a non-significant p-value does not indicate that groups are the same. For info on the topic in relation to baseline imbalance in randomized trials see Altman, https://doi.org/10.2307/2987510 and Senn, https://doi.org/10.1002/sim.4780131703. My recommendation is to remove the significance testing from table 1 and use standardized difference to assess differences (see Austin, https://doi.org/10.1080/03610910902859574).

7. (Table 4) I don't believe there was a mention of using Cohen's effect size in the methods section. This needs to be described in the methods section. Also, I'm not sure what this adds beyond the information in tables 3 and 5.

8. (Table 5) What is the first row, the one labeled "Coefficient"? Are these the model intercepts? Also, why does the Stigma coefficient not have a CI?

9. (Table 5) Also, the coefficients and CIs are confusing in almost all situations. A first example is the comparison between intervention and control for the anxiety outcome is reported as "-.326 (12.240- (-2.770))" with a p-value of .002. The lower CI limit is greater than the upper CI limit. Since the CI covers zero, the p-value should not be <0.05. A second example is the intervention versus control for the stigma outcome. That is reported as

"-.371 (-1.046 -3.036)". The point estimate is not contained within the CI limits, which is illogical. This table is very problematic and requires a thorough review.

**********

6. PLOS authors have the option to publish the peer review history of their article (what does this mean?). If published, this will include your full peer review and any attached files.

If you choose “no”, your identity will remain anonymous but your review may still be made public.

**Do you want your identity to be public for this peer review?** For information about this choice, including consent withdrawal, please see our Privacy Policy.

Reviewer #1: **Yes: **Dr Ruth Verhey

Reviewer #2: No

Reviewer #3: No

**********

[NOTE: If reviewer comments were submitted as an attachment file, they will be attached to this email and accessible via the submission site. Please log into your account, locate the manuscript record, and check for the action link "View Attachments". If this link does not appear, there are no attachment files.]

While revising your submission, please upload your figure files to the Preflight Analysis and Conversion Engine (PACE) digital diagnostic tool, https://pacev2.apexcovantage.com/. PACE helps ensure that figures meet PLOS requirements. To use PACE, you must first register as a user. Registration is free. Then, login and navigate to the UPLOAD tab, where you will find detailed instructions on how to use the tool. If you encounter any issues or have any questions when using PACE, please email PLOS at figures@plos.org. Please note that Supporting Information files do not need this step.

# Author comment: Author response to Decision Letter 0

**DOI:** 10.1371/journal.pone.0301988.r002

27 Feb 2024

Reviewer 1

a) A few typos, and spaces, please read carefully and fix: The typos and spacing issues have been carefully taken care of

b) Please add a description of the adaptation to Ugandan culture and context. At the moment, I am not clear on that. Did you have a participatory process to adapt it and if yes, how exactly was that done? A description of the adaptation to the Ugandan context has been added to the Introduction (lines 108-114) and Methods (lines 167-171).

c) Why only 4 sessions: add an explanation as to why the local MH experts suggested it and whether was it based on evidence from other studies? Justification for reducing sessions has been included (lines 164-171)

d) The sentence around the "14-year-old clients being young, not old" is a bit confusing. Please rephrase, can you add any evidence to your choice of excluding the 14-year-olds? This has been rephrased (lines 179-189)

e) Write out abbreviations from time to time (such as DNA-v) for a better understanding of the reader. This had been attended to all through the manuscript.

f) Can you add more information about the trainers. What core competencies did they have?

How do you insure replicability, can the intervention be task-shifted and if yes to whom in the local context? Additional information about the trainers has been added. See lines, 230-236 and 

Information on the ability of the intervention to be task-shifted is added. See lines 230-236

g) In standard of care: How much time on average do/did health care providers spend per adolescent? The program you describe is quite comprehensive and how is fidelity assured? Additional information regarding time and fidelity of standard of care has been added. See lines 259-264

Tools

h) Have the BDI ii and SHAI been validated in your setting? You didn't mention it so I suppose not. It would help if you then discussed this in the limitations. The BDI has been validated in Uganda. This information is now included in the manuscript. See lines 285-286. However, the SHAI has not been validated in Uganda. This has been included as a limitation. See lines 574-575

i) Discussion: I would appreciate it if you had more integration of the program details into the Ugandan culture and context to better understand its applicability. It would be good in studies like this to have a qualitative component post-intervention to understand the experience of the participants. What barriers did you experience or do you anticipate should such a program be rolled out? Please also discuss social desirability as a potential bias. Additional details from the adaptation phase that covered the integration of the program into the Ugandan context have been added. See lines 600-609. Social desirability bias has been discussed under the potential limitations of the study. See lines 575-578

Reviewer 2

Intro: 

Reads very well, with no comments. Thank you for the acknowledgement 

Methods:

a) Please clarify the grouping. Were groups mixed gender? How were the sub-groups determined? Did the 3 sub-groups of 4 interact as a bigger group of 11-12? If not, then why did you not randomized into groups of 4 as opposed to 11-12? The sub-groups within the intervention arm were not based on randomization but rather created as sub-divisions balanced by age and gender. See lines 237-241. Each sub-group was made up of 11-13 participants to fit the recommended group size in acceptance and commitment therapy-based programs. This has been clarified (lines 238-240) 

b) Please clarify the DNA-v model v. mindfulness and acceptance. Were both trainers leading different components of the intervention or working together with the three sub-groups during each of the 4 sessions? The relationship between DNA-v and Mindfulness and acceptance has been clarified. See lines 100-102

Trainers collaboratively worked together to deliver the intervention sessions across sub-groups. See line 246 

c) What is required to be trained in the delivery of the DNA-v model? How is fidelity ensured? The minimal requirements to deliver DNA-V sessions and how fidelity is ensured have been included. See lines 230-236

d) Can you say more as to why in the adaptation phase, 4 sessions were preferred to 6, and what content was cut out to accommodate this change? Additional information has been provided. See lines 166-177 

e) For the SHAI. How does the overall reliability have α=.93. When the components are less: The fear of illness sub-scale (items 1-14) has a co-efficient of α=.84 and the negative consequences of an illness sub-scale (items 15-18) has a co-efficient of α=.67? This was an oversight and has been revised. See line 291

f) How were data from youth who were randomized to intervention but never came or missed many sessions handled? Were they still allowed to come for post-intervention visits and where were they grouped? How were missing sessions handled? Were youth allowed to come in any session or was there a criteria for them to attend the first, or a certain number to stay in the group? Only adolescents who completed at least 75% of the program (3/4) were considered for post-assessment. 75% is minimum attendance for the program to offer effect. See lines 248-251.

g) Sample size powered for 116, but enrolled 122.The sample was inflated to cover possible intergroup correlations 

Results:

a). A consort flow diagram might be helpful. More transparency in stating in the text that there was 30% attrition post-assessment. [Doing the math it looks like intervention went from 61 pre to 44 post; control 61 pre to 42 post?]. Were those who did not come for follow-up in any way different from those who did? This is somewhat addressed in the limitations but should be addressed in the results section. A consort flow diagram is the figure being reported on line 225. The 30% attrition has been reported under limitations. See lines 587

b) What was the attendance rate of the intervention group? Did all 44 complete all four sessions? All 44 completed at least 75% of the program. See lines 248-251 

c) Do you have any HIV RNA viral load data to link mental health to HIV outcomes? We did not consider viral load due to the short duration of the study. At the clinic, VL data is collected every six months

Discussion:

a) What would need to happen to roll this out in Uganda more broadly? Should it be integrated into clinical care? Strategies to translate the DNA-v into real clinical practice have been added. See lines 621-625

b) The discussion statement: line 345 the results can be interpreted that “AWH who received the intervention became open to life experiences non-judgementally, developed self-compassion, and learned how to make choices in ways that bring meaning to them”. I am not sure these conclusions can be made based on the 3 scales of depression, anxiety and stigma presented. Do you have specific data not presented to support this? Otherwise, perhaps it is more accurate to say it is possible that being open to life experiences non-judgmentally, developing self-compassion, and learning how to make choices in ways that bring meaning may be the mechanism by which participants in the intervention arm experienced less mental distress and reduced internal stigma at post-intervention. Thank you for the observation. This has been revised. See lines 491-494

c) Any concern of spillover of intervention content between AWH who received the intervention compared to those in the control group given they all attend the same clinic? Spillover was possible and has been acknowledged alongside strategies that were taken to minimize it. See lines 215-220

d) Consider Simms PMID 34986170, and Dow PMID 32887558, in the references of line 392 regarding other psychotherapeutic approaches to improve the MH of adolescents living with HIV in LIC. Thank you for these valuable references, they have been reviewed and considered. Line 546. Also, see references 70 and 71

Reviewer 3

a) Regarding the study design, I have specific comments below regarding aspects of the study. One major comment I have is since the intervention was delivered in small groups, I believe this is an individual randomized group treatment trial (IRGT doi: 10.2105/AJPH.2007.127027). In IRGTs, the composition of the groups could influence the participant's outcome; in other words, within-group responses may be correlated. The sample size calculations and analyses do not appear to have taken into account this correlation in the analyses. Unfortunately, this is a shortcoming of the power analyses and, depending on the magnitude of the correlation and the small number of subgroups, may mean the study is underpowered. Thank you for this observation. Yes, the randomization was individual and treatment given as groups. For the power of the study, we have discussed it as a limitation see lines 580-586 and for analysis we have switched to a mixed effect regression which accounts for within group correlations see lines 347-356

b) Regarding the methods, you need to make changes to the analyses to address the correlation between participants in the same subgroup in order for the analyses to be robust. Most likely, this involves switching to a mixed model. Thank you for the comment. For the correlations between the outcomes, we have remodeled the data based on mixed effects regression see lines 386-451

c) It is also not entirely clear to me whether the regression models analyze change scores or the responses at each survey. There is quite a bit of literature on changes scores and, unfortunately, a good portion of it came out before the widespread availability of methods to fit longitudinal models. The efficiency of change scores can be quite low (e.g., https://doi.org/10.1136/bmj.323.7321.1123). I strongly encourage you not to analyze change scores and instead analyze both pre- and post-test measurements together in a mixed model. That means two rows for each person who completed both assessments and then using a random effect to control for the correlation between those observations. Thank you for your comment. We agree that a mixed effects regression was the best analysis. See lines 414-423

d) I also think, that if the clinical threshold outcomes are important enough to have a table (Table 6) and devote a portion of the discussion to, multivariable analyses need to be performed on them. It seems strange to not have regression analyses of the clinical threshold outcomes. Thank you for the observation. We agree that the central message of the manuscript is centered around the intervention and its effects on the continuous outcomes of depression, stigma, and anxiety. Clinical thresholds were not the central message to deserve much attention. The clinical threshold table has been deleted (line 473) and also removed from the discussion (lines 518-534)

e) The reporting in Table 5 is very confusing (see specific comments below). I also believe more discussion is needed on the magnitude of the decreases. What do the beta coefficients mean? How clinically relevant are these decreases? Continuous scale outcomes are nice from a research perspective because they provide greater power, but I think the changes need to be described more fully. Table 5 has been replaced, the mixed effect model gives a clear magnitude of decrease and have been well documented. See lines 414-451

f) I think the language of saying that your study "contradicts" other studies (line 351) is too strong. The language has been revised. See line 501

g) (line 153) "protocal" should be "protocol". This has been revised. See line 193

h) (lines 168-169) Please indicate the program utilized for randomization and the random number algorithm. We used STATA code based on random numbers and considered block sizes of 6 individuals with equal numbers in both the intervention and control groups. We generated 21 blocks of six individuals. Line 211

i) (line 254) I have many questions about the power analysis. Is the mean decrease of 4.09 based on BDI or all measures? Why did you choose 4.09? What is the relevance of that mean decrease? What did you assume as a variance or standard deviation estimate? A detailed procedure followed in determining sample size has been included. See lines 322-342

j) (lines 254-255) What did you use for your power calculations, e.g., formulas, software packages, etc.? We used a formula and it has been added. Lines 322-342

k) (lines 262-264) These two statements do not make sense together since an ITT approach assumes anyone randomized gets analyzed. You are following a per-protocol (PP) or according-to-protocol (ATP) approach by excluding all people who withdrew before the end of the protocol. Thank you for this observation, this has been revised. See line 356

l) (Table 1) Significance testing in these situations is generally frowned upon because a non-significant p-value does not indicate that groups are the same. For info on the topic in relation to baseline imbalance in randomized trials see Altman, https://doi.org/10.2307/2987510 and Senn, https://doi.org/10.1002/sim.4780131703. My recommendation is to remove the significance testing from Table 1 and use standardized differences to assess differences (see Austin, https://doi.org/10.1080/03610910902859574). Significance testing has been removed from Table 1 (lines 381). Detailed information on groups is presented in mixed effects results 

m) (Table 4) I don't believe there was a mention of using Cohen's effect size in the methods section. This needs to be described in the methods section. Also, I'm not sure what this adds beyond the information in Tables 3 and 5. This table has been deleted (lines 409) by switching to mixed effect regression, information about the precision of the effect has been provided by the confidence intervals. In tables 3,4 and 5 (lines, 430, 439 and 449 respectively)

n) (Table 5) What is the first row, the one labelled "Coefficient"? Are these the model intercepts? Also, why does the Stigma coefficient not have a CI? Thank you for the comment, Table 5 has been removed as it had become redundant with the new analysis 

o) (Table 5) Also, the coefficients and CIs are confusing in almost all situations. A first example is the comparison between intervention and control for the anxiety outcome is reported as "-.326 (12.240- (-2.770))" with a p-value of .002. The lower CI limit is greater than the upper CI limit. Since the CI covers zero, the p-value should not be <0.05. A second example is the intervention versus control for the stigma outcome. That is reported as

"-.371 (-1.046 -3.036)". The point estimate is not contained within the CI limits, which is illogical. This table is very problematic and requires a thorough review. Thank you for the comment, Table 5 has been removed because it had become redundant

Attachment Submitted filename: Response to reviewers.docx

# Editor report: Decision Letter 1

**DOI:** 10.1371/journal.pone.0301988.r003

**Authors:** Chiedu Eseadi

27 Mar 2024

Effectiveness of a mindfulness and acceptance-based intervention for improving the mental health of adolescents with HIV in Uganda: An open-label trial

PONE-D-23-25851R1

Dear Dr. Musanje,

We’re pleased to inform you that your manuscript has been judged scientifically suitable for publication and will be formally accepted for publication once it meets all outstanding technical requirements.

Within one week, you’ll receive an e-mail detailing the required amendments. When these have been addressed, you’ll receive a formal acceptance letter and your manuscript will be scheduled for publication.

An invoice will be generated when your article is formally accepted. Please note, if your institution has a publishing partnership with PLOS and your article meets the relevant criteria, all or part of your publication costs will be covered. Please make sure your user information is up-to-date by logging into Editorial Manager at Editorial Manager® and clicking the ‘Update My Information' link at the top of the page. If you have any questions relating to publication charges, please contact our Author Billing department directly at authorbilling@plos.org.

If your institution or institutions have a press office, please notify them about your upcoming paper to help maximize its impact. If they’ll be preparing press materials, please inform our press team as soon as possible -- no later than 48 hours after receiving the formal acceptance. Your manuscript will remain under strict press embargo until 2 pm Eastern Time on the date of publication. For more information, please contact onepress@plos.org.

Kind regards,

Chiedu Eseadi, PhD

Academic Editor

PLOS ONE

Additional Editor Comments (optional):

Thank you for taking the time to thoroughly respond to the reviewers' comments. 

Reviewers' comments:

# Editor report: Acceptance letter

**DOI:** 10.1371/journal.pone.0301988.r004

**Authors:** Chiedu Eseadi

29 Apr 2024

PONE-D-23-25851R1 

PLOS ONE

Dear Dr. Musanje, 

I'm pleased to inform you that your manuscript has been deemed suitable for publication in PLOS ONE. Congratulations! Your manuscript is now being handed over to our production team.

At this stage, our production department will prepare your paper for publication. This includes ensuring the following:

* All references, tables, and figures are properly cited

* All relevant supporting information is included in the manuscript submission,

* There are no issues that prevent the paper from being properly typeset

If revisions are needed, the production department will contact you directly to resolve them. If no revisions are needed, you will receive an email when the publication date has been set. At this time, we do not offer pre-publication proofs to authors during production of the accepted work. Please keep in mind that we are working through a large volume of accepted articles, so please give us a few weeks to review your paper and let you know the next and final steps. 

Lastly, if your institution or institutions have a press office, please let them know about your upcoming paper now to help maximize its impact. If they'll be preparing press materials, please inform our press team within the next 48 hours. Your manuscript will remain under strict press embargo until 2 pm Eastern Time on the date of publication. For more information, please contact onepress@plos.org.

If we can help with anything else, please email us at customercare@plos.org.

Thank you for submitting your work to PLOS ONE and supporting open access. 

Kind regards, 

PLOS ONE Editorial Office Staff

on behalf of

Dr. Chiedu Eseadi 

Academic Editor

PLOS ONE
